# Enriched rhizospheric functional microbiome may enhance adaptability of *Artemisia lavandulaefolia* and *Betula luminifera* in antimony mining areas

**DOI:** 10.3389/fmicb.2024.1348054

**Published:** 2024-03-21

**Authors:** Wenli Xing, Xu Gai, Liang Xue, Shaocui Li, Xiaoping Zhang, Feng Ju, Guangcai Chen

**Affiliations:** ^1^Research Institute of Subtropical Forestry, Chinese Academy of Forestry, Hangzhou, China; ^2^China National Bamboo Research Center, Key Laboratory of State Forestry and Grassland Administration on Bamboo Forest Ecology and Resource Utilization, Hangzhou, Zhejiang, China; ^3^Key Laboratory of Coastal Environment and Resources of Zhejiang Province, School of Engineering, Westlake University, Hangzhou, China

**Keywords:** rhizosphere microbiome, Sb mining sites, *Betula luminifera*, *Artemisia lavandulaefolia*, ecological restoration

## Abstract

Dominant native plants are crucial for vegetation reconstruction and ecological restoration of mining areas, though their adaptation mechanisms in stressful environments are unclear. This study focuses on the interactions between dominant indigenous species in antimony (Sb) mining area, *Artemisia lavandulaefolia* and *Betula luminifera*, and the microbes in their rhizosphere. The rhizosphere microbial diversity and potential functions of both plants were analyzed through the utilization of 16S, ITS sequencing, and metabarcoding analysis. The results revealed that soil environmental factors, rather than plant species, had a more significant impact on the composition of the rhizosphere microbial community. Soil pH and moisture significantly affected microbial biomarkers and keystone species. *Actinobacteria*, *Proteobacteria* and *Acidobacteriota*, exhibited high resistance to Sb and As, and played a crucial role in the cycling of carbon, nitrogen (N), phosphorus (P), and sulfur (S). The genes participating in N, P, and S cycling exhibited metabolic coupling with those genes associated with Sb and As resistance, which might have enhanced the rhizosphere microbes’ capacity to endure environmental stressors. The enrichment of these rhizosphere functional microbes is the combined result of dispersal limitations and deterministic assembly processes. Notably, the genes related to quorum sensing, the type III secretion system, and chemotaxis systems were significantly enriched in the rhizosphere of plants, especially in *B. luminifera*, in the mining area. The phylogenetic tree derived from the evolutionary relationships among rhizosphere microbial and chloroplast whole-genome resequencing results, infers both species especially *B. luminifera*, may have undergone co-evolution with rhizosphere microorganisms in mining areas. These findings offer valuable insights into the dominant native rhizosphere microorganisms that facilitate plant adaptation to environmental stress in mining areas, thereby shedding light on potential strategies for ecological restoration in such environments.

## Graphical abstract

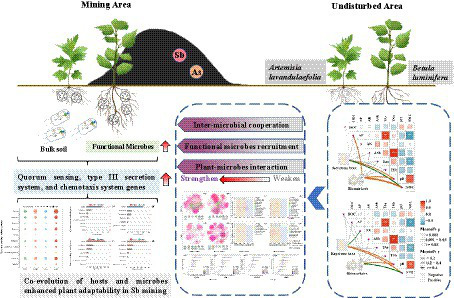

## 1 Introduction

Antimony (Sb) pollution has merged as a major public health concern worldwide, which generally resulted from mining and smelting activities, damaging the local ecosystem, and threatening ecological health and safety (Wilson et al., 2010; Li Y. et al., 2021). About 80% of the world’s Sb supply originating from China (He et al., 2012), and there is an imminent need for ecological restoration in Sb mining areas. Vegetation reconstruction with indigenous pioneer plants plays a crucial role in ecological restoration, which initiates the recovery of soil structure, fertility, microorganisms, and animals, thereby facilitating the rebuilding of the ecosystem’s structure and function in mining areas (Hu et al., 2012). The indigenous pioneer plants are typically well-adapted to local environment and can tolerate disturbances effectively, enabling them to establish vegetation rapidly and efficiently in mining damaged areas (Gairola et al., 2023). It’s found rhizosphere-associated microbes, by promoting the growth of local dominant plants such as *Miscanthus sinensis*, enhance their ecological adaptability in extreme environments like metal pollution (Sun et al., 2021; Li Y. et al., 2022). Additionally, studies on the rhizosphere microbial community of alkaline iron tailings revealed that these microbes have evolved a range of survival strategies to adapt to the mining environment characterized by nutrient deficiency and metal pollution. For instance, the reductive tricarboxylic acid cycle (rTCA) enhances the carbon sequestration capacity of rhizosphere microbes in tailings (Geng et al., 2022). However, the study on rhizosphere microbes associated with dominant woody plants in mining areas remains relatively limited.

Plants grown in contaminated sites may recruit more rhizosphere microorganisms related to stress tolerance and growth promotion from the bulk soil through enhanced root exudates (Hu et al., 2018; Chai and Schachtman, 2022). It is widely believed this microbial community assembly is determined by niche filtration, i.e., the selective effect of plant species on root-related microorganisms (Ling et al., 2022; Zhong et al., 2022). Nevertheless, soil characteristics and stress events are suggested as the primary driving forces of plant-microbial community interactions under stressful conditions (Escudero-Martinez and Bulgarelli, 2019; Xing et al., 2023). The microorganisms related to Sb-resistance, carbon, nitrogen, phosphorus and sulfur cycling in Sb mining sites have been suggested to promote the bioremediation of Sb pollution (Sun et al., 2017; Li Y. et al., 2021). The recruitment of these rhizosphere microorganisms can strengthen the connection between plants and soil ecosystem processes, promote the formation of specific rhizosphere system (Park et al., 2023), and enhance the nutrient cycle, metal resistance, and establishment of plant communities (Bhuyan et al., 2020). Hence, the dominant indigenous plants may benefit from the supplement and assembly of closely related microorganisms that experience the same antimony stress environment. Typically, plant roots recruit beneficial microorganisms through a ‘cry for help’ strategy (Gao et al., 2021; Li et al., 2022), where they secrete substances that interfere with microbial quorum sensing, and some nearby microorganisms can use their unique chemotaxis system to sense the release of plant signals and regulate the movement toward the plant (Badri et al., 2009; Raina et al., 2019). Microorganisms secrete effector proteins to overcome host immune surveillance in the vicinity of plant roots (Trivedi et al., 2020), which is essential for symbiotic microorganisms to colonize different plant niches (Ansari and Ahmad, 2018). These proteins are typically secreted through a secretion pathway or type III secretion system (T3SS) (Trivedi et al., 2020). To date, the recruitment of microorganisms by plants mainly focuses on the signal transduction and action mechanism at the molecular level (Sasse et al., 2018; Liu et al., 2021), while the adaptive mechanism of dominant indigenous plants in polluted or ecologically destroyed areas is still unclear. Meanwhile, the complex soil conditions in mining areas make it difficult to conduct control studies of dominant indigenous plants and their associated rhizosphere microorganisms, which has led to a lack of clarity regarding plant-microorganism interactions in such areas.

It has been suggested that *Betula luminifera,* the only woody plant that grows in Sb mining sites, along with the herbaceous *Artemisia lavandulaefolia,* are the dominant indigenous plants in Sb mining area in Southwest China (Du et al., 2023). These plants are able to thrive in the severely polluted environment around mining sites (the total antimony content is 27,592.33 ± 501.58 mg·kg^−1^, and the available antimony content is 44.03 ± 45.02 mg·kg^−1^). However, it is not clear how these plants can tolerate and grow well in such harsh conditions. We hypothesized that, (1) the rhizosphere of plants significantly enriches certain microorganisms under the stress conditions of the mining area, which may respond to “cry for help” from plants through evolution; (2) The functional microorganisms enriched in the rhizosphere of mining areas may exhibit higher functional redundancy, and the co-evolution between plants and microorganisms may enhance the long-term adaptability of plants in the mining area. These findings provide insights into the potential adaptive mechanisms of plants in mining areas from the perspective of rhizosphere microorganisms, and offer valuable evidence for promoting ecological restoration processes in antimony mining regions.

## 2 Materials and methods

### 2.1 Experimental design and samples collection

**Figure 1:**
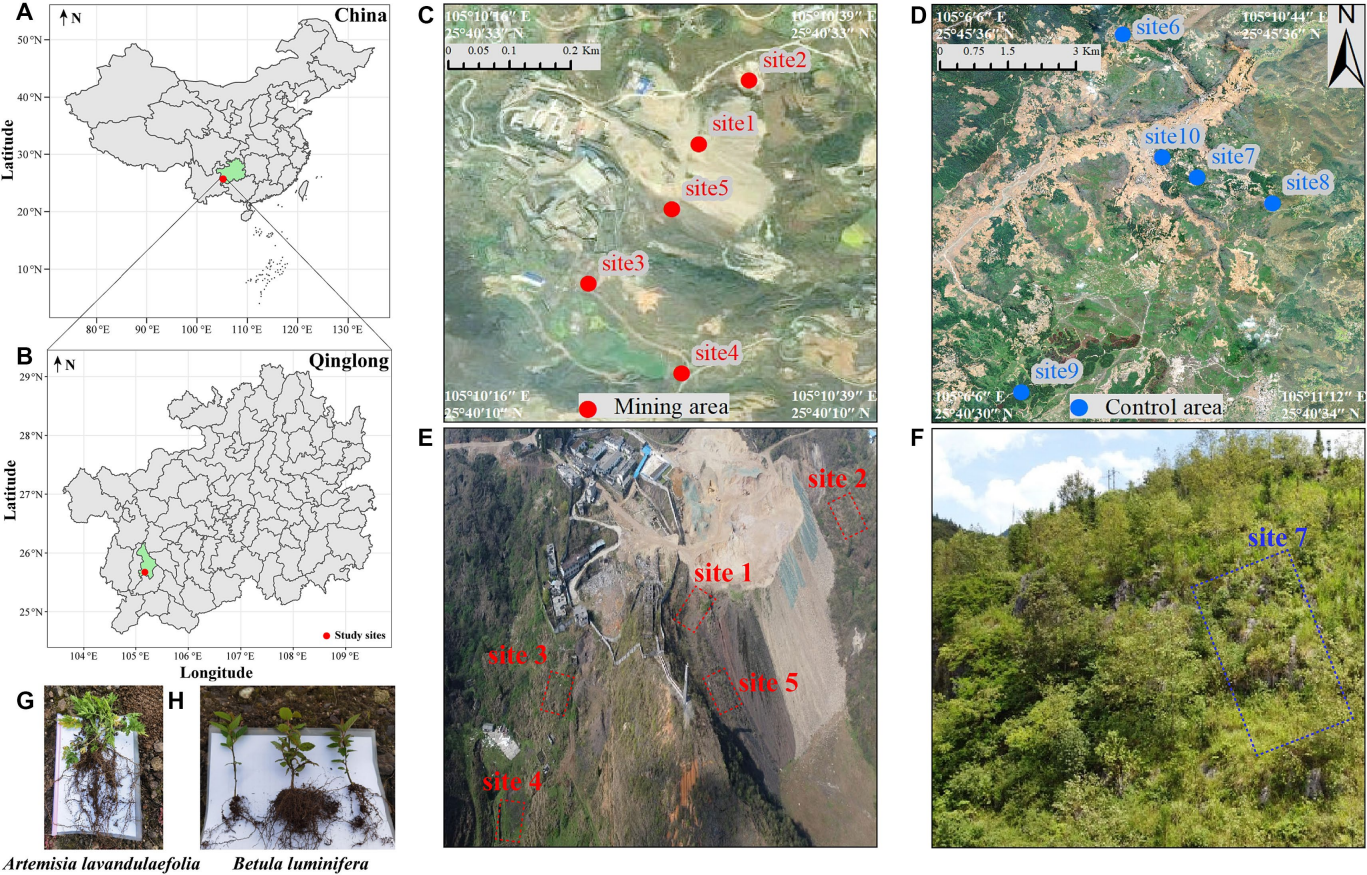
Sketch map of study area and sample collection points. Study sites **(A,B)**, sampling sites in mining area **(C)** and control area **(D)**, the aerial map of mining area **(E)**, and the aerial map of site 7 in the control area **(F)**. *Artemisia lavandulaefolia* **(G)** and *Betula luminifera* **(H)** with a height of 8–10 cm. The minimum distance between the sampling points in control area and mining area is 24 km. Due to the large area of control area and the long distance between sampling point, the aerial photos of site 7 in control area were randomly selected to display.

In March 2021, five representative ecological damaged sites in antimony mining area (mining area) and five control sites without mining activities interference (control area) were selected (Figure 1). The control area located more than 20 km away from the antimony mining area, and is defined as a natural succession area without human interference, while the mining area is defined as a high pollution area. In each plot, we randomly selected 30 individuals of *B. luminifera* (one-year-old seedling after germination, the height of the plants was 8–10 cm) and 30 of *A. lavandulaefolia* (newly germinated in the current year, the height of the plants was 8–10 cm), following an “S” shaped path, for sample collection. All samples from the same plot were then combined into a single composite sample. Ultimately, across 5 mining areas and 5 control regions, we obtained a total of 10 rhizosphere soil samples from *B. luminifera* and 10 from *A. lavandulaefolia* (with *n* = 5 for each group). After digging out the whole plant, the bulk soil from its roots were removed via shaking the soil off the root, the residual soil of 1 mm around the root designated as rhizosphere soil, were collected using a sterile brush. Each sample was divided into three parts: one was shipped back to the laboratory using dry ice and stored at −80°C until DNA was extracted for assay microbiological analysis, one was shipped back to the laboratory using ice packs and stored at −4°C for soil microbial biomass, and the remaining samples were shipped to the laboratory in ice packs and air dried for chemical analysis.

### 2.2 Geochemical analysis

To explore the effects of environmental factors on rhizosphere microorganisms, 37 indexes related to soil physical (SMC), chemical properties (pH, SOC, TN, TP, etc.), and metal element (TSb, TAs, ASb, AAs, etc.) were determined (Tables 1, 2). All soil samples sifted through a 2 mm sieve to remove debris (e.g., leaves and roots) and gravel. Soil moisture content (SMC) was analyzed by using fresh soil and determining the mass lost after oven drying at 105°C until a stable weight was reached (30 h). The chloroform fumigation-extraction method was employed to assess the microbial biomass carbon (MBC), nitrogen (MBN), and phosphorus (MBP), and the determination of dissolved organic carbon (DOC) involved the use of fresh soil samples (Xing et al., 2020). The soil was naturally air-dried (< 2 mm) and used for testing other indexes. Soil pH was measured using a pH meter (*g*/*v*, 1:2.5; PHS3C, Leici Instruments, China), cation exchange capacity (CEC) was determined using the NH_4_OAc method, available nitrogen (AN) was assessed by the alkaline hydrolysis distillation method, available phosphorus (AP) was analyzed using the molybdenum blue colorimetry method (Xing et al., 2020), available potassium (AK) was determined through the ammonium acetate leaching flame photometer method, and exchangeable calcium (ACa) and magnesium (AMg) were measured using atomic absorption spectrophotometer (ICE 3500; Thermo Scientific, United States) (Kumar et al., 2018). The available forms of cadmium (ACd), aluminum (AAl), manganese (AMn), nickel (ANi), lead (APb), iron (AFe), copper (ACu), zinc (AZn) in soil were extracted by buffered diethylenetriaminepentaacetic acid (DTPA) (Xing et al., 2023). The available antimony (ASb) and arsenic (AAs) were extracted by 1 M NH_4_H_2_PO_4_ solution (Zhang et al., 2018; Zhong et al., 2020). Part of the soil passed through a 0.149-mm sieve to determine soil organic carbon (SOC) was determined by the K_2_Cr_2_O_7_ method, total contents of nitrogen (TN) was determined by the Kjeldahl determination, phosphorus (TP) was determined by the molybdenum blue colorimetry method, potassium (TK) was determined by the sodium hydroxide melting flame photometer and calcium (TCa) was determined by the titration with the chelating agent EDTA (Kumar et al., 2018; Xing et al., 2020). Total contents of metals including antimony (TSb), arsenic (TAs), cadmium (TCd), aluminum (TAl), manganese (TMn), nickel (TNi), lead (TPb), iron (TFe), copper (TCu) and zinc (TZn) were digested with aqua regia (mixture of hydrochloric acid and nitric acid with volume of 3:1). The contents of metals were determined by an inductively coupled plasma mass spectrometry (ICP-MS 7700, Agilent, United States) (Xing et al., 2023). Meanwhile, the pre-experimental results showed that the form of antimony in the soil of this mining area was mainly Sb (V).

**Table 1:** Physiochemical properties of both plants’ rhizosphere soil in control and mining areas.

	CARS	MARS	CBRS	MBRS
SOC (g·kg^−1^)	22.18 ± 9.20Aa	36.74 ± 15.22Aa	15.74 ± 5.58Aa	22.54 ± 4.06Aa
TN (g·kg^−1^)	1.77 ± 0.67Aa	1.72 ± 0.29Aa	1.22 ± 0.33Aa	1.14 ± 0.33Aa
TP (g·kg^−1^)	0.58 ± 0.32Aa	1.10 ± 0.82Aa	0.49 ± 0.31Aa	1.00 ± 0.82Aa
TK (g·kg^−1^)	4.53 ± 2.25Ab	9.44 ± 3.20Aa	4.39 ± 1.70Ab	8.85 ± 3.59Aa
TCa (g·kg^−1^)	6.57 ± 4.53Ab	21.62 ± 15.54Aa	3.98 ± 3.01Ab	10.74 ± 4.76Aa
CEC (cmol^+^·kg^−1^)	15.62 ± 4.83Aa	10.91 ± 3.63Aa	10.84 ± 1.44Aa	5.27 ± 2.97Aa
DOC (mg·kg^−1^)	96.95 ± 19.68Aa	71.84 ± 25.76Aa	52.67 ± 13.56Ba	56.24 ± 10.29Ba
AN (mg·kg^−1^)	150.20 ± 79.33Aa	105.42 ± 16.15Aa	105.78 ± 44.16Aa	92.10 ± 46.61Aa
AP (mg·kg^−1^)	9.19 ± 4.54Ab	42.36 ± 24.63Aa	6.01 ± 2.48Ab	34.58 ± 23.85Aa
Ak (mg·kg^−1^)	161.12 ± 63.23Aa	257.60 ± 132.97Aa	102.72 ± 21.09Aa	89.34 ± 30.89Ba
ACa (mg·kg^−1^)	3306.95 ± 1659.01Aa	3471.6 ± 3625.81Aa	1514.75 ± 1217.37Aa	1075.60 ± 214.35Aa
AMg (mg·kg^−1^)	165.47 ± 110.12Aa	105.82 ± 61.48Aa	182.79 ± 271.39Aa	43.13 ± 15.36Aa
MBC (mg·kg^−1^)	227.54 ± 77.60Aa	158.77 ± 18.05Aa	175.57 ± 50.37Aa	140.63 ± 19.54Aa
MBN (mg·kg^−1^)	23.29 ± 5.85Aa	14.75 ± 2.73Aa	16.82 ± 4.14Aa	19.52 ± 4.95Aa
MBP (mg·kg^−1^)	8.29 ± 2.32Ab	22.24 ± 5.17Aa	2.81 ± 0.73Bb	7.84 ± 2.00Ba
SMC (%)	22.60 ± 1.74Aa	8.20 ± 3.25Ab	19.60 ± 1.02Ba	7.40 ± 4.03Ab
pH	6.40 ± 0.07Aa	5.14 ± 0.67Ab	6.10 ± 0.14Aa	4.83 ± 0.32Ab

SOC, soil organic carbon; DOC, dissoluble organic carbon; TN, total nitrogen; TP, total phosphorus; TK, total potassium; AP, available phosphorus; AN, available nitrogen; AK, available potassium; TCa, total calcium; ACa, available calcium; CEC, total cation exchange; MBC, soil microbial biomass C; MBN, soil microbial biomass N; MBP, soil microbial biomass phosphorus; SMC, soil moisture content. CARS is *Artemisia lavandulaefolia* rhizosphere in control area, MARS is *Artemisia lavandulaefolia* rhizosphere in mining area, CBRS is *Betula luminifera* rhizosphere in control area, MBRS is *Betula luminifera* rhizosphere in mining area. The results are mean ± SD. Capital letters represent the comparison between two species in the same place. Lowercase letters represent the comparison of the same species between two areas. T-test is used when the data conform to the normal distribution, and Mann–Whitney U test is used when the data do not conform to the normal distribution, *p* < 0.05, similarly hereinafter, *n* = 5.

**Table 2:** Total and available metals of both plants’ rhizosphere soil in control and mining areas.

	CARS	MARS	CBRS	MBRS
TSb (mg·kg^−1^)	76.42 ± 78.84Ab	6711.76 ± 7911.01Aa	51.45 ± 46.57Ab	7555.29 ± 6535.50Aa
TAs (mg·kg^−1^)	90.05 ± 39.94Ab	588.34 ± 434.67Aa	69.71 ± 29.34Ab	463.98 ± 436.03Aa
TCd (mg·kg^−1^)	1.76 ± 1.08Ab	7.18 ± 4.96Aa	1.16 ± 0.47Ab	5.54 ± 4.53Aa
TAl (mg·kg^−1^)	51105.25 ± 6316.04Ab	31091.89 ± 13187.14Aa	38734.10 ± 10023.40Aa	26581.47 ± 17312.60Aa
TMn (mg·kg^−1^)	583.38 ± 173.24Aa	412.17 ± 248.22Aa	514.86 ± 307.95Aa	214.15 ± 202.77Aa
TNi (mg·kg^−1^)	105.63 ± 26.64Aa	39.32 ± 16.23Ab	97.62 ± 22.78Aa	27.93 ± 15.54Ab
TPb (mg·kg^−1^)	15.49 ± 5.52Aa	38.54 ± 24.91Aa	10.22 ± 3.39Aa	38.84 ± 25.26Aa
TFe (mg·kg^−1^)	44929.03 ± 6741.37Aa	52418.93 ± 11560.42Aa	38164.57 ± 11155.33Aa	48948.45 ± 6547.93Aa
TCu (mg·kg^−1^)	156.78 ± 70.14Aa	186.74 ± 132.41Aa	145.93 ± 72.81Aa	202.02 ± 184.52Aa
TZn (mg·kg^−1^)	155.09 ± 33.72Ab	288.95 ± 345.98Aa	140.12 ± 39.60Aa	98.09 ± 28.14Bb
ASb (mg·kg^−1^)	0.68 ± 0.11Ab	52.89 ± 47.33Aa	0.58 ± 0.15Ab	35.17 ± 40.70Aa
AAs (mg·kg^−1^)	6.90 ± 3.98Ab	85.24 ± 105.64Aa	8.86 ± 2.68Ab	46.26 ± 29.56Aa
ACd (mg·kg^−1^)	0.14 ± 0.09Aa	0.04 ± 0.03Aa	0.09 ± 0.08Aa	0.04 ± 0.02Aa
AAl (mg·kg^−1^)	4.53 ± 8.38Aa	5.36 ± 6.79Aa	1.45 ± 0.65Aa	9.02 ± 11.07Aa
AMn (mg·kg^−1^)	14.39 ± 16.31Aa	6.14 ± 3.84Aa	8.56 ± 7.52Aa	5.31 ± 3.12Aa
ANi (mg·kg^−1^)	0.45 ± 0.68Aa	0.59 ± 0.30Aa	0.19 ± 0.17Aa	0.37 ± 0.08Aa
APb (mg·kg^−1^)	1.50 ± 0.85Aa	0.67 ± 0.42Aa	1.02 ± 0.48Aa	1.11 ± 1.30Aa
AFe (mg·kg^−1^)	10.73 ± 8.62Ab	55.31 ± 35.94Aa	8.19 ± 3.86Ab	89.68 ± 43.92Aa
ACu (mg·kg^−1^)	1.47 ± 1.59Ab	10.75 ± 12.32Aa	1.02 ± 0.75Ab	13.01 ± 13.79Aa
AZn (mg·kg^−1^)	2.63 ± 2.43Aa	4.96 ± 5.83Aa	1.62 ± 1.90Aa	1.77 ± 0.98Aa

TSb, total antimony; TAs, total arsenic; TCd, total cadmium; TAl, total aluminum; TMn, total manganese; TNi, total nickel; TPb, total lead; TFe, total iron; TCu, total copper; TZn, total zinc; ASb, available antimony; AAs, available arsenic; ACd, available cadmium; AAl, available aluminum; AMn, available manganese; ANi, available nickel; APb, available lead; AFe, available iron; ACu, available copper; AZn, available zinc. The results are mean ± SD. Capital letters represent the comparison between two species in the same place. Lowercase letters represent the comparison of the same species between two areas. T-test is used when the data conform to the normal distribution, and Mann–Whitney U test is used when the data do not conform to the normal distribution, *p* < 0.05, *n* = 5.

### 2.3 DNA extraction, amplification, sequencing, and bioinformatic analysis

Soil DNA was extracted from 0.5 g of frozen soil samples utilizing the FastDNA SPIN Kit for Soil (MP Biomedicals; Solon, OH, United States) and the FastPrep-24 instrument (MP Biomedicals) in accordance with the manufacturer’s instructions. The quality and concentration of DNA were determined by 1.0% agarose gel electrophoresis and a NanoDrop2000 spectrophotometer (Thermo Scientific, United States) and kept at −80°C prior to further use. The hypervariable region V3-V4 of the bacterial 16S rRNA gene was amplified with primers 338F (5′-ACTCCTACGGGAGGCAGCAG-3′) and 806R (5′-GGACTACHVGGGTWTCTAAT-3′) (Yan et al., 2017), while the ITS1 region of the fungal internal transcribed spacer (ITS) was amplified with primers ITS1F (5′-CTTGGTCATTTAGAGGAAGTAA-3′) and ITS2R (5′-GCTGCGTTCTTCATCGATGC-3′) (Wang et al., 2019). Equimolar amounts of the purified amplicons were pooled and subjected to paired-end sequencing on an Illumina NovaSeq PE300 platform (Illumina, San Diego, United States) according to the standard protocols by Majorbio Bio-Pharm Technology Co. Ltd. (Shanghai, China).

Raw reads were quality-filtered by fastp version 0.19.6 (Chen et al., 2018) and merged by FLASH version 1.2.7 (Magoč and Salzberg, 2011) with the following criteria: (i) the reads were truncated at any site receiving an average quality score of < 20 over a 50 bp sliding window, and the truncated reads shorter than 50 bp were discarded, reads containing ambiguous characters were also discarded; (ii) only overlapping sequences longer than 10 bp were assembled according to their overlapped sequence. The maximum mismatch ratio of overlap region is 0.2. Reads that could not be assembled were discarded; (iii) Samples were distinguished according to the barcode and primers, and the sequence direction was adjusted, exact barcode matching, 2 nucleotide mismatch in primer matching. Low quality and chimeric reads were filtered using QIIME (Quantitative Insights Into Microbial Ecology) to obtain the effective reads, which were used for the subsequent analysis (Caporaso et al., 2010; Bokulich et al., 2013). Then the optimized sequences were clustered into operational taxonomic units (OTUs) using UPARSE 7.1 (version 7.1^1^) with 97% sequence similarity level (Schloss and Westcott, 2011; Edgar, 2013), and the most abundant sequence for each OTU was selected as a representative sequence. The data related to soil bacteria and fungi were cross-referenced with the Silva bacterial database (Release138^2^) and the Unite reference database (Release 8.0^3^). Subsequent analyses were conducted using homogenized (minimum sample size) and flattened data. BugBase software^4^ utilized for microbial phenotype prediction. FUNGuild software (Fungi Functional Guild^5^) is used for functional classification of fungi. These data processing and analyses were performed using Majorbio Cloud Platform^6^ developed by Majorbio Bio-Pharm Technology Co., Ltd. (Shanghai, China) (Ren et al., 2022).

All sequencing data were deposited into the NCBI sequence read archive (SRA) database under accession number PRJNA903843 (16S rRNA) and PRJNA904213 (ITS).

### 2.4 Metabarcoding analysis

Based on the sequencing results of 16S and ITS, one sample was selected from each treatment for metabarcoding analysis (Tickle et al., 2013; Sun et al., 2017). Based on MicroPITA analysis of both plants’ rhizosphere soil in control and mining areas (Supplementary Figures S1A,B), i.e., the samples with the highest diversity of α (Maximum diversity), the most extreme species composition (Most dissimilar), can basically reflect the characteristics of the overall species composition (Most representative), were all selected by the above three methods (Multiple selections). Then, combined with the total antimony content in rhizosphere soil, secondary screening was conducted. Total genomic DNA was fragmented using Covaris M220 (Gene Company Limited, China) to an average size of approximately 400 bp. Paired-end library was constructed using NEXTFLEX Rapid DNA-Seq (Bioo Scientific, Austin, TX, United States). Adapters containing the full complement of sequencing primer hybridization sites were ligated to the blunt-end of fragments. The paired-end sequencing of the libraries was performed on an Illumina NovaSeq (Illumina Inc., San Diego, CA, United States) at Majorbio Bio-Pharm Technology Co., Ltd. (Shanghai, China). The data were analyzed on the free online platform of Majorbio Cloud Platform (see text footnote 6). Briefly, the paired-end Illumina reads were trimmed of adaptors, and low-quality reads (length < 50 bp or with a quality value <20 or having N bases) were removed by fastp (version 0.23.1) (Chen et al., 2018).

Metagenomics data were assembled using MEGAHIT^7^ (version 1.1.2) (Li et al., 2015), which makes use of succinct de Bruijn graphs. Contigs with a length ≥ 300 bp were selected as the final assembling result, and then the contigs were used for further gene prediction and annotation. Open reading frames (ORFs) from each assembled contig were predicted using Prodigal/MetaGene^8^ (Noguchi et al., 2006; Hyatt et al., 2010). The predicted ORFs with a length ≥ 100 bp were retrieved and translated into amino acid sequences using the NCBI translation table. A non-redundant gene catalog was constructed using CD-HIT^9^ (version 4.6.1) with 90% sequence identity and 90% coverage (Fu et al., 2012). Using SOAPaligner software^10^ to align the high-quality reads of each sample with the non-redundant gene set (default parameters: 95% identity) and to obtain the abundance information of genes in the corresponding samples (Li et al., 2009). In this study, gene abundance is expressed as the number of reads aligned to each gene per one thousand bases per million sequenced reads (RPKM) (Lawson et al., 2017). The KEGG annotation was conducted using Diamond^11^ (version 0.8.35) against the Kyoto Encyclopedia of Genes and Genomes database^12^ with an e-value cutoff of 1e^−5^ (Buchfink et al., 2015). By conducting a keyword search for QS genes and accessing the UniprotKB database,^13^ we retrieved QS genes and established a QS gene database. Employing the Diamond software, we performed homologous sequence alignment through BLASTP^14^ (version 2.2.31+), comparing non-redundant gene set sequences with the QS database we constructed. Using the SignalP prediction tool^15^ (version 4.1), and employed neural networks and hidden Markov models for the prediction of secretory proteins. The term “Probiotic Effect” is derived from the Probiotic database provided by the China National Center for Bioinformation (CNCB).^16^ These processes were performed using Majorbio Cloud Platform (see text footnote 6) (Ren et al., 2022).

All shotgun metagenome sequencing data generated in this study have been deposited in the NCBI short read archive database under the accession number PRJNA904564.

### 2.5 Statistical analyses

Most of the statistical analyses were carried out by R software (V4.3.2),^17^ including microbial co-occurrence network analysis, the normalized stochasticity ratio analysis (NST), the neutral community model analysis (NCM), the random forest analysis (RF), correlation analysis, the canonical correlation analysis (CCA), the redundancy analysis (RDA) and the Mantel’s test (Wang et al., 2022). Partial statistical analyses were analyzed on the online platform of Majorbio Cloud Platform (see text footnote 6), including the α- diversity index (Chao index and Shannon index), the principal coordinate analysis (PCoA), microbial diversity composition, linear discriminant analysis effect size (LEfSe), linear discriminant analysis (LDA) and Procrustes analysis. In the co-occurrence network analysis, the nodes of co-occurrence network were classified into four categories based on their intramodule connectivity (Zi) and intermodule connectivity (Pi) values: peripherals (Zi < 2.5, Pi <0.62), connectors (Zi < 2.5, Pi >0.62), module hubs (Zi > 2.5, Pi <0.62) and network hubs (Zi > 2.5, Pi >0.62). Apart from peripherals, the remaining three categories can be considered as keystone taxa in the molecular ecological network (Guimerà and Amaral, 2005; Banerjee et al., 2018; Yuan et al., 2021). Prior to significance testing, all parameters in the manuscript were tested for normal distribution and homogeneity of variance. The One-way ANOVA (conforming to normality distribution and homogeneity of variance) and Kruskal-Wallis H test (Does not conforming to normality distribution and homogeneity of variance) were utilized to assess significant differences among multiple groups of samples. Independent samples t-test (conforming to normality distribution and homogeneity of variance) and Mann–Whitney U test (Does not conforming to normality distribution and homogeneity of variance) were used to analyze the significant differences between the two groups. The Fisher’s exact test was employed to test the significant between differences two samples (two-tailed test, fdr multiple test to correct *p*-value, DP: Newcombe-Wilson). All *p* values were corrected using the FDR multiple test. The specific methods and details of the statistical analysis were detailed in the Supplementary – Materials and Methods. All R-related data analysis codes have been uploaded to *Code availability*.

## 3 Results

### 3.1 Microbial community composition, diversity, and biomarker taxa

**Figure 2:**
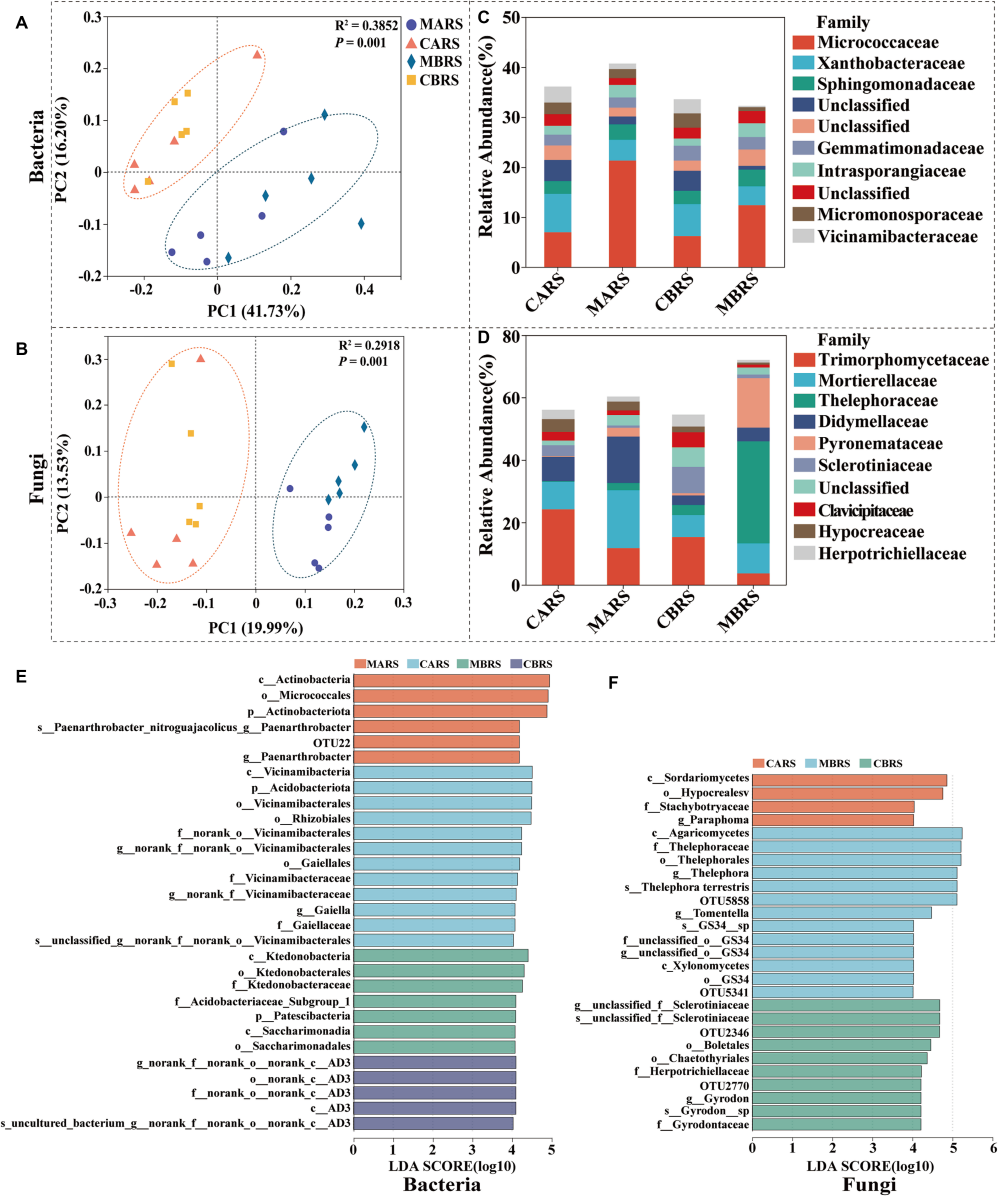
The principal coordinates analysis (PCoA) based on unweighted unifrac distance **(A,B)**, microbial composition **(C,D)** and LEfSe (LDA ≥ 4) results revealed plants rhizosphere microbial biomarkers (from Phylum to OTU level) **(E,F)**. CARS is *Artemisia lavandulaefolia* rhizosphere soil in control area, MARS is *Artemisia lavandulaefolia* rhizosphere soil in mining area, CBRS is *Betula luminifera* rhizosphere soil in control area, MBRS is *Betula luminifera* rhizosphere soil in mining area, similarly hereinafter.

The Chao and Shannon indexes of bacteria and fungi in rhizosphere soil of *B. luminifera* in mining area (MBRS) were significantly lower than those in control area (CBRS) (*p* < 0.05), except the fungal Chao index in MBRS (Supplementary Figures S2A–D). The richness and diversity of microbial community in *A. lavandulaefolia* rhizosphere showed insignificantly difference between the mining area (MARS) and control area (CARS) (*p* > 0.05). There was no significant difference in the Chao and Shannon indexes between *B. luminifera* and *A. lavandulaefolia* in the same area (*p* > 0.05; Supplementary Figures S2A–D). According to PCoA, soil bacterial and fungal communities were clearly separated into mining area and control area (Figures 2A,B). It suggested that the environmental factors (PERMANOVA, *p* = 0.001) had a greater impact on the composition of rhizosphere microbial community compared to the plant species (PERMANOVA, *p* > 0.05) under Sb mining area.

*Micrococcaceae* was more abundant in the rhizosphere of *B. luminifera* and *A. lavandulaefolia* in the mining area, while *Xanthobacteraceae* was more abundant in the rhizosphere of *B. luminifera* in the control area (*p* < 0.05) (Figure 2C). Regarding fungi, in the mining area environment, *A. lavandulaefolia* significantly enriched *Mortierellaceae*, while *B. luminifera* significantly enriched *Thelephoraceae* in the rhizosphere (*p* < 0.05) (Figure 2D). In MARS, one group of bacteria, the *Paenarthrobacter_nitroguajacolicus* (from phylum to species), was significantly enriched, while no fungi were enriched. In MBRS, three groups of bacteria, *Patescibacteria* (the class and its order *Saccharimonadia*), *Ktedonobacteria* (from class to family), *Acidobacteriaceae_Subgroup_1* (within *Acidobacteriota*), and four groups of fungi, *Thelephora_terrestris*, *Thelephora_terrestris*, *Gyrodon*_sp., *GS34*_sp (from phylum to species), and *Xylonomycetes* (the order GS34 and its family unclassified), were significantly enriched (Figures 2E,F). The analysis of dominant microbial phyla, genera and generalists were detailed in the Supplementary – Results Analysis.

### 3.2 Microbial co-occurrence network and assembly process

The empirical network of bacterial associations had more edges, a larger network diameter, a higher mean clustering coefficient, and a higher average path length than the random network, indicating a more complex structure of the empirical network than the random network (Supplementary Table S1). Compared with the control area, the number of edges, graph density, and average clustering coefficient were higher in the rhizosphere microbial co-occurrence network of both plant species in the mining area, and this indicated that the rhizosphere co-occurrence network in the mining area exhibits greater complexity and density. Conversely, the network diameter and average path length are lower in the rhizosphere bacterial co-occurrence network of both plant species in the mining area, while they are longer in the fungal network (Supplementary Table S1), reflecting not only the complexity but also the larger scale of the rhizosphere fungal network in the mining area.

**Figure 3:**
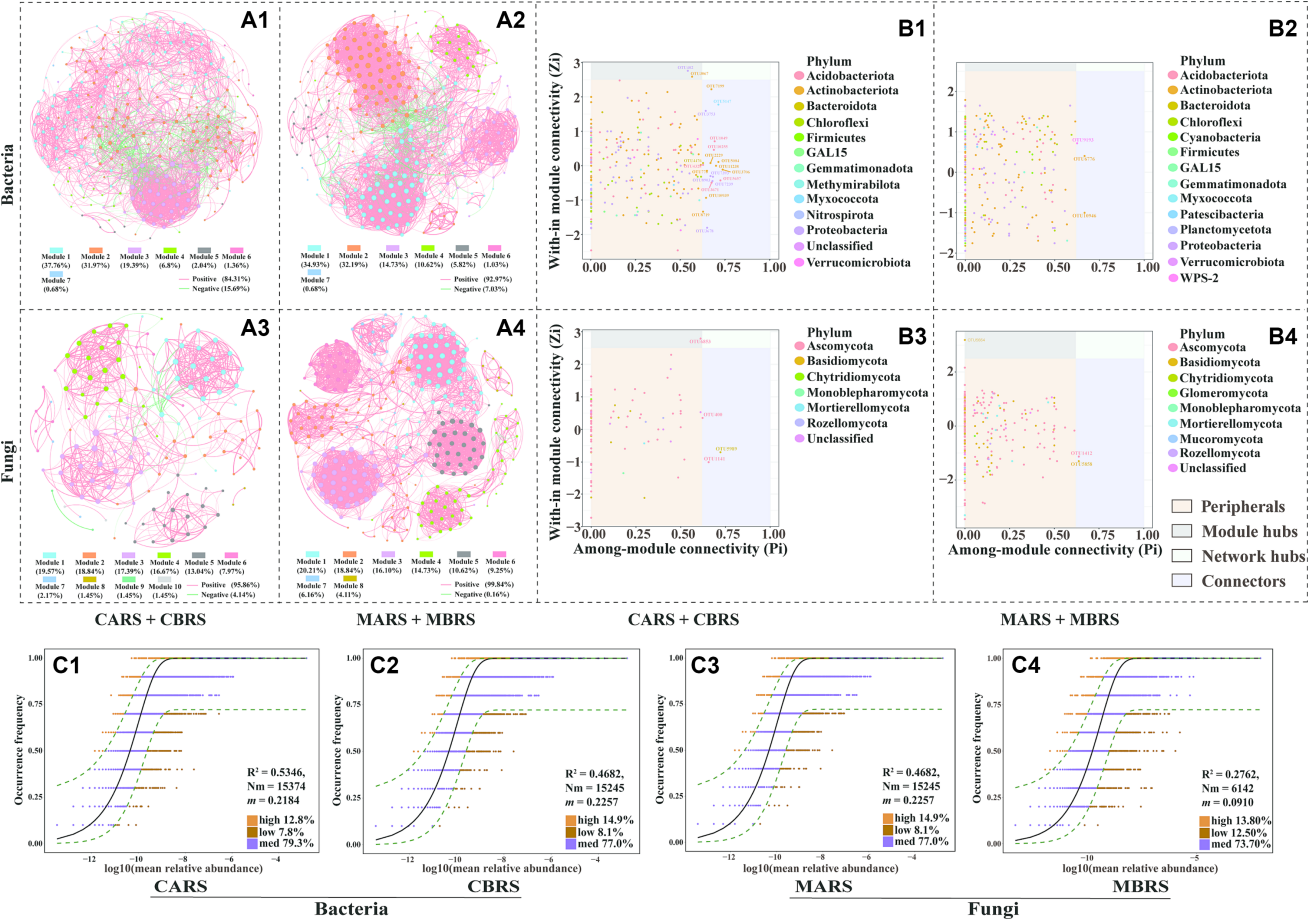
Co-occurrence network of bacterial **(A1,A2)** and fungal **(A3,A4)** communities based on correlation analysis of both plants’ rhizosphere soil in control and mining area. The nodes of co-occurrence network were classified into four categories based on their intramodule connectivity (Zi) and intermodule connectivity (Pi) values: peripherals (Zi < 2.5, Pi < 0.62), connectors (Zi < 2.5, Pi > 0.62), module hubs (Zi > 2.5, Pi < 0.62) and network hubs (Zi > 2.5, Pi > 0.62) **(B1–B4)**. The neutral community model (NCM) was used to analyze the assembly process of microbial communities **(C1–C4)**. The nodes in the network are OTUs (top 300), and they are colored according to modularity class. Red line and green line segments indicate significantly positive and negative correlations, respectively. The connections indicate strong (Pearson’s r > 0.6) and significant correlations (*p* < 0.01), and the size of each node indicates degree **(A1–A4)**. Among them, connectors, module hubs, and network hubs can all be considered as keystone taxa **(B1–B4)**. OTUs that occur more frequently than predicted by the model are shown in orange, while those that occur less frequently than predicted are shown in brown, OTUs that occur within the prediction are shown in and purple. Dashed lines represent 95% confidence intervals around the model prediction (black line) **(C1–C4)**.

In both the control and mining areas’ rhizosphere microbial networks, modules 1, 2, and 3 are the primary modules in the bacterial network, accounting for 89.12 and 81.85% of the total modules, respectively (Figures 3A1,A2). Meanwhile, the primary modules in the fungal network are primarily composed of the top five modules, accounting for 85.51 and 80.5% of the control and mining area rhizosphere networks, respectively (Figures 3A3,A4). Nodes in the main modules of the bacterial network are predominantly attributed to *Actinobacteria* and *Proteobacteria*, while nodes in the main modules of the fungal network are predominantly attributed to *Ascomycota* (Supplementary Figure S5). In the rhizosphere network of the control area, *Microvirga_ossetica* (OTU 402) belonging to *Rhizobiales* and *metagenome_g__Solirubrobacter* (OTU 3067) belonging to *Solirubrobacter* were identified as module hubs (Figure 3B1). Conversely, OTU 9193 (belonging to *WPS-2*), OTU 6776 (belonging to *Acidothermus*), and OTU 10946 (belonging to *Actinobacteriota*) were identified as connectors in the rhizosphere network of the mining area (Figure 3B2). In the rhizosphere fungal network, *Wojnowiciella_leptocarpi* (OTU 6853) belonging to *Phaeosphaeriaceae* and *Udeniomyces_pyricola* (OTU 5664) belonging to *Mrakiaceae* were identified as module hubs in the control area and mining area, respectively (Figures 3B3,B4). Meanwhile, the stability analysis of the microbial co-occurrence network indicated that, whether based on random deletion of network nodes, random species loss, or targeted removal from module hubs, the stability of the rhizosphere microbial network in the mining area is higher than that in the control area (Supplementary Figures S6A–F).

However, the aforementioned results indicate that under the stressful environmental conditions in mining areas, significant changes may have occurred in the community assembly patterns of rhizosphere microorganisms, thereby affecting their diversity, composition, and network co-occurrence patterns. The neutral community model (NCM) explained the variations of 53.46 and 27.37% of the bacterial community, and the variations of 46.82 and 27.62% of the fungal community in mining area and control area, respectively (Figures 3C1–C4). The *m* values (migration rate, 0.0861 for bacteria and 0.0910 for fungi) in mining area was also lower than those in control area (0.2184 for bacteria and 0.2257 for fungi). Meanwhile, the *Nm* values (metacommunity size X migration rate, i.e., *Nm* = *N* X *m*) of mining area (6,061 for bacteria and 6,142 for fungi) were much lower than those of control area (15,374 for bacteria and 15,245 for fungi) in both bacterial and fungal communities (Figures 3C1–C4). The NST*jac* index showed that community assembly of CARS, MARS and MBRS were predominately governed by deterministic processes (NST*jac* < 0.5), while CBRS were primarily controlled by stochastic processes (NST*jac* > 0.5) (Supplementary Figures S7A1,A2). In addition, the relative abundance of stress-tolerant bacteria in the rhizosphere soil of mining area was significantly higher than that in control area (Supplementary Figure S7B1). It’s worth noting that the relative abundance of ectomycorrhizal fungi in the rhizosphere of *B. luminifera* was higher than that of *A. lavandulaefolia*, and the ectomycorrhizal fungi in *B. luminifera* rhizosphere accounted for up to 55.40% in mining area (Supplementary Figures S7B2, S8).

### 3.3 Correlations between microbial communities and geochemical parameters

**Figure 4:**
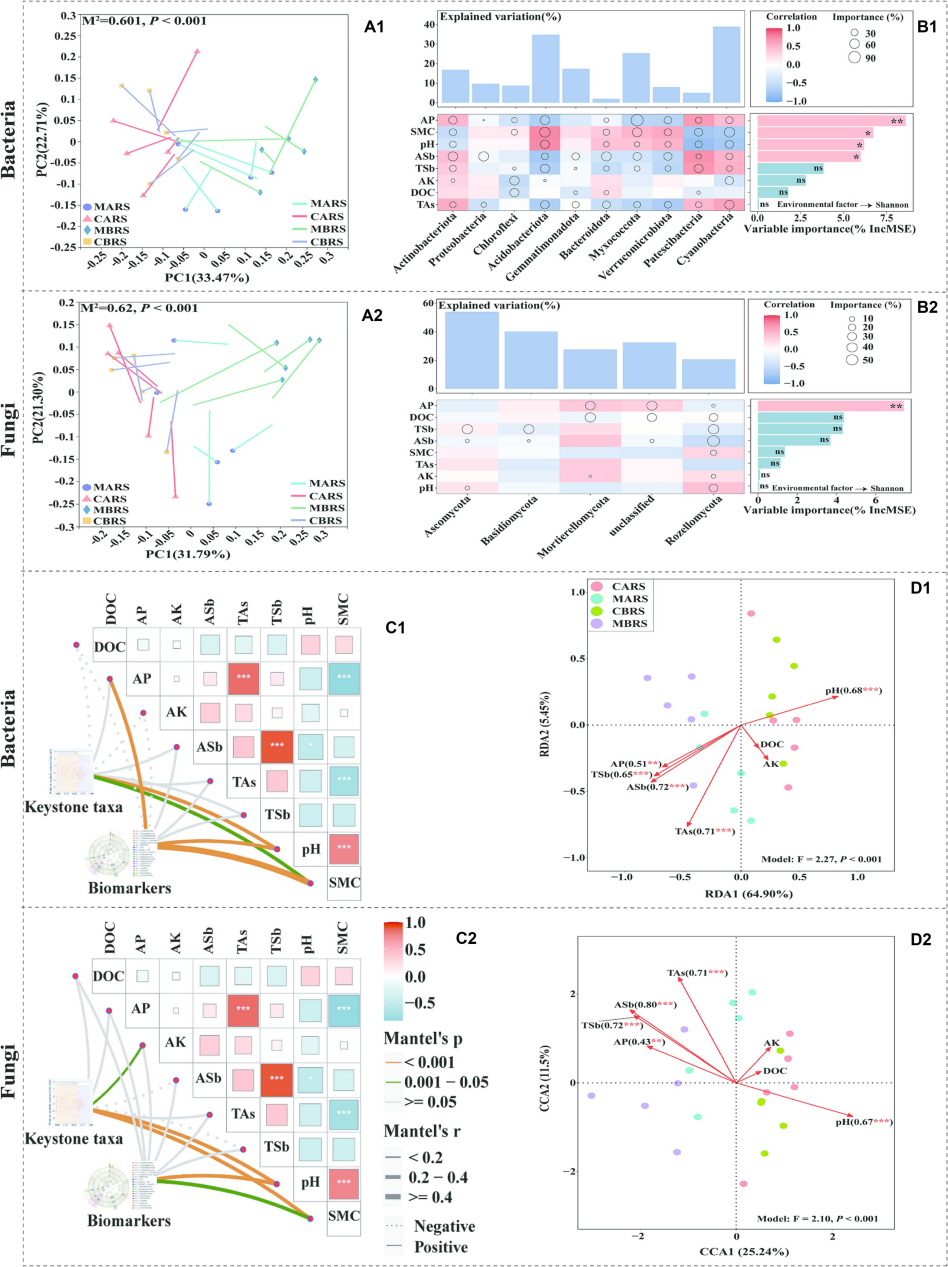
Procrustes analysis between soil microbial OTUs and geochemical parameters based on bray curtis distance **(A1,A2)**. Geochemical parameters associations of the dominant microbial phyla and Shannon index evaluated by correlation and best random forest model **(B1,B2)**. Correlation analysis of keystone taxa, biomarkers and geochemical parameters **(C1,C2)**. Redundancy analysis (RDA) and canonical correlation analysis (CCA) of OTUs and geochemical parameters **(D1,D2)** show the influence of environmental parameters on microorganisms of both plants’ rhizosphere soil in control and mining areas. *M^2^* is the goodness-of-fit statistic of the model, and *P* is the result of Monte Carlo permutation test **(A1,A2)**. Circle size represents the variable’s importance (i.e., the proportion of explanatory variables is calculated through multiple regression modeling and variance decomposition analysis). Colors represent Pearson correlations. The top bar chart shows the total explanatory amount of the corresponding geochemical parameters to each microbial phylum, and the right bar chart shows the importance order of each geochemical parameter to the Shannon index **(B1,B2)**. The edge width corresponds to Mantel’s *r* statistic for the corresponding distance correlations, and edge colour denotes the statistical significance based on 9,999 permutations **(C1,C2)**.

Procrustes analysis revealed a strong correlation between the microbial community composition and rhizosphere environmental factors (goodness of fit *M^2^* = 0.601 and 0.62 for bacteria and fungi, respectively; *p* < 0.001) (Figures 4A1,A2). It showed that available phosphorus (AP), soil moisture content (SMC), pH, available antimony (ASb), total antimony (TSb) and total arsenic (TAs) were the main driving factors for the difference of bacterial phyla (Figure 4B1). However, in addition to *Proteobacteria* and *Gemmatimonadota* were strongly influenced by ASb and TAs, the other phyla were strongly influenced by most environmental factors, including *Acidobacteriota* and *Actinobacteriota* as dominant bacteria. TSb was the main driving factor affecting the difference of fungi (Figure 4B2). The random forest model further determined AP as the primary factor affecting the diversity of bacteria and fungi (Figures 4B1,B2).

The rhizosphere microbial keystone taxa and biomarkers were mainly significantly affected by pH and SMC (Figures 4C1,C2). RDA and CCA analysis further showed that environmental parameters explained 70.35 and 36.74% of the variations of rhizosphere bacterial and fungal community structures, respectively (Figures 4D1,D2). Five environmental variables, i.e., ASb, TAs, pH, TSb and AP, showed the strongest correlation with the changes in rhizosphere bacterial community (R^2^ was 0.72, 0.71, 0.68, 0.65 and 0.51, respectively, *p* < 0.01). The variations of rhizosphere fungal community structure were observed to be closely related to ASb, TSb, TAs, pH and AP (R^2^ was 0.80, 0.72, 0.71, 0.67 and 0.43, respectively, *p* < 0.01). Additionally, this study found that TAl, TMn, TNi, ACd, AAl, ACu, and AFe significantly influenced the composition of rhizosphere bacterial communities (Supplementary Figures S9, S10), while only TZn and ACu significantly affected the composition of rhizosphere fungal communities (Supplementary Figures S11, S12). The geochemical parameters analysis of rhizosphere soil can be found in the Supplementary – Results Analysis.

### 3.4 Functional characteristics of rhizosphere microorganism

**Figure 5:**
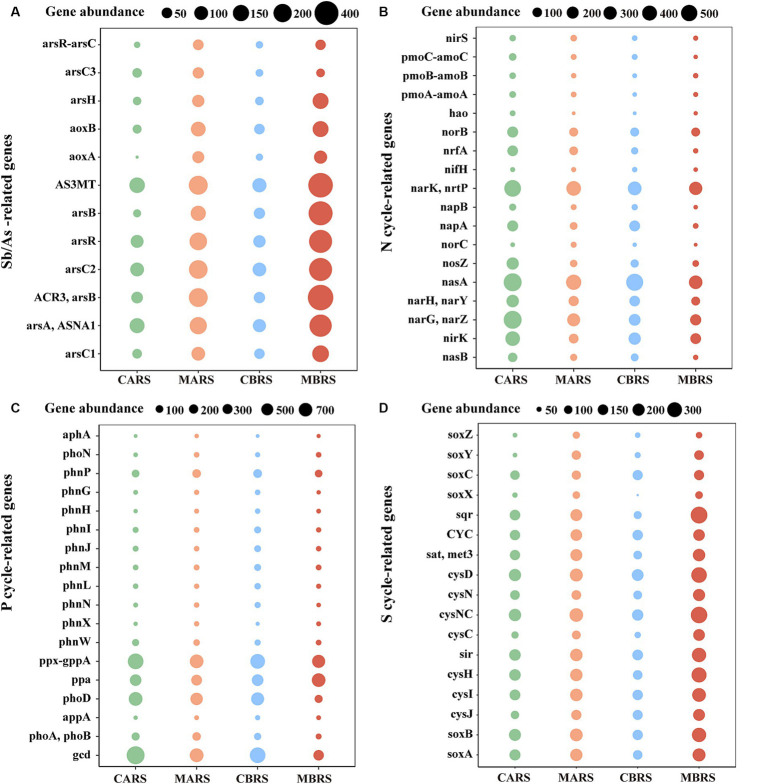
Changes of genes abundance related to Sb/As-resistance **(A)**, nitrogen **(B)**, phosphorus **(C)**, sulfur cycle **(D)** of both plants’ rhizosphere soil in control and mining areas. See Supplementary Figures S14–S17 for statistical difference analysis of genes.

Metabarcoding analysis revealed that, compared to the control area, the rhizosphere of plants in the mining area was enriched with numerous genes related to antimony and arsenic resistance, carbon cycling, nitrogen cycling, phosphorus cycling, and sulfur cycling (Figures 5A–D and Supplementary Figure S13). Specifically, compared to the control area, the rhizosphere of *B. luminifera* in the mining area exhibited significant enrichment of almost all antimony and arsenic resistance genes, such as genes related to As methyltransferase (*AS3MT*), Sb and As-resistance regulation (*arsR*), As transport ATPase (*arsA* and *ASNA1*), Sb and As reductase (*arsC1*, *arsC2*, and *arsC3*), and Sb and As transport (*ACR3*) (Supplementary Figure S14A). Additionally, there was enrichment of Glycosyl Transferases (GT) and Glycoside Hydrolases (GH) genes (Supplementary Figure S14B), genes related to nitrogen cycling (*narK*/*nrtP*, *narG*/*narZ*, and *norB*, etc) (Supplementary Figure S14C), genes related to phosphorus cycling (ppa), and all genes related to sulfur cycling (*cysD*, *sir* and *cysH*, etc) (Supplementary Figure S14D). Similarly, as observed in the rhizosphere of *A. lavandulaefolia* in the control area, the rhizosphere of *A. lavandulaefolia* in the mining area also showed significant enrichment of genes related to Sb and As resistance, C cycling, and S cycling (Supplementary Figure S15). However, compared with *A. lavandulaefolia* in mining area, the abundance of genes related to N cycling and P cycling was significantly lower in the rhizosphere of *B. luminifera* in the mining area (Supplementary Figure S16). Instead, compared with *B. luminifera* in mining area, genes related to N cycling (*nirS* and *norC*) and genes related to P cycling (*phnP* and *phoA*/*phoB*) were significantly enriched in the rhizosphere of *A. lavandulaefolia* in the mining area (Supplementary Figure S17).

This study also revealed that, compared to the control area, a significant enrichment of numerous QS genes and genes related to chemotaxis systems was observed in both MBRS and MARS, especially in MBRS (*p* < 0.05; Supplementary Figures S18A,B–S20). Additionally, the abundance of genes related to the type III secretion system in MARS and MBRS was higher than those in the control area (Supplementary Table S3). Some strains promoting plant growth, such as *Bradyrhizobium japonicum*, *Methylobacterium*, and *Bradyrhizobium pachyrhizi*, were identified and enriched in mining area, and these strains are mainly involved in providing plant nutrition and enhancing plant immunity (Supplementary Figure S21 and Details of growth-promoting strains.xlsx). These strains mainly belong to *Proteobacteria* and *Actinobacteria*, and genes related to Sb and As resistance, C, N, P, S cycling, QS, and chemotaxis systems are primarily provided by *Actinobacteria*, *Proteobacteria*, and *Acidobacteria* (Supplementary Figures S22–S27). The results of environmental correlation showed that these plant probiotics were relatively less affected by environmental factors (Supplementary Figure S28). *Bradyrhizobium* was significantly negatively correlated with AP and TAs, while *Pseudomonas* was significantly positively correlated with AP and TAs (*p* < 0.05). *Methylobacterium* was significantly negatively correlated with pH, *Rhizobium* was significantly positively correlated with available potassium (AK), and *Streptomyces* was significantly positively correlated with TSb.

## 4 Discussion

### 4.1 Structure and assembly of rhizosphere microorganisms in mining areas

It is found that environmental factors showed stronger influence on the microbial community composition than plant species presented in the mining area. The differences of rhizosphere microbial composition and structure between mining areas and control areas depended on the changes of relative abundance of its dominant phyla (Supplementary Figures S4A–D). The specific discussion on α and β diversity of microorganisms can be found in the Supplementary Discussion. The co-occurrence network of rhizosphere microbial communities of both plant species in mining area exhibited greater complexity, with more closely related species (as evidenced by more nodes and edges, a higher average degree, and higher graph density) (Figures 3A1–A4). The higher clustering coefficient and graph density observed in mining area suggested that microbial species in this environment tended to form more tightly connected groups or modules, potentially reflecting adaptation to the harsh and changing conditions in mining area (Meng et al., 2022; Wang et al., 2023). Regardless of whether in the control or the mining environment, the proportion of connections between nodes in microbial co-occurrence network was mainly positively correlated, and this positive correlation was further improved in mining area (Supplementary Table S1). In particular, this study found that increased complexity of rhizosphere microbial networks in mining areas also contributed to network stability. This is because network robustness can be positively correlated with network complexity metrics such as number of nodes and edges, average connectivity, clustering coefficient, modularity, etc. (Yuan et al., 2021; Ling et al., 2022). Furthermore, compared to the control area, the rhizosphere microbial co-occurrence network in the mining area exhibits higher robustness even after the removal of certain microbial groups, including key ones, highlighting the greater ecological niche overlap and functional complementarity of rhizosphere microorganisms in the mining area (Yang et al., 2022; Li et al., 2023). Functional redundancy may play a crucial role in maintaining ecosystem stability, ensuring that functional traits remain more stable in response to environmental changes compared to microbial taxonomic composition (Li et al., 2023). Moreover, the higher functional redundancy of rhizosphere microorganisms also enhances resistance to changes in taxonomic composition caused by biotic or abiotic disturbances (Louca et al., 2018). These findings indicated that microorganisms tend to cooperate relationship in the mining environment, and resulting in a more interconnected (He et al., 2017). Inter-microbial cooperation can promote functional redundancy and resource sharing among community members, enhancing the stability and resilience of microbial communities (Louca et al., 2018). Cooperative interactions can also reduce competition, and enhance resistance to environmental stressors, and allow microbial species to better utilize available resources (He et al., 2017; Gao et al., 2022). Therefore, in mining area, inter-microbial cooperation can lead to greater stability and resilience of the community. Traditional niche-based theories posit that deterministic factors such as species traits, interspecific interactions (e.g., competition, predation, mutualism, and trade-offs), and environmental conditions (e.g., pH, temperature, salinity, and humidity) govern community structure, a process commonly referred to as deterministic assembly (Dini-Andreote et al., 2015; Zhou and Ning, 2017). Conversely, neutral theory assumes that community structure is independent of species traits and is governed by stochastic processes of birth, death, extinction, and speciation (Dini-Andreote et al., 2015; Zhou and Ning, 2017). In this study, we found that rhizosphere microbes of plants in mining areas were subject to significant dispersal limitations compared to those in control areas, indicating that dispersal limitation is a key factor influencing microbial community assembly in mining environments (Figures 3C1–C4). Interestingly, while rhizosphere microbes of *A. lavandulaefolia* were predominantly influenced by deterministic assembly in both environments, those associated with *B. luminifera* showed a shift from stochastic to deterministic assembly in the mining area relative to the control area (Supplementary Figures S6A1,A2). This suggests that rhizosphere microbes of *B. luminifera* in the mining area were subject to strong dual selection pressures from both the plant and the environment (Chen et al., 2019; Ning et al., 2024), and these pressures could have been critical factors contributing to the significant differences observed in the alpha and beta diversity of these microbes (Xun et al., 2019). This finding further supports our previous conclusion that there is a more pronounced selection effect on *B. luminifera* in mining areas.

The study suggested that plants in mining areas selectively enriched specific rhizosphere microorganisms to fulfill their unique requirements, effectively retaining them within the rhizosphere. For instance, *A. lavandulaefolia* and *B. luminifera* exhibited significantly higher abundance of *Mortierellaceae* and *Thelephoraceae* (*p* < 0.05), respectively (Figure 2D). Notably, it’s suggested that *Mortierellaceae* contribute to plant growth enhancement (Escudero-Martinez and Bulgarelli, 2019; Ozimek and Hanaka, 2021; Li et al., 2023) and augment inter-plant competition (Ozimek and Hanaka, 2021; Yang et al., 2022), while also playing a crucial role in soil carbon (Escudero-Martinez and Bulgarelli, 2019; Ozimek and Hanaka, 2021), nitrogen (Li et al., 2023), and phosphorus cycling (Sun et al., 2018). Importantly, it has been shown that *Thelephoraceae*, as ectomycorrhizal fungi, can enhance the tolerance, vigor, and biomass of host plants under heavy metal stress, thereby enhancing the adaptability of plants in mining areas (Yu et al., 2020; Yung et al., 2021; Xiao Y. et al., 2023). The enrichment of these rhizospheric microorganisms might have enhanced the adaptability of *A. lavandulaefolia* and *B. luminifera* to the mining area environment, potentially contributing to their dominance in the region. Interestingly, the evolutionary relationships of the *Micrococcaceae* and *Thelephoraceae* in the rhizosphere of dominant native plants grown in the mining area appear to be relatively independent compared to those in the control area (Supplementary Figures S29A,B). This supports our previous assertion that rhizosphere microorganisms may respond to plant distress signals through rapid evolution under stress conditions.

### 4.2 Environmental factors affecting structure and composition of rhizosphere microbial community

It was suggested that *Actinobacteriota* was more tolerant to heavy metal compared with *Acidobacteriota* (van Bergeijk et al., 2020), which could explain the observed increase of relative abundance of *Actinobacteriota* and decrease of *Acidobacteriota* in mining environments. The increase in relative abundance of *Actinobacteriota* in mining areas may also be an important factor contributing to the increase in relative abundance of stress-tolerant bacteria (van Bergeijk et al., 2020), and this was confirmed by the current study. It is believed that phosphorus is very important to plants and microorganisms (Čapek et al., 2018). The level of AP further increased due to the decrease of pH in mining environment, which became the primary factor affecting the rhizosphere microbial diversity in antimony mining areas, while this was neglected in previous reports. The correlation of environmental parameters and microorganisms were detailed discussed in Supplementary materials.

It implied that pH and SMC were the main factors affected bacterial diversity and co-occurrence networks in mining areas, while the levels of antimony and arsenic were the primary factors affecting the fungal diversity and co-occurrence networks among species (Figures 4C1,C2). Notably, this discovery was different from the general view that microorganisms in antimony mining areas are predominantly influenced by antimony (Sun et al., 2017). The environmental factors, such as pH, organic matter, and other metals, can alter the antimony toxicity, which can further affect the growth and survival of acid-sensitive microorganisms (Zhu et al., 2020). This can lead to cell death or growth inhibition of susceptible microorganisms and result in changes in the microbial community structure and composition (Li et al., 2016; Zhu et al., 2020). Therefore, the RDA results indicated that the primary factor affecting the rhizosphere microbial community structure was found to be ASb (Figures 4D1,D2).

### 4.3 Rhizosphere functional microorganisms may enhance the adaptability of plants to mining stress

Antimony and arsenic are chemically similar homologues, suggesting that microorganisms can transform and transport both elements through similar biological process. Studies have shown that arsenic resistance operon, which includes *arsB*, *arsR* and *arsC*, can be induced by Sb (Sun et al., 2017; Li Y. et al., 2021). Some studies have also shown that Sb may also promote the enrichment of As-related reductase genes (Li C. et al., 2021; Li Y. et al., 2021). Therefore, since arsenic and antimony resistance mechanisms are closely related, analyzing a large number of arsenic oxidation-related genes was still believed to be an effective approach to predict potential patterns of change in antimony oxidation-related genes. It was found that the abundance of Sb/As-related resistance genes in rhizosphere was significantly increased by high Sb content in mining areas, especially for *B. luminifera*, which revealed that *B. luminifera* had stronger potential to resist antimony and arsenic than *A. lavandulaefolia* (Figure 5A and Supplementary Figure S17A). Meanwhile, the genes abundance of six kinds of enzymes involved in carbon cycle of both plant species increased significantly in heavily polluted areas (Supplementary Figures S13, S14B, S15B), which may be an important measure for the rhizosphere-soil interface of plants to resist related stress. It is believed that plant roots can release more carbon-containing compounds into the soil under extreme stress, such as root exudates, which will increase the substrates of enzymatic reaction related to the carbon cycle, thus providing energy for the life of rhizosphere microorganisms (Sanaullah et al., 2012). However, it was found that a large number of high-abundance genes in the rhizosphere of mining area plants were related to the denitrification process (Figure 5B; Supplementary Figures S14C, S15C). Increasing the abundance of denitrification genes in the rhizosphere of plants in mining areas can improve the adaptability of rhizosphere microbial communities, enhance nitrogen cycle and transformation functions, and help plants obtain more nitrogen, which is of great significance to plant growth (Nelson et al., 2016; Kuypers et al., 2018). This also provided a new perspective to understand the interaction between plants and microorganisms in mining areas. Notably, a large number of functional genes related to phosphorus and sulfur cycling were enriched in the rhizosphere of plants in mining areas in the current study, which potentially aids in enhancing the phosphorus supply and detoxification ability of plants in these regions (Figures 5C,D; Supplementary Figures S14D,E, S15D,E), and the specific discussion of functional genes related to N, P and S cycle can be found in Supplementary Discussion.

**Figure 6:**
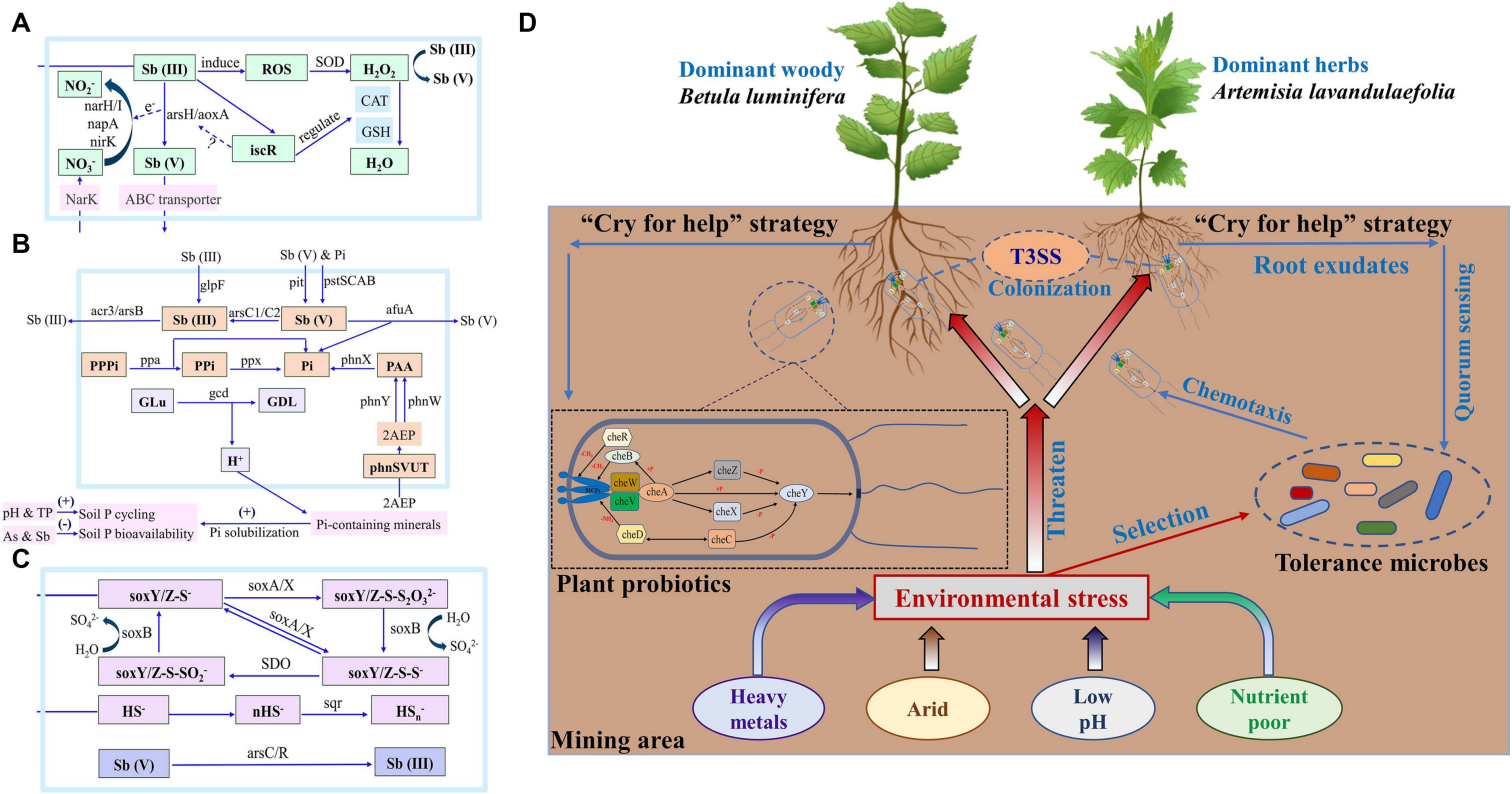
Related genes responsible for N turnover—Sb resistance **(A)**, P turnover—Sb resistance **(B)** and S turnover—Sb resistance **(C)** of both plants’ rhizosphere soil in control and mining areas. Plants in mining areas may undergo co-evolution with rhizosphere microorganisms, thereby enhancing their adaptability **(D)**. Mcps: chemotactic receptor protein.

Nitrogen, phosphorus and antimony being part of the same group on the periodic table and exhibiting similar chemical properties, have led some studies to propose a coupling relationship between genes involved in N, P and S cycling in the microbiome and Sb oxidation (Zhang et al., 2021; Sun et al., 2022; Liu et al., 2023; Xiao S. et al., 2023). It has been observed that nitrate can serve as an electron acceptor during Sb oxidation, enabling the nitrate-dependent Sb oxidation reactions (Zhang et al., 2021; Xiao S. et al., 2023). During the conversion of Sb (III) to Sb (V) by *arsH*/*aoxA* converted, electrons are transferred to NO_3_^−^, which is then reduced to NO_2_^−^ through the action of *narH*/*I*, *napA* and *nirK* (Xiao S. et al., 2023). Furthermore, the microbial phosphate-specific transport protein can utilize the same binding site to absorb both phosphate and antimonate, leading to intracellular Sb accumulation and toxicity (Liu et al., 2023). Consequently, certain Sb resistance genes (e.g., *arsB*, *arsC* and *acr3* etc.) also have the potential to impact phosphorus cycling, allowing bacteria involved in phosphorus cycle to develop various Sb resistance strategies, including oxidation, reduction and efflux (Liu et al., 2023). To mitigate the toxic effects, genes such as *ppa* and *ppx*, involved in oxidative phosphorylation, may cooperate with the arsC, acr3 and arsB to facilitate the efflux process (Li et al., 2016; Liu et al., 2023), thus serving as an important Sb detoxification mechanism for microorganisms. The geochemical conditions observed in Sb mining areas, characterized by low nutrient levels and high S and Sb contents, create a favorable environment for the coupled metabolism of Sb and S (Sun et al., 2022). The *arsC/R* encoded As reductase, while converts Sb (V) to Sb (III), also utilizes sulfide as an electron acceptor and participates in the S oxidation process with the involvement of genes such as *soxA*/*B/X*, representing a key microbial-mediated process in Sb mining areas (Sun et al., 2022). These genes have been found to be significantly enriched in the rhizosphere soil of *B. luminifera* in the mining area, further validating the coupled reaction mechanisms of nitrogen, phosphorus, and sulfur cycling mediated by rhizosphere microorganisms and oxidation–reduction of Sb in Sb-contaminated sites (Figures 6A–C). However, it should be noted that this study found that known non-enzymatic genes associated with antimony and arsenic were lacking in the study area, such as *sodB* and *sodC*. It suggested the microorganisms in the rhizosphere soil obtaining nutrients mainly through root exudates (Čapek et al., 2018), while these exudates may not contain sufficient compounds to induce non-enzymatic antimony oxidation pathways. Meanwhile, the reaction rate of enzymatic antimony oxidation is faster and may adapt more effectively to high antimony pollution levels in rhizosphere soils (Wang et al., 2021).

It is worth noting that, under mining area environment, almost all high-abundance functional genes in the rhizosphere of *B. luminifera* were significantly higher than those in *A. lavandulaefolia*. It indicated that the mining area environment may drive adaptive evolution in *B. luminifera*, leading to changes in the co-occurrence patterns and gene expression profiles of its rhizosphere microorganisms. This may be attributed to the fact that woody plants, characterized by their perennial nature, frequently experience various abiotic stresses such as drought, high temperatures, low temperatures, salinity, and heavy metals throughout their growth (Han et al., 2022). Over the course of prolonged adaptive evolution, woody plants have developed a range of physiological and molecular response mechanisms to cope with adverse environments (Wang et al., 2018). In comparison to herbaceous plants, perennial woody plants are characterized by well-developed root systems, sturdy stems, and rich secondary xylem, which are crucial for enhancing vascular system function and improving stress resistance (Han et al., 2022). The lignification process involves the synthesis of lignin monomers and their subsequent polymerization and deposition into the cell walls, playing a vital role in responding to abiotic stressors (Wang et al., 2018; Han et al., 2022).

To sum up, the increased abundance of functional genes in the rhizosphere of plants in mining areas can largely complement and substitute ecological functions lost due to mining disturbances. This supports the maintenance of normal physiological and metabolic activities of plants and ecosystems, and enhancing the adaptability of native plants in mining areas. In addition, *Proteobacteria*, *Actinobacteria* and *Acidobacteriota* were the main contributors of genes involved in As-resistance and biogeochemical cycles of carbon, nitrogen, phosphorus and sulfur. This further suggested that these phyla played an important role in sustaining ecological processes and recovery from environmental perturbations in mining areas.

### 4.4 Eco-evolutionary responses of rhizosphere microorganisms to environmental stress in mining areas

Plants in mining areas form a symbiotic relationship with rhizosphere microorganisms, adjusting their root secretions to nurture these microbes which in turn aid plant growth and survival, a process known as co-evolution that enhances adaptability to stressful conditions for both (Otto, 2021; Tan et al., 2021; Fields and Friman, 2022; Trivedi et al., 2022). Bacterial chemotaxis senses plant root exudates, showcasing their active adaptability and facilitating the recruitment and assembly of the rhizosphere microbiome (Wadhams and Armitage, 2004; Philippot et al., 2013). This process regulates bacterial colonization levels in roots and their viability in rhizosphere soil, altering soil microbial community structure and initiating symbiotic relationships with plants (Adadevoh et al., 2016; Raina et al., 2019). The rhizosphere of both plant species had higher abundance of genes related to chemotaxis in mining areas indicated the chemotaxis of rhizosphere microorganisms to root exudates might regulate their root colonization to the host (Supplementary Figures S18A, S19). Meantime, the enrichment of chemotactic genes in the rhizosphere soil of mining area suggested the efficient nutrient cycle and energy flow microsystem than those in control area, thus promoting the absorption and utilization of nutrients by plants (Pedler et al., 2014; Adadevoh et al., 2016; Smriga et al., 2016). Many root-related bacteria need QS to colonize the rhizosphere and regulate a variety of phenotypes, including rhizosphere competitiveness, virulence, conjugation, secretion of hydrolase and production of secondary metabolites (Trivedi et al., 2020; Ling et al., 2022). The abundance of QS gene in the rhizosphere of both plant species in mining area was significantly higher than those in control area (Supplementary Figures S18B, S20), which indicated that the more potential conducive to QS signal transmission in the rhizosphere of both plant species in mining area. In this process, bacteria can use the chemotaxis system to sense and respond to signaling molecules and approach the surface of plant roots (Jin et al., 2019). The high abundance of QS genes in the rhizosphere soil (especially *B. luminifera*) indicated a more efficient and closer communication system between rhizosphere bacteria and their hosts in mining areas. Therefore, the high abundance of QS gene deepens the communication between plants and rhizosphere microorganisms, which was helpful for them to form a stable dependence and even co-evolution.

The secretion of protein plays an important role in the information transmission, survival, and growth of bacteria, and it is an important way to ensure the interaction between bacteria and host cells (Costa et al., 2015). The abundance of secreted protein genes was elevated in both plant species in mining area than those in control area. The increased report on plant genetic regulatory factors which can integrate biotic/abiotic stress signals and actively reshape plant microbial communities, further supports the hypothesis that plants “cry for help” to microorganisms (Carrión et al., 2019; Li H. et al., 2021). This study found that, in response to extreme environmental stress, plants in mining areas enrich a large number of functional microorganisms through similar strategies, potentially enhancing their adaptability (Figure 6D). It was worth mentioning that, the genes abundance of type III secretion system (T3SS) increased in the rhizosphere of both plant species in mining area than those in the control area (Supplementary Table S3). The T3SS system, is believed to serve solely as a channel for pathogenic bacteria to invade plants, is also utilized by many rhizosphere growth-promoting bacteria (PGPR) to establish mutually beneficial relationships by circumventing plant immune responses (Yu et al., 2019). Rhizobia’s T3SS effectors, known as nodulation outer proteins (Nops), regulate nodulation or test host specificity, demonstrating how beneficial root microflora manipulate plant immune signals (Miwa and Okazaki, 2017). This suggests that microbes in mining areas actively seek plant assistance due to environmental stress, prompting plant responses and indicating co-evolution between plants and microorganisms in natural communities, akin to the binary interaction model of plants and pathogens (Delaux and Schornack, 2021). The co-evolution between plants in mining areas and *B. luminifera* may have driven the genetic differentiation of *B. luminifera* in mining areas (Supplementary Figure S30). This also partially confirms our second hypothesis, which is the possible co-evolution between plants and microorganisms in the mining area, which may have enhanced the long-term adaptability of the plants in that environment.

In short, the rhizosphere microbes of *B. luminifera* and *A. lavandulaefolia* showed similar strategies when being faced extreme environmental pressures, thus helping their host plants to better adapt to the environment. It is further speculated that the microbial community in the early ecological stage of mining area may be weak, while plants can enrich more functional microorganisms without changing the original microbial community greatly, thus making the rhizosphere microbial community beneficial to themselves stronger.

## 5 Conclusion

Here, the rhizosphere microorganisms of two native dominant plants (*Artemisia lavandulaefolia* and *Betula luminifera*) in Sb mining area in Southwest China were selected and studied. The soil exhibited high Sb level, imbalanced nutrient, lower pH and reduced water content, which resulted in changes in the flora structure alterations in the composition and diversity of rhizosphere microbial communities. In response to these environmental pressures, the dominant plants significantly enriched *Proteobacteria* and *Actinobacteria* in the mining area. These microbial populations harbored a large number of genes associated with Sb and As resistance, as well as genes involved in carbon, nitrogen, phosphorus and sulfur cycling. Through the concerted action of Sb/As resistance genes and N, P, S cycling-related genes, the detoxification capacity of rhizosphere microorganisms might have been enhanced, potentially enhancing the resistance and adaptability of the plants in the mining area. Despite the notable differences between herbaceous (*A. lavandulaefolia*) and woody vegetation (*B. luminifera*), their rhizosphere microbes exhibited similar strategies, such as disrupting microbial quorum sensing and chemotaxis, to enrich abundant functional microorganisms in the rhizosphere. These strategies assist the host plants in better adapting to the challenging environment while improving their overall living conditions.

## Data availability statement

The original contributions presented in the study are included in the article/Supplementary material, further inquiries can be directed to the corresponding author.

## Author contributions

WX: Data curation, Formal analysis, Investigation, Methodology, Software, Validation, Visualization, Writing – original draft, Writing – review & editing. XG: Conceptualization, Supervision, Writing – review & editing. LX: Validation, Writing – review & editing. SL: Writing – review & editing. XZ: Writing – review & editing. FJ: Writing – review & editing. GC: Conceptualization, Funding acquisition, Project administration, Resources, Supervision, Validation, Writing – review & editing.

## Footnotes

^1^ http://drive5.com/uparse/

^2^ http://www.arb-silva.de

^3^ http://unite.ut.ee/index.php

^4^ https://bugbase.cs.umn.edu/index.html

^5^ http://www.funguild.org/

^6^ http://www.majorbio.com

^7^ https://github.com/voutcn/megahit

^8^ http://metagene.cb.k.u-tokyo.ac.jp/

^9^ http://www.bioinformatics.org/cd-hit/

^10^ http://soap.genomics.org.cn/

^11^ http://www.diamondsearch.org/index.php

^12^ http://www.genome.jp/keeg/

^13^ http://www.uniprot.org/

^14^ http://blast.ncbi.nlm.nih.gov/Blast.cgi

^15^ http://www.cbs.dtu.dk/services/SignalP/

^16^ http://bidd2.nus.edu.sg/probio/homepage.htm

^17^ http://www.r-project.org
