# Supplementary material for: Enriched rhizospheric functional microbiome may enhance adaptability of Artemisia lavandulaefolia and Betula luminifera in antimony mining areas
Source: Front Microbiol. 2024 Mar 21;15:1348054. doi: 10.3389/fmicb.2024.1348054 (PMC10993014; doi:10.3389/fmicb.2024.1348054)
Supplement: Supplementary file 1 [file Data_Sheet_1.docx]

■ **Supplementary-Materials and Methods**

**Study sites**. The research area is located in Dachang Town (25°33′ - 26°40′ N, 105°05′ - 105°25′ E), Qinglong County, Guizhou Province. The research area belongs to a subtropical humid climate zone, with an annual average temperature of 14.0-14.7 ℃ and abundant rainfall of about 1590 mm, which is mostly concentrated in May-October, accounting for more than 80% of the annual rainfall. The frost-free period of this area is 250-320 d days, with abundant heat, and the average annual sunshine of about 1,454-1,714 hours. The surface of the survey area is arid and short of water, and the terrain is complicated. There is without human interference in the tailings (slag) accumulation area (with a total area of about 10 hm^2^) after antimony mining. The trees in this area are only *Betula luminifera* (about 8-15 years old), and the herbaceous plants are mainly *Artemisia lavandulaefolia*, etc (Du et al., 2023).

**Statistical Analyses.** Uparse (V7.0.1090 http://drive5.com/uparse/) was used to statistically analyze the biological information of ASV, and Mothur (V5.1.30.2 https://mothur.org/wiki/calculators/) was used to analyze the Chao index and Shannon index of bacteria and fungi communities. The Venn diagram (venn diagram function in ‘Venn Diagram’ software package) was used to statistically analyze the number of common and unique ASVs in the sample. QIIME 2 was used to calculate beta diversity distance matrix, and principal coordinate analysis (PCoA) based on unweighted unifrac distance was used to compare microbial groups. Permutal multivariate analysis of variance (PERMANOVA) was used to determine the similarity of microbial groups ASVs among samples. Linear discriminant analysis effect size (LEfSe) was applied to identify significantly different bacterial species (biomarkers) among different reclamation stages based on the Kruskal Wallis ranked sum test (*P* <0.05), and threshold of the linear discriminant analysis (LDA) is 4. Through PERMANOVA analysis, it was found that there was no significant difference between groups after clustering the both plants respectively (CARS&CBRS, R = 0.1000, *P* = 0.1690; MARS&MBRS, R = 0.1760, *P* = 0.1170), but after clustering according to the mining area and the control area, we found that the difference between groups were significantly greater than that within groups (CARS&MARS, R = 0.3920, *P* = 0.0280; CBR & MBRS, r = 0.7120, *P* = 0.0090), that is, it is more meaningful to group according to these two factors. Therefore, we chose the grouping of soil types instead of plants to explore the interaction mode of microorganisms was used by microbial co-occurrence network analysis. The normalized stochasticity ratio (NST) was applied to quantitively evaluate the relative role of stochastic (NST > 50 %) versus deterministic (NST < 50 %) processes in microbial community assembly, NST analysis was performed with the NST package in R (v.4.0.3) (Xing et al., 2023).

First, we use Procrustes analysis based on Bray Curtis distance and PCoA ranking to analyze the goodness of fit and correlation between two rankings of all environmental factors and species data, and use Monte Carlo replacement test to test its significance. Then, the environmental factors were screened for many times by using *VIF* variance expansion factor analysis, and all the environmental factors with *VIF* > 10 were filtered out until all the *VIF* values corresponding to the screened environmental factors were less than 10, and the subsequent environment-species correlation analysis was carried out by using the screened environmental factors (Table S2). Mantel tests, redundancy analysis (RDA) and multivariate direct gradient analysis (CCA) are used to reflect the relationship between flora and environmental factors (firstly, DCA analysis is made with species-sample data, CCA analysis is used if the first axis of Lengths of gradient is greater than or equal to 3.5, and RDA analysis is used if it is less than 3.5), Hmisc (*V.* 4.8-0), dplyr (*V.* 1.0.7) and linkET (*V.* 0.0.7.1) packages were usd for Mantel tests, and ‘Vegan’ package was used for RDA and CCA analysis and mapping. Based on the results of PCoA analysis, the relationship between environment and species was evaluated. In order to determine the most important environmental factors affecting microbial diversity, we use the default parameters of R program to conduct random Forest analysis on Shannon index of microorganisms and environmental factors (R package "random forest", ntree = 1,000, and the default mtry is p/3, where p is the number of this group). Quorum sensing (QS) analysis uses Diamond software, through BLASTP (http://blast.ncbi.nlm.nih.gov/Blast.cgi, *V* 2.2.31+), compares the sequences of non-redundant gene sets with the homologous sequences of our constructed QS database, and gets the annotation results (the expected value of comparison parameters is set to E-value ≤ 1E-5), so as to analyze the types and abundance of QS genes in metagenome samples. Signal peptide prediction tool Signal p (http://www.cbs.dtu.dk/services/SignalP/, *V*. 4.1) was used to predict secreted protein by neural network and hidden Markov model. The software EffectiveT3 is used to predict the input amino acid sequences, and each amino acid sequence is scored by its internal specific calculation model, and the sequence whose score is higher than the threshold is selected, which is the type III secretory system effector protein. In addition, we selected 52 strains beneficial to plants from Probio's probiotic database (http://bidd.group/probio/homepage.htm).

**References**

Du, Z.Y., Xing, W.L., Xue, L., Xiao, J., Chen, G.C., 2023. Niche characteristics and interspecific association of main plant species in antimony mining sites of karst rocky desertification area, Guizhou, China. Acta Ecologica Sinica. 43 (7), 2865-2880. (In Chinese)

Xing, W., Gai, X., Ju, F., Chen, G., 2023. Microbial communities in tree root-compartment niches under Cd and Zn pollution: structure, assembly process and co-occurrence relationship. Sci. Total Environ. 860, 160273.
