# Supplementary material for: Enriched rhizospheric functional microbiome may enhance adaptability of Artemisia lavandulaefolia and Betula luminifera in antimony mining areas
Source: Front Microbiol. 2024 Mar 21;15:1348054. doi: 10.3389/fmicb.2024.1348054 (PMC10993014; doi:10.3389/fmicb.2024.1348054)
Supplement: Supplementary file 2 [file Data_Sheet_2.docx]

■ **Supplementary - Results Analysis**

**Microbial community composition.** The dominant bacterial phyla were *Actinobacteriota*, *Proteobacteria*, *Chloroflexi*, *Acidobacteriota* and *Gemmatimonadota*, which accounted for more than 85% of the total relative abundance. *Actinobacteriota* was significantly more abundant in the rhizosphere of *A. lavandulaefolia* and *B. luminifera* in the mining area, while *Acidobacteria* was more abundant in the rhizosphere soil of the control area (*P* < 0.05) (Fig. S3a). Among these, the *Arthrobacter* (belonging to the *Actinobacteria*), exhibited significant enrichment in the rhizosphere of the mining area, especially in the MARS, while *Sphingomonas* showed relatively higher abundance in the MBRS (Fig. S3b). The most abundant fungal phyla were *Ascomycota*, *Basidiomycota* and *Mortierellomycota*, which accounts for more than 97% of total relative abundance (Fig. S3 c). At the genus level, compared to the control area, in the mining area environment, the rhizosphere of *A. lavandulaefolia* significantly enriched *Mortierellaceae* (belonging to the *Mortierellomycota*), while the rhizosphere of *B. luminifera* significantly enriched *Thelephoraceae* (belonging to the *Basidiomycota*) (Fig. S3 d). There were no specific taxa (i.e., Microbial species were similar) in the rhizosphere microorganisms of both plants in mining area, which indicated the microbial species of two plants are highly overlapped in mining area and control area at the phylum level, and these shared species (generalists) belong to the dominant species (Fig. S4 a and b).

**Correlations between microbial communities and** **geochemical parameters.** In order to reflect the collected soil conditions more comprehensively and truly, we have determined 37 geochemical parameters, including 17 common soil basic physical and chemical properties (Table.1) and 20 metal element parameters (Table. 2). After tested and analysed the significant differences between groups, we found that there were significant differences in 18 geochemical parameters. Geochemical analysis showed that both plants’ rhizosphere soil samples from the mine area contained a significantly higher concentration of TK, TCa, AP, MBP, TCd, TAl, TSb, TAs, AFe, ACu, ASb and AAs than control area, while the significantly higher concentrations of SMC, pH and TNi in the control area rhizosphere soil than the mine area (*P* < 0.05). The content of TZn in rhizosphere soil of MARS is significantly higher than CARS, while MBRS is significantly lower than CBRS (*P* < 0.05). Except for TZn, we found that there was no significant difference of the contents of metal elements in rhizosphere soil between *A.lavandulaefolia* and *B.luminifera* in the same sampling sites (*P* > 0.05). The DOC content in rhizosphere soil of *B.luminifera* was significantly lower than that of *A.lavandulaefolia*, while only the AK content in rhizosphere soil of MBRS was significantly lower than that of MARS (*P* < 0.05).

**Functional characteristics of rhizosphere microorganism.** Meanwhile, this study also observed that in the CARS, the genes *AS3MT*, *arsC3*, *arsA*, and *ASNA1* associated with Sb and As - resistance were significantly higher compared to CBRS, while the genes *arsB*, *arsC1*, *aoxA*, and *aoxB* were significantly lower than CBRS (*P* < 0.05) (Fig. S16a). Genes associated with Glycoside Hydrolases (GH) and Carbohydrate Esterases (CE) were significantly enriched in CBRS, whereas genes related to Carbohydrate-Binding Modules (CBM) and Polysaccharide Lyases (PL) were significantly enriched in CARS (*P* < 0.01) (Fig. S16b). Almost all genes associated with nitrogen cycling, the *ppx-gppA* and *phoD* genes related to phosphorus cycling, and the *cysD* and *sat/met3* genes related to sulfur cycling were significantly enriched in CARS (Fig. S16 c, d, and e). However, in the mining area environment, except for genes related to nitrogen and phosphorus cycling, most genes associated with Sb, As resistance, carbon cycling, and sulfur cycling were significantly enriched in MBRS (Fig. S17 a, b, c, d, and e).

**Rhizosphere microorganisms in the mining area may have promoted the genetic differentiation of plants in the mining area.** To further corroborate the possibility of co-evolution in *Betula luminifera* grown in the mining area, seeds of *B. luminifera* from both the control and mining areas were collected and sown in contaminated soil from the mining area. After 100 days, leaves of *B. luminifera* were collected for chloroplast whole-genome resequencing to detect mutations (Fig. S30 a and b). Initially, the NC_068733.1 (*B. luminifera* chloroplast genome) was used as the reference sequence for mutation detection. Subsequently, the vcf file was converted into a consensus sequence, and a phylogenetic tree was constructed. Overall, a total of 43 mutation sites were detected. Among these, the proportion of heterozygous sites in the chloroplast genome of *B. luminifera* from the control area was 4.7% (2/43), while the proportion of heterozygous sites in the chloroplast genome of *B. luminifera* from the mining area was 100% (43/43) (Table. S4). This suggests that *B. luminifera* living in the mining area may have already undergone genetic differentiation. The results of the chloroplast genome phylogenetic tree validate our hypothesis. *Betula luminifera*_HAP1 from the mining area and *B. luminifera* from the control area cluster together, while Betula luminifera_HAP2 from the mining area clusters with *Betula hainanensis* (Fig. S30 c). This further illustrates that *B. luminifera* in the mining area may have acquired some heritable advantageous traits during long-term adaptation to stressful environments, allowing the population to evolve. Our earlier research findings also support similar viewpoints.
