# Supplementary material for: Enriched rhizospheric functional microbiome may enhance adaptability of Artemisia lavandulaefolia and Betula luminifera in antimony mining areas
Source: Front Microbiol. 2024 Mar 21;15:1348054. doi: 10.3389/fmicb.2024.1348054 (PMC10993014; doi:10.3389/fmicb.2024.1348054)
Supplement: Supplementary file 3 [file Data_Sheet_3.docx]

■ **Supplementary Discussion**

**Structure and assembly of rhizosphere microorganisms in mining areas**. Generally, heavy metals can change the composition of the microbial community, particularly among microorganisms that thrive in the soil, while plant genotypes also play a role in shaping rhizosphere microbial communities to some extent (Li et al., 2021; Wang et al., 2022). Surprisingly, this study found no significant difference in the α-diversity of rhizosphere microorganisms between *B. luminifera* and *A. lavandulaefolia* living in the same area (*P* > 0.05). This could be attributed to these two plants growing in similar soil environments, where their rhizosphere microorganisms face comparable soil influences such as similar soil pH, moisture content, organic matter and heavy metal content, etc., thus resulting in consistent trends in rhizosphere microbial diversity for both plant types (Philippot et al., 2023). Additionally, as indigenous dominant plants, these two species may have established similar symbiotic relationships with soil microorganisms, leading to comparable microbial diversity in their rhizosphere communities (Yang et al., 2023). In this study, compared with the control area, Sb mining significantly decreased the α -diversity of *B. luminifera* (Fig. S2 a-d). This may be attributed to the ecological niche pressure on certain sensitive microorganisms in the plant rhizosphere under heavy metal stress, leading to the disappearance of some of the more sensitive microorganisms (Chen et al., 2020), and this results in a reduction in the microbial diversity within the rhizosphere of *B. luminifera*. However, this study found that the alpha diversity index of *A. lavandulaefolia* in the antimony mining area did not change significantly. Zhu et al. (2023) have shown that *A. lavandulaefolia*, as a pioneer plant, has a strong rhizosphere effect on the composition and structure of soil total organic carbon (SOC) and dissolved organic carbon (DOC) (Zhu et al., 2023). It can also improve the physicochemical properties of alumina refinery residue areas through strong rhizosphere priming effects, thus stabilizing microbial community structure (Liu et al., 2023a). It is consistent with the above research results, the results of this study indicated that *A. lavandulaefolia* in both mining area and control area have higher SOC and DOC content in the soil compared to *B. luminifera*. Meanwhile, the total nitrogen, total phosphorus, available nitrogen and available phosphorus contents in the *A. lavandulaefolia* rhizosphere soil are also higher than those of *B. luminifera*. This further demonstrates that *A. lavandulaefolia* somewhat improved the rhizosphere soil environment in the mining area, maintained microbial activity and diversity, and thus resulted in the rhizosphere microbial α-diversity being less affected by the environment.

Furthermore, the observed differences in the influence of hosts on microorganisms were noticeably diminished, and highlighted the detrimental effects of extreme heavy metal pollution on the microorganisms (Fig. 2 a and b). This may be due to the fact that the harsh conditions of the mining environment exert strong selective pressure on microorganisms, which leads to the establishment of a microbial community that is better adapted to the prevailing environmental conditions (Li et al., 2021; Xing et al., 2023).

**Environmental factors affecting structure and composition of rhizosphere microbial community.** Antimony can affect the microbial community indirectly by altering the physicochemical properties of the environment. For example, antimony can increase the acidity of soil or water, leading to changes in nutrient availability and pH-dependent microbial growth (Zhang et al., 2022). As the dominant phylum, keystone species and biomarkers, the relative abundance of *Actinobacteriota*, *Acidobacteriota* and *Ascomycota* was influenced by environmental factors to varying degrees. Although *Acidobacteriota* as an acid-tolerant bacteria, can thrive in acidic environments, their relative abundance is decreased in mining environments with high levels of TAs, TSb, and AP, low SMC and low pH (Fig. 4b1). The relative abundance of *Actinobacteria* and *Proteobacteria* was primarily influenced by the ASb, while *Ascomycota* and *Basidiomycota* were influenced by TSb levels. In the rhizosphere of mining environments, the relative abundance of *Mortierellomycota* was influenced by AP and DOC (Fig. 4b2). However, the environmental correlation analysis revealed that plant probiotics were relatively insensitive to environmental factors (Fig. S28). This further verified that the rhizosphere probiotics recruited by the plant have relatively robust tolerance to environmental stressors.

**Rhizosphere functional microorganisms enhance the adaptability of plants to the environment of antimony mining area.** Microorganisms involved in nitrogen cycling played an active role in metal contaminated soil, and might provide available nitrogen for the ecosystem and play an active role in bioremediation (Montiel-Rozas et al., 2016). However, it was reported that the nitrate concentration in contaminated sites was significantly lower (Sun et al., 2020; Xu et al., 2020). It was found that a large number of high-abundance genes in the rhizosphere of mining area plants were related to the denitrification process (Fig. 5b; Fig. 14c; Fig. 15c), such as the main function of *nasA*, *nasA*/*B* and *narG* is to convert NO_3_^-^ into NO_2_^-^, and the main function of nir*S*/*K* is to convert NO_2_^-^ into NO, the main function of nor*B* is to convert NO into N_2_O, while the main function of nosZ is to convert N_2_O into N_2_ (Sun et al., 2020; Li et al., 2021). The significant increase in genes related to denitrification process may be the main reason for the decrease in AN content in the rhizosphere soil of plants in mining areas (Fig. 14c; Fig. 15c). Moreover, phosphate solubilizing microorganisms can also promote the dissolution of insoluble mineral phosphorus (including calcium phosphate and apatite), and can chelate with cations such as Fe^3+^, Ca^2+^, Mg^2+^ and Al^3+^, that was releasing phosphate ions and increasing available phosphorus in the soil (Kishore et al., 2015). This may be the reason why the contents of the AP and MBP in rhizosphere soils of the mining area increased significantly.

The acidic soil environment in mining area was conducive to the transformation of insoluble inorganic phosphorus into soluble orthophosphate (Liu et al., 2023b). It was observed 9 genes (*appA*, *phnH* and *phnM*, et al.) involved in mineralization of organic phosphorus and 2 genes (*ppa* and p*px-gppA*) involved in dissolution of inorganic phosphorus were significantly enriched in both plants’ rhizosphere soils in mining area (Fig. 5c; Fig. 14d; Fig. 15d). This elucidated that indigenous microorganisms in mining soils adapt to their harsh habitat in mining environment by boosting phosphorus uptake and metabolic capacities. Such adaptation helps conserve phosphorus equilibrium within the ecosystem and furnishes plants with beneficial rhizospheric services. Sulfur cycle plays an important role in metal mobilization or immobilization via sulfur oxidation or sulfate reduction (Anderson and Lovley 2000). The sulfur oxidation and sulfate reduction genes in all macro-genome samples were detected in the current study, which indicated that innate microbiome in the rhizosphere of both plant species in mining area had the potential to drive the complete sulfur cycle. Among the 17 functional genes related to sulfur cycle, 15 genes were enriched in the rhizosphere of both plant species in mining area (Fig. 5d; Fig. 14e; Fig. 15e). Sulfite reductase genes such as cysJ, cysI and sir may be resistant to heavy metals such as Sb and As, which is of great environmental significance due to the precipitation to metals reducing mobility and toxicity via sulfate reduction (Gao et al., 2021).

**References**

Anderson, R.T., Lovley, D.R. (2000). Anaerobic bioremediation of benzene under sulfate-reducing conditions in a petroleum-contaminated aquifer. Environ. Sci. Technol. 34 (11), 2261-2266.

Gao, L., Li, R., Liang, Z., Wu, Q., Yang, Z., Li, M., Chen, J., Hou, L., 2021. Mobilization mechanisms and toxicity risk of sediment trace metals (Cu, Zn, Ni, and Pb) based on diffusive gradients in thin films: a case study in the Xizhi river basin, South China. J. Hazard. Mater. 410, 124590.

Kishore, N., Pindi, P.K., Ram Reddy, S., 2015. Phosphate-solubilizing microorganisms: a critical review. In *plant biology and biotechnology: Volume I: Plant Diversity, Organization, Function and Improvement*, Bahadur, B., Venkat Rajam, M., Sahijram, L., Krishnamurthy, K.V., Eds. Springer India: New Delhi. pp 307-333.

Li, Y., Zhang, M., Xu, R., Lin, H., Sun, X., Xu, F., Gao, P., Kong, T., Xiao, E., Yang, N., Sun, W., 2021. Arsenic and antimony co-contamination influences on soil microbial community composition and functions: Relevance to arsenic resistance and carbon, nitrogen, and sulfur cycling. Environ Int. 153, 106522.

Liu, C., Jiang, M., Yuan, M.M., Wang, E., Bai, Y., Crowther, T.W., Zhou, J., Ma, Z., Zhang, L., Wang, Y., Ding, J., Liu, W., Sun, B., Shen, R., Zhang, J., Liang, Y., 2023a. Root microbiota confers rice resistance to aluminium toxicity and phosphorus deficiency in acidic soils. Nat. Food. 1-13.

Liu, S., Zeng, J., Yu, H., Wang, C., Yang, Y., Wang, J., He, Z., Yan, Q., 2023b. Antimony efflux underpins phosphorus cycling and resistance of phosphate-solubilizing bacteria in mining soils. ISME J. 17, 1278-1289.

Montiel-Rozas, M.M., Madejón, E., Madejón, P., 2016. Effect of heavy metals and organic matter on root exudates (low molecular weight organic acids) of herbaceous species: an assessment in sand and soil conditions under different levels of contamination. Environ. Pollut. 216, 273-281.

Rosindell, J., Hubbell, S. P., Etienne, R.S., 2011. The unified neutral theory of biodiversity and biogeography at age ten. Trends Ecol. Evol. 26 (7), 340-348.

Sun, X., Kong, T., Häggblom, M.M., Kolton, M., Li, F., Dong, Y., Huang, Y., Li, B., Sun, W., 2020. Chemolithoautotropic diazotrophy dominates the nitrogen fixation process in mine tailings. Environ. Sci. Technol. 54 (10), 6082-6093.

Wang, L., Gong, L., Gan, D., Li, X., Yao, J., Wang, L., Qu, J., Cong, J., Zhang, Y., 2022. Diversity, function and assembly of the *Trifolium repens* L. root-associated microbiome under lead stress. J. Hazard. Mater. 438, 129510.

Xing, W., Gai, X., Ju, F., Chen, G., 2023. Microbial communities in tree root-compartment niches under Cd and Zn pollution: structure, assembly process and co-occurrence relationship. Sci. Total Environ. 860, 160273.

Xu, R., Li, B., Xiao, E., Young, L.Y., Sun, X., Kong, T., Dong, Y., Wang, Q., Yang, Z., Chen, L., Sun, W., 2020. Uncovering microbial responses to sharp geochemical gradients in a terrace contaminated by acid mine drainage. Environ. Pollut. 261, 114226.

Zhang, D., Guo, J., Xie, X., Zhang, Y., Jing, C., 2022. Acidity-dependent mobilization of antimony and arsenic in sediments near a mining area. J. Hazard. Mater. 426, 127790.

Zhu, F., Zhang, X., Guo, X., Yang, X., Xue, S., 2023. Root architectures differentiate the composition of organic carbon in bauxite residue during natural vegetation. Sci. Total Environ. 883, 163588.
