# Supplementary material for: Enriched rhizospheric functional microbiome may enhance adaptability of Artemisia lavandulaefolia and Betula luminifera in antimony mining areas
Source: Front Microbiol. 2024 Mar 21;15:1348054. doi: 10.3389/fmicb.2024.1348054 (PMC10993014; doi:10.3389/fmicb.2024.1348054)
Supplement: Supplementary file 5 [file Table_6.DOCX]

■ **Table Legends**

**Table S1** Topological structure of both plants’ rhizosphere soil microbial co-occurrence networks in control and mining areas.

**Table S2** Geochemical parameters collinearity screening (*VIF* < 10) of both plants’ rhizosphere soil in control and mining areas.

**Table S3** Genes abundance of both plants’ rhizosphere microbial secreted functional proteins in control and mining areas.

**Table S4** SNP data summary table

**Supplementary Tables**

**Table S1** Topological structure of both plants’ rhizosphere soil microbial co-occurrence networks in control and mining areas.

|  | Topological features | CARS + CBRS | | MARS + MBRS | |
| --- | --- | --- | --- | --- | --- |
|  |  | Empirical network | Random network | Empirical network | Random network |
| Bacteria | Nodes | 294 | 294 | 292 | 292 |
|  | Edges | 2638 | 2638 | 3628 | 3628 |
|  | Network diameter | 8.114 | 3.46 ± 0.051 | 6.444 | 3 ± 0.000 |
|  | Graph density | 0.061 | 0.061 ± 0.000 | 0.085 | 0.085 ± 0.000 |
|  | Mean clustering coefficient | 0.523 | 0.061 ± 0.002 | 0.64 | 0.085 ± 0.002 |
|  | Average path length | 2.616 | 2.252 ± 0.002 | 2.557 | 2.024 ± 0.002 |
|  | Positive Correlations (%) | 2224 | 2224 | 3373 | 3373 |
|  | Negative Correlations (%) | 414 | 414 | 255 | 255 |
| Fungi | Nodes | 138 | 138 | 292 | 292 |
|  | Edges | 628 | 628 | 3061 | 3061 |
|  | Network diameter | 10.564 | 4.11 ± 0.314 | 12.351 | 3 ± 0.000 |
|  | Graph density | 0.066 | 0.066 ± 0.000 | 0.072 | 0.072 ± 0.000 |
|  | Mean clustering coefficient | 0.638 | 0.067 ± 0.006 | 0.77 | 0.072 ± 0.002 |
|  | Average path length | 3.716 | 2.464 ± 0.006 | 3.979 | 2.133 ± 0.002 |
|  | Positive Correlations (%) | 602 | 602 | 3056 | 3056 |
|  | Negative Correlations (%) | 26 | 26 | 5 | 5 |

**Note**: 10000 random networks were generated by randomly rewiring all edges of the corresponding empirical network. These random networks have an equal number of nodes and connections. The displayed values represent the mean ± SD from the 10000 random networks.

**Table S2** Geochemical parameters collinearity screening (*VIF* < 10) of both plants’ rhizosphere soil in control and mining areas.

| Environmental parameter | *VIF* value containing AAS | *VIF* value removing AAS |
| --- | --- | --- |
| DOC | 1.34 | 1.26 |
| AP | 16.90 | 6.06 |
| AK | 2.84 | 2.05 |
| TSb | 24.13 | 7.66 |
| TAs | 26.08 | 6.34 |
| ASb | 25.97 | 8.28 |
| AAs | 24.34 | — |
| pH | 5.26 | 2.02 |

**Note**: DOC: dissoluble organic carbon, AP: available phosphorus, AK: available potassium, TSb: total antimony, TAs: total arsenic, ASb: available antimony, AAs: available arsenic.

**Table S3** Genes abundance of both plants’ rhizosphere microbial secreted functional proteins in control and mining areas.

|  | Total gene abundance | Gene abundance of secretion protein | Gene abundance of type III secretion system |
| --- | --- | --- | --- |
| CARS | 4338.915 | 917.6274 (21.15%) | 1492.3(34.39%) |
| MARS | 5412.484 | 1275.994(23.58%) | 1921.749 (35.51%) |
| CBRS | 4195.834 | 876.9265(20.90%) | 1470.884 (35.06%) |
| MBRS | 6122.542 | 1464.698 (23.92%) | 2293.478 (37.46%) |

**Note**: Secretion protein refers to the protein which is synthesized in the cell and secreted out of the cell to play a role. Its main feature is that there is a hydrophobic amino acid peptide fragment encoded by DNA at the 5' end, which is called signal peptide. Using signal peptide prediction tool Signal P, neural network and hidden Markov model are used to predict secreted protein. Type III secretion system (T3SS) is widely distributed in different bacteria, and it is the most complex secretion system known at present. The software EffectiveT3 is used to predict the input amino acid sequences, and each amino acid sequence is scored by its internal specific calculation model. The higher the score, the higher the reliability, and the sequence whose score is higher than the threshold is selected, which is the type III secretory system effect protein.

**Table S4** SNP data summary table

|  | SNP Number | Transition | Transversion | Ts/Tv | Heterozygosity Number | Homozygosity Number |
| --- | --- | --- | --- | --- | --- | --- |
| BC | 9 | 3 | 6 | 0.50 | 2 | 41 |
| BM | 43 | 19 | 25 | 0.76 | 43 | 0 |

**Note**: SNP Number: Number of detected single nucleotide polymorphism (SNP) sites, representing nucleotide variations between the experimental material and the reference genome; Transition: Number of transition SNPs; Transversion: Number of transversion SNPs; Ts/Tv: Ratio of transition SNPs (Transition) to transversion SNPs (Transversion); Heterozygosity Number: Total number of heterozygous SNP sites; Homozygosity Number: Total number of homozygous SNP sites. BC represents seedlings germinated from *Betula luminifera* seeds collected from the control area; BM represents seedlings germinated from *Betula luminifera* seeds collected from the mining area.
