# Supplementary material for: Enriched rhizospheric functional microbiome may enhance adaptability of Artemisia lavandulaefolia and Betula luminifera in antimony mining areas
Source: Front Microbiol. 2024 Mar 21;15:1348054. doi: 10.3389/fmicb.2024.1348054 (PMC10993014; doi:10.3389/fmicb.2024.1348054)
Supplement: Supplementary file 6 [file Table_10.DOCX]

■ **Figure Legends**

**Fig. S1.** Sample selection principle of shotgun metagenomics analysis based on MicroPITA analysis of both plants’ rhizosphere soil in control and mining areas.

**Fig. S2.** The microbial alpha-diversity index of both plants’ rhizosphere soil in control and mining areas.

**Fig. S3.** Bacterial phylum and genus-level community composition, and fungal phylum and genus-level community composition of both plants’ rhizosphere soil in control and mining areas.

**Fig. S4.** Venn diagram, and classification of generalists results revealed unique and shared rhizosphere microorganisms.

**Fig. S5.** Co-occurrence network of bacterial and fungal communities based on correlation analysis of both plants’ rhizosphere soil in control and mining area.

**Fig. S6.** Stability of the microbial co-occurrence network.

**Fig. S7.** Microbial community assembly process measurements by the normalized stochasticity ratio (NST), and the relative abundance of stress - tolerant bacterial and fungal function prediction.

**Fig. S8.** Abundance of ectomycorrhizal fungi in the rhizosphere soil.

**Fig. S9.** Ordinal regression analysis showed the influence of total heavy metals on bacterial communities of both plants’ rhizosphere soil in control and mining areas.

**Fig. S10.** Ordinal regression analysis showed the influence of available heavy metals on fungal communities of both plants’ rhizosphere soil in control and mining areas.

**Fig. S11.** Ordinal regression analysis showed the influence of total heavy metals on fungal communities of both plants’ rhizosphere soil in control and mining areas.

**Fig. S12.** Ordinal regression analysis showed the influence of available heavy metals on fungal communities of both plants’ rhizosphere soil in control and mining areas.

**Fig. S13.** Changes of genes abundance related to carbon cycle of both plants’ rhizosphere soil in control and mining areas.

**Fig. S14.** Statistical analysis genes of Sb/As-resistance – related, carbon cycle-related, nitrogen cycle-related, phosphorus cycle-related and sulfur cycle-related of *B. luminifera* rhizosphere soil in control and mining areas.

**Fig. S15.** Statistical analysis genes of Sb/As-resistance – related, carbon cycle-related, nitrogen cycle-related, phosphorus cycle-related and sulfur cycle-related of *A. lavandulaefolia* rhizosphere soil in control and mining areas.

**Fig. S16.** Statistical analysis genes of Sb/As-resistance – related, carbon cycle-related, nitrogen cycle-related, phosphorus cycle-related and sulfur cycle-related of *A. lavandulaefolia* and *B. luminifera* rhizosphere soil in control aera.

**Fig. S17.** Statistical analysis genes of Sb/As-resistance – related, carbon cycle-related, nitrogen cycle-related, phosphorus cycle-related and sulfur cycle-related of *A. lavandulaefolia* and *B. luminifera* rhizosphere soil in mining aera.

**Fig. S18**. Changes of genes abundance related to bacterial chemotaxis system, and quorum sensing (QS index) of both plants’ rhizosphere soil in control and mining areas.

**Fig. S19.** Statistical analysis of bacterial chemotaxis system related genes of both plants’ rhizosphere soil in control and mining areas

**Fig. S20.** Statistical analysis of bacterial quorum sensing (QS index) of both plants’ rhizosphere soil in control and mining areas.

**Fig. S21.** Notes on probiotics of both plants’ rhizosphere soil in control and mining areas.

**Fig. S22.** Microbial contribution of Sb/As-resistance related genes of both plants’ rhizosphere soil in control and mining areas.

**Fig. S23.** Changes of genes abundance related to carbon cycle and microbial contribution of both plants’ rhizosphere soil in control and mining areas.

**Fig. S24.** Microbial contribution of nitrogen cycle-related genes of both plants’ rhizosphere soil in control and mining areas.

**Fig. S25.** Microbial contribution of phosphorus cycle-related genes of both plants’ rhizosphere soil in control and mining areas.

**Fig. S26.** Microbial contribution of sulfur cycle-related genes of both plants’ rhizosphere soil in control and mining areas.

**Fig. S27.** Microbial contribution of chemotaxis system - related genes of both plants’ rhizosphere soil in control and mining areas.

**Fig. S28.** Correlation analysis between plant probiotics and environmental parameters. The legend on the right indicates the pearson correlation coefficients.

**Fig. S29**. Phylogenetic tree based on the evolutionary relationships among rhizosphere microbial species, revealing the phylogenetic affinity of native dominant plant rhizosphere microorganisms from a molecular evolution perspective.

**Fig. S30**. *Betula luminifera* in mining area and control area, and phylogenetic tree of chloroplast genomes of *Betula luminifera* from the control and mining areas.


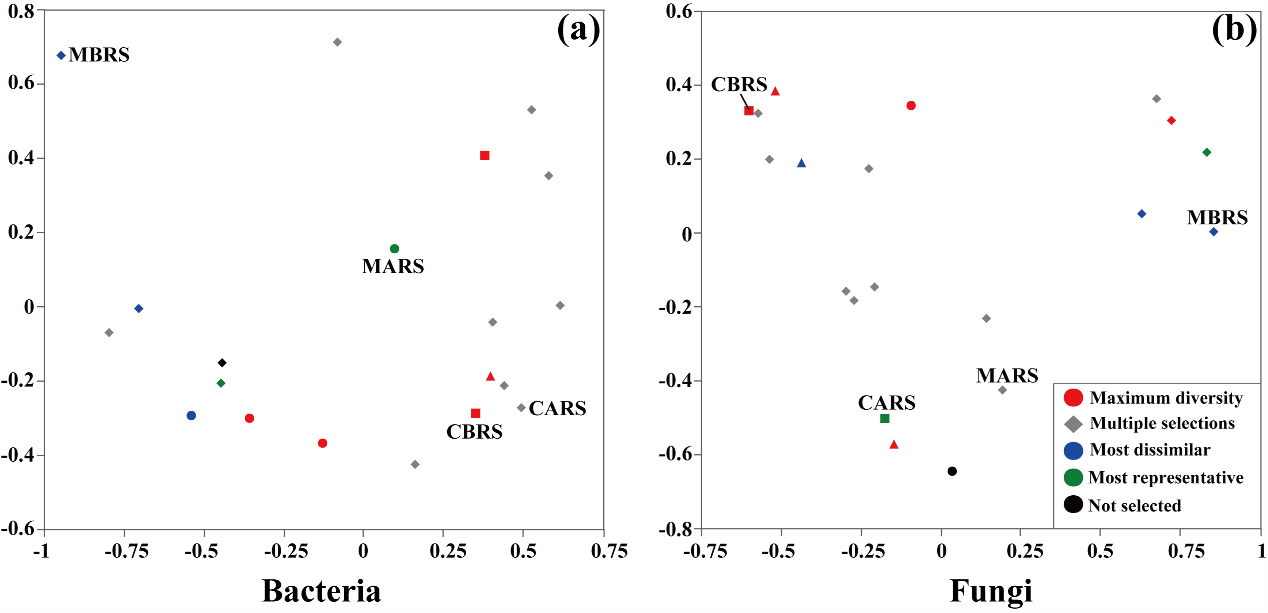


**Fig. S1.** Sample selection principle of shotgun metagenomics analysis based on MicroPITA analysis (a, b) of both plants’ rhizosphere soil in control and mining areas.

**Note:** According to the samples with the highest diversity of α (Maximum diversity), the most extreme species composition (Most dissimilar), can basically reflect the characteristics of the overall species composition (Most representative), all selected by the above three methods (Multiple selections), and none by the above three methods (Not selected) are used for the preliminary screening of samples. Then, combined with the total antimony content in rhizosphere soil, secondary screening was conducted.


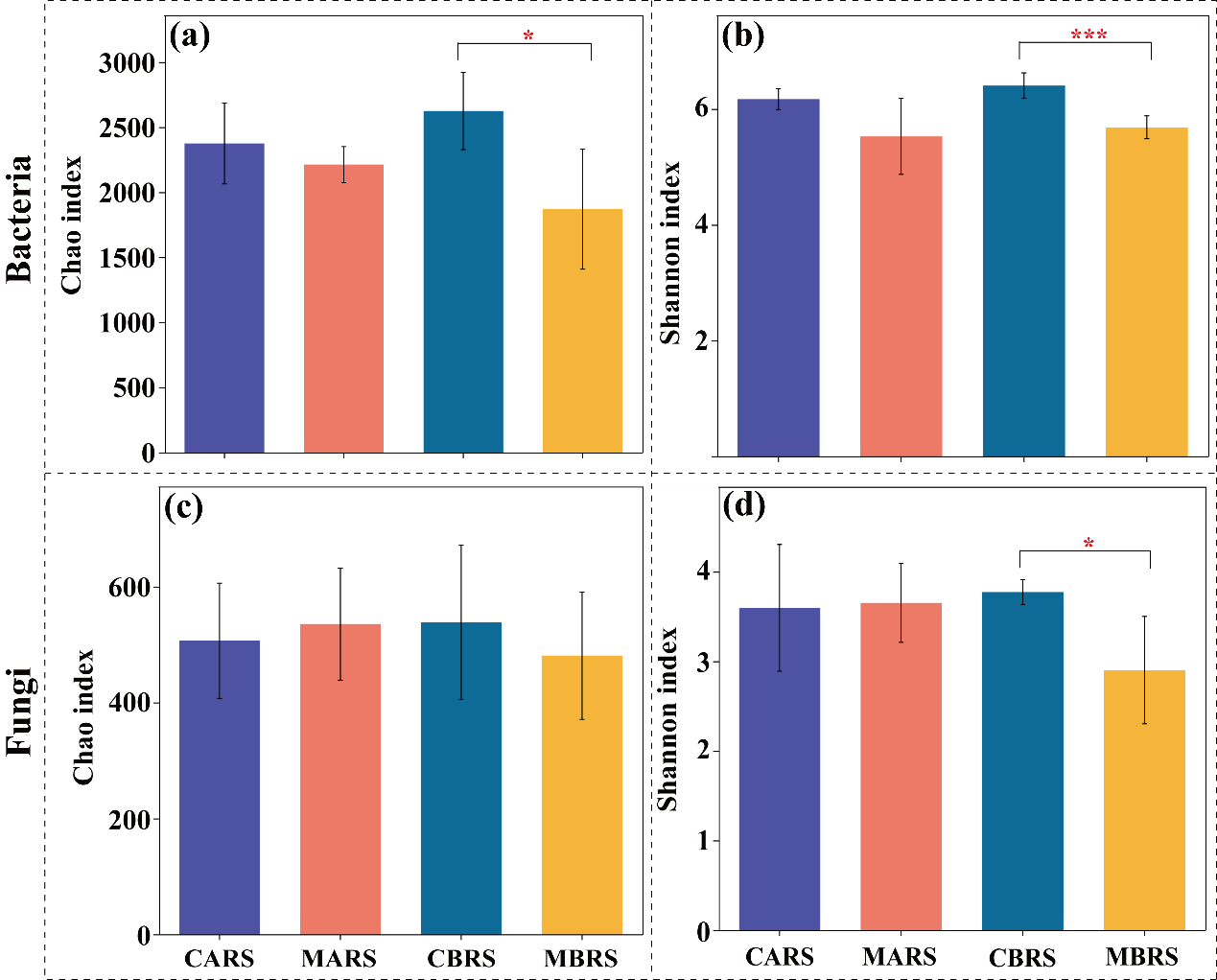


**Fig. S2.** The microbial alpha-diversity index (a, b, c and d) of both plants’ rhizosphere soil in control and mining areas.

**Note:** CARS is *Artemisia lavandulaefolia* rhizosphere soil in control area, MARS is *Artemisia lavandulaefolia* rhizosphere soil in mining area, CBRS is *Betula luminifera* rhizosphere soil in control area, MBRS is *Betula luminifera* rhizosphere soil in mining area, * is *P* < 0.05, ** is *P* < 0.01, *** is *P* < 0.001, n = 5, similarly hereinafter.


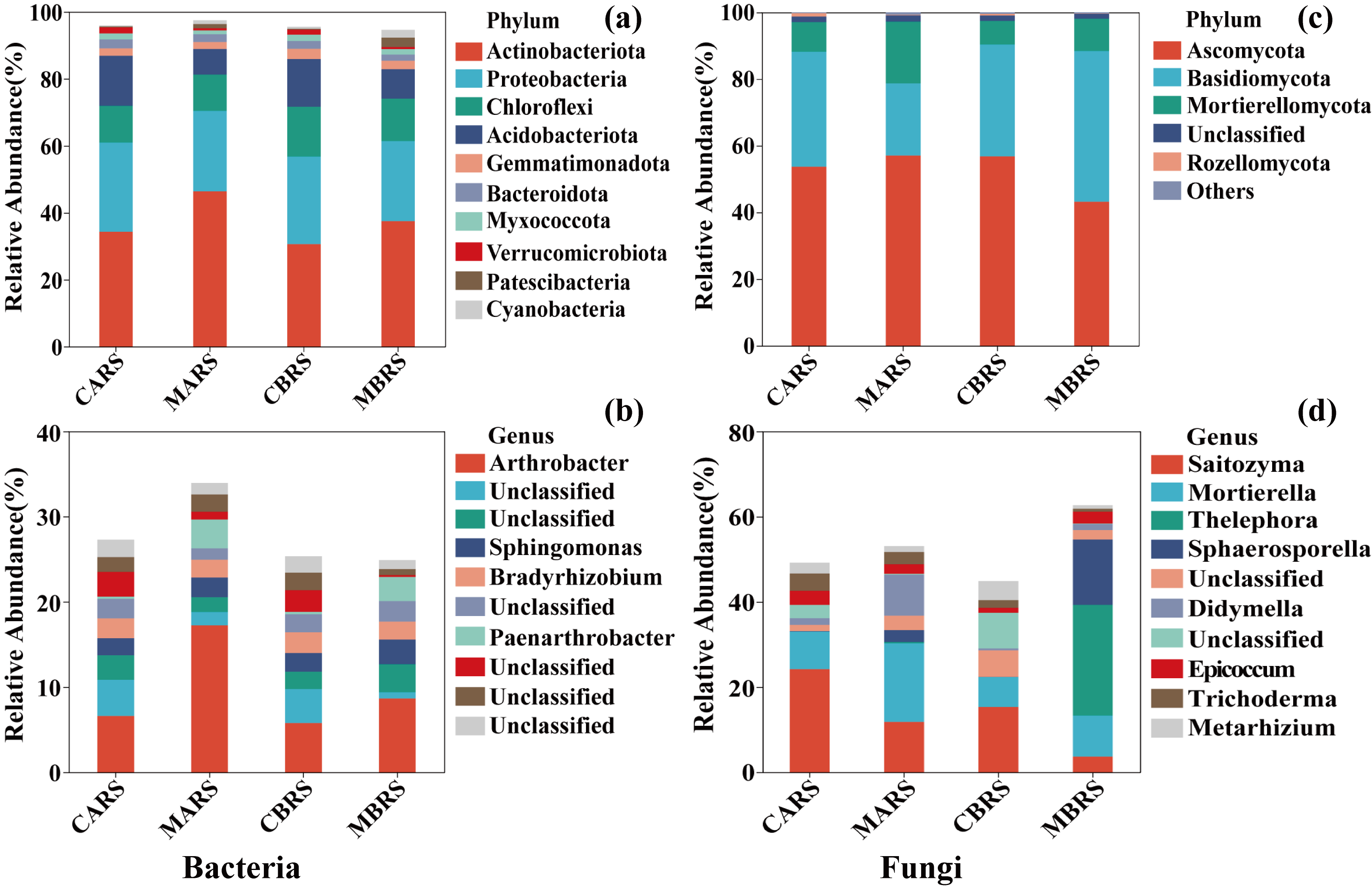


**Fig. S3**. Bacterial phylum and genus-level community composition (a and b), and fungal phylum and genus-level community composition (c and d) of both plants’ rhizosphere soil in control and mining areas.

**Note:** CARS is *A. lavandulaefolia* rhizosphere soil in control area, MARS is *A. lavandulaefolia* rhizosphere soil in mining area, CBRS is *B. luminifera* rhizosphere soil in control area, MBRS is *B. luminifera* rhizosphere soil in mining area, similarly hereinafter.


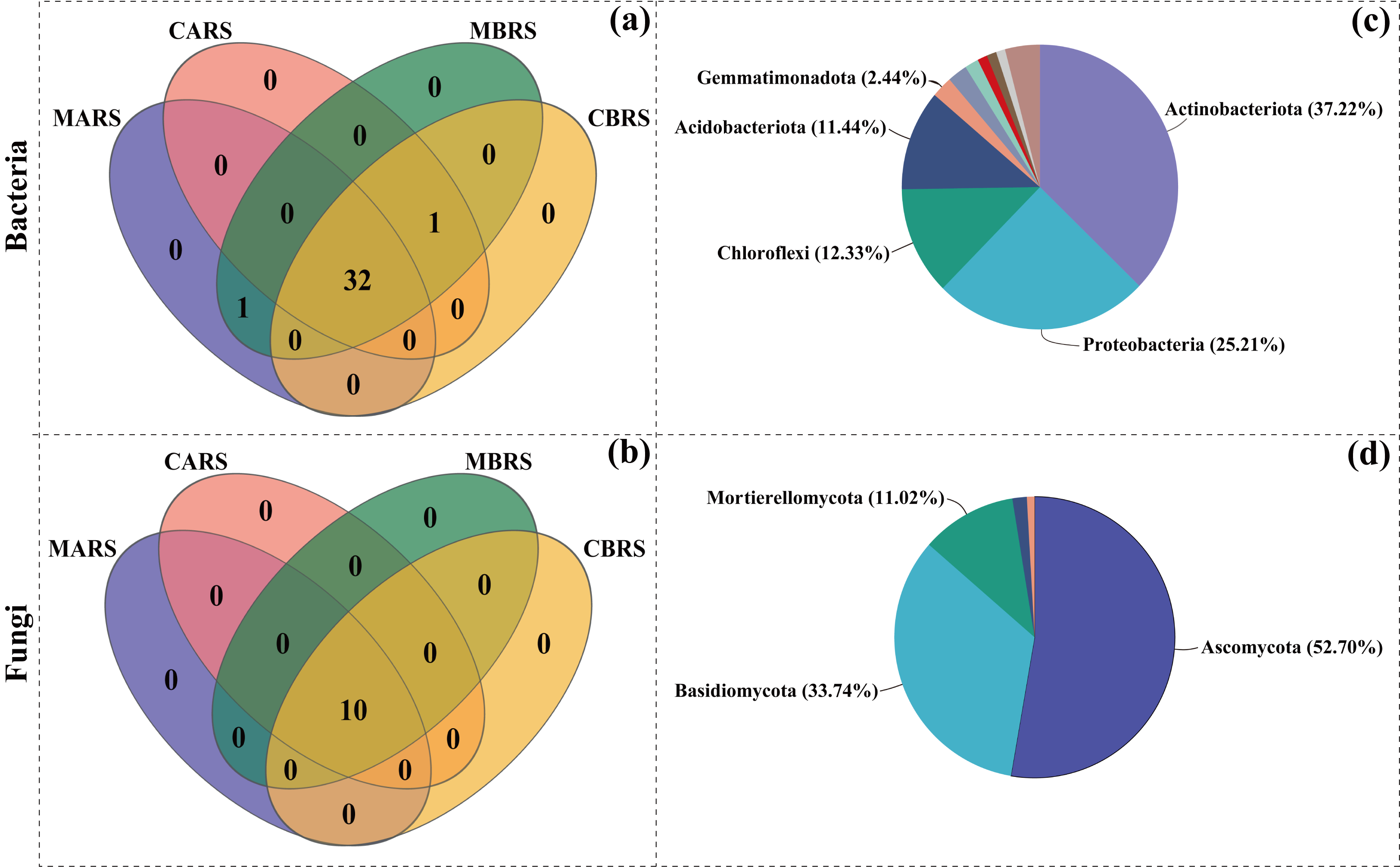


**Fig. S4.** Venn diagram (a and b), and classification of generalists (c and d) results revealed unique and shared rhizosphere microorganisms.


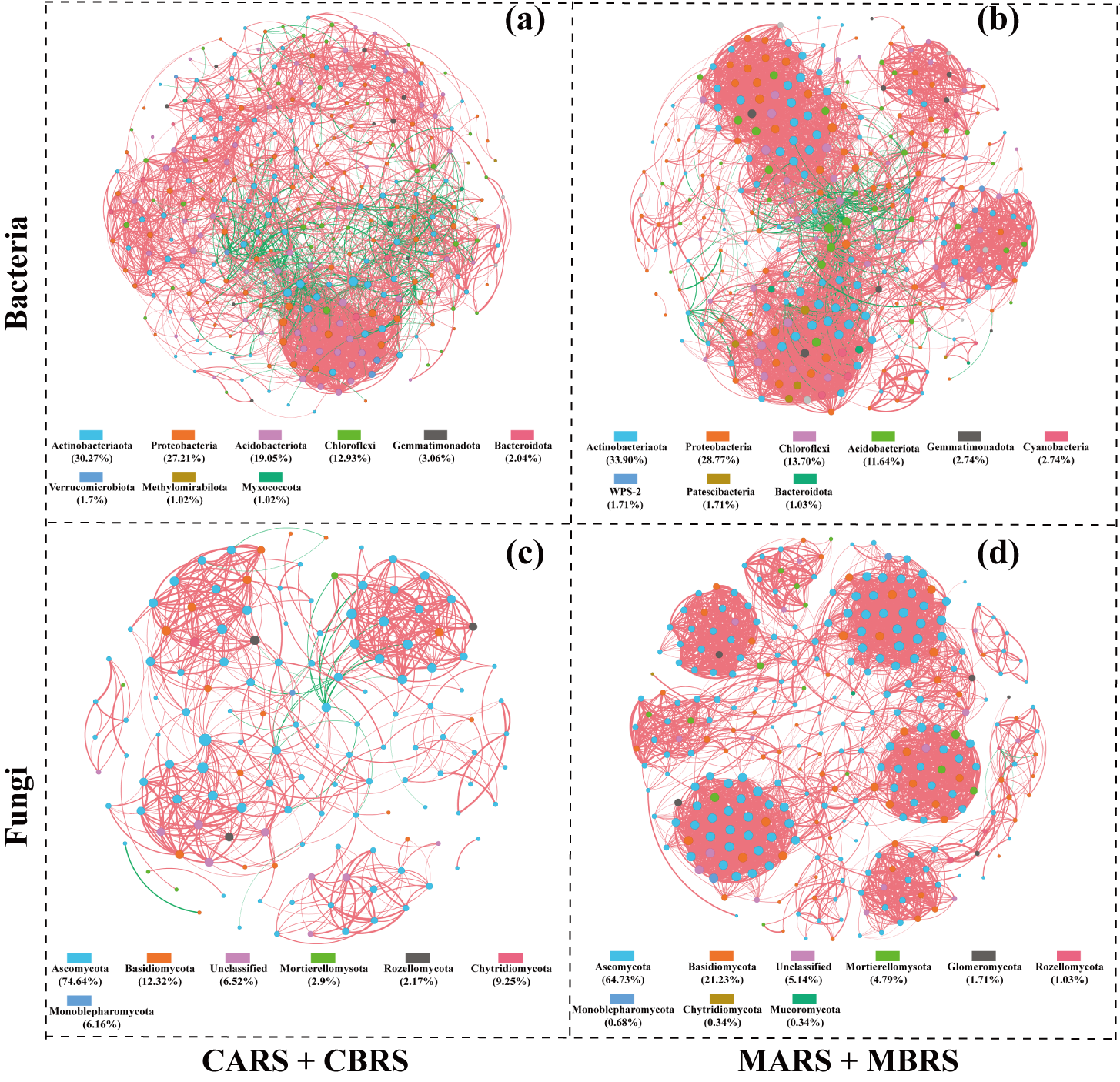


**Fig. S5.** Co-occurrence network of bacterial (a and b) and fungal (c and d) communities based on correlation analysis of both plants’ rhizosphere soil in control and mining area.

**Note:** The nodes in the network are OTUs (top 300), and they are colored according to phylum. Red line and green line segments indicate significantly positive and negative correlations, respectively. The connections indicate strong (Pearson's r > 0.6) and significant (*P* < 0.01) correlations, and the size of each node indicates degree.


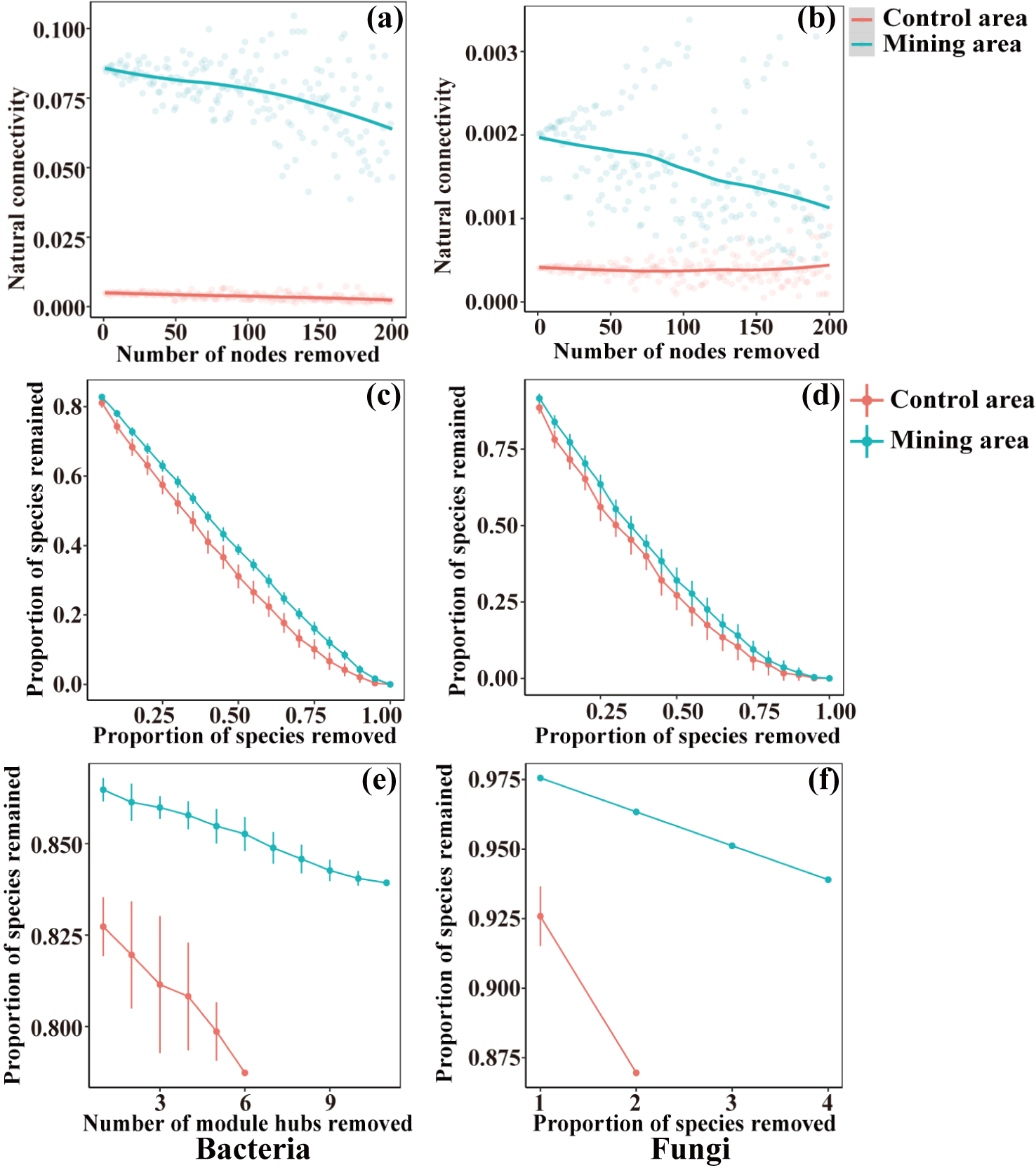


**Fig. S6.** Stability of the microbial co-occurrence network. (a) and (b) depict the ability of the co-occurrence network to maintain connectivity after the removal of certain nodes. (c) and (d) illustrate the proportion of remaining species in the co-occurrence network after the removal of specific species. (e) and (f) show the proportion of remaining species in the co-occurrence network after the removal of certain module hubs.


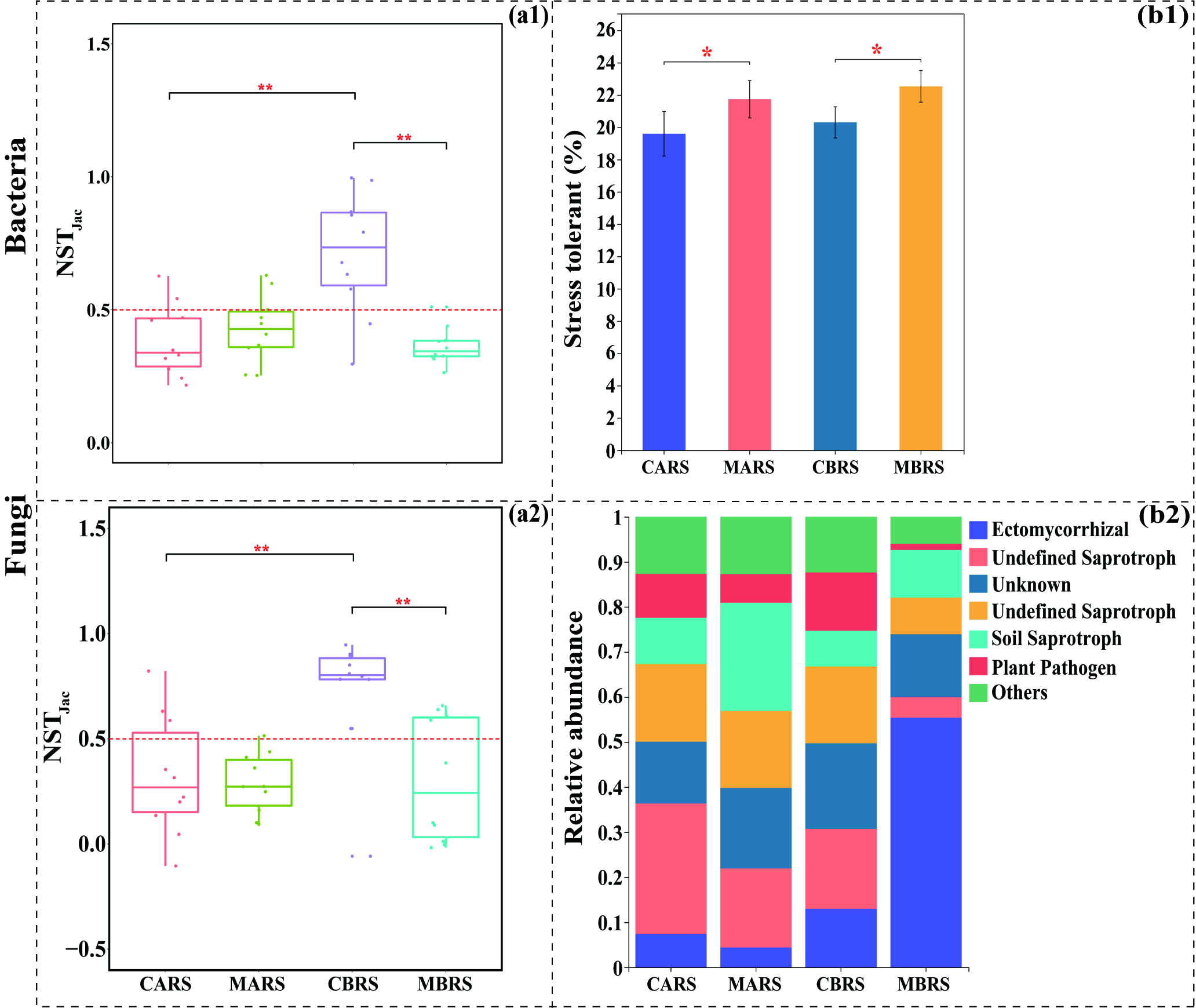


**Fig. S7.** Microbial community assembly process measurements by the normalized stochasticity ratio (NST) (a1 and a2); The relative abundance of stress - tolerant bacterial(b1) and fungal function prediction (b2).


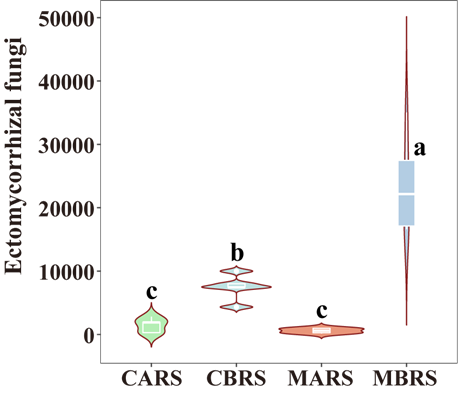


**Fig. S8.** Abundance of ectomycorrhizal fungi in the rhizosphere soil.

**Note:** Kruskal-Wallis H test (Does not conforming to normality distribution and homogeneity of variance) was utilized to assess significant differences among multiple groups of samples. CARS: *A. lavandulaefolia* rhizosphere soil in control area, MARS: *A. lavandulaefolia* rhizosphere soil in mining area, CBRS: *B. luminifera* rhizosphere soil in control area, MBRS is *B. luminifera* rhizosphere soil in mining area. Different lowercase letters indicate significant differences in the abundance of ectomycorrhizal fungi among different treatments (*P* < 0.05).


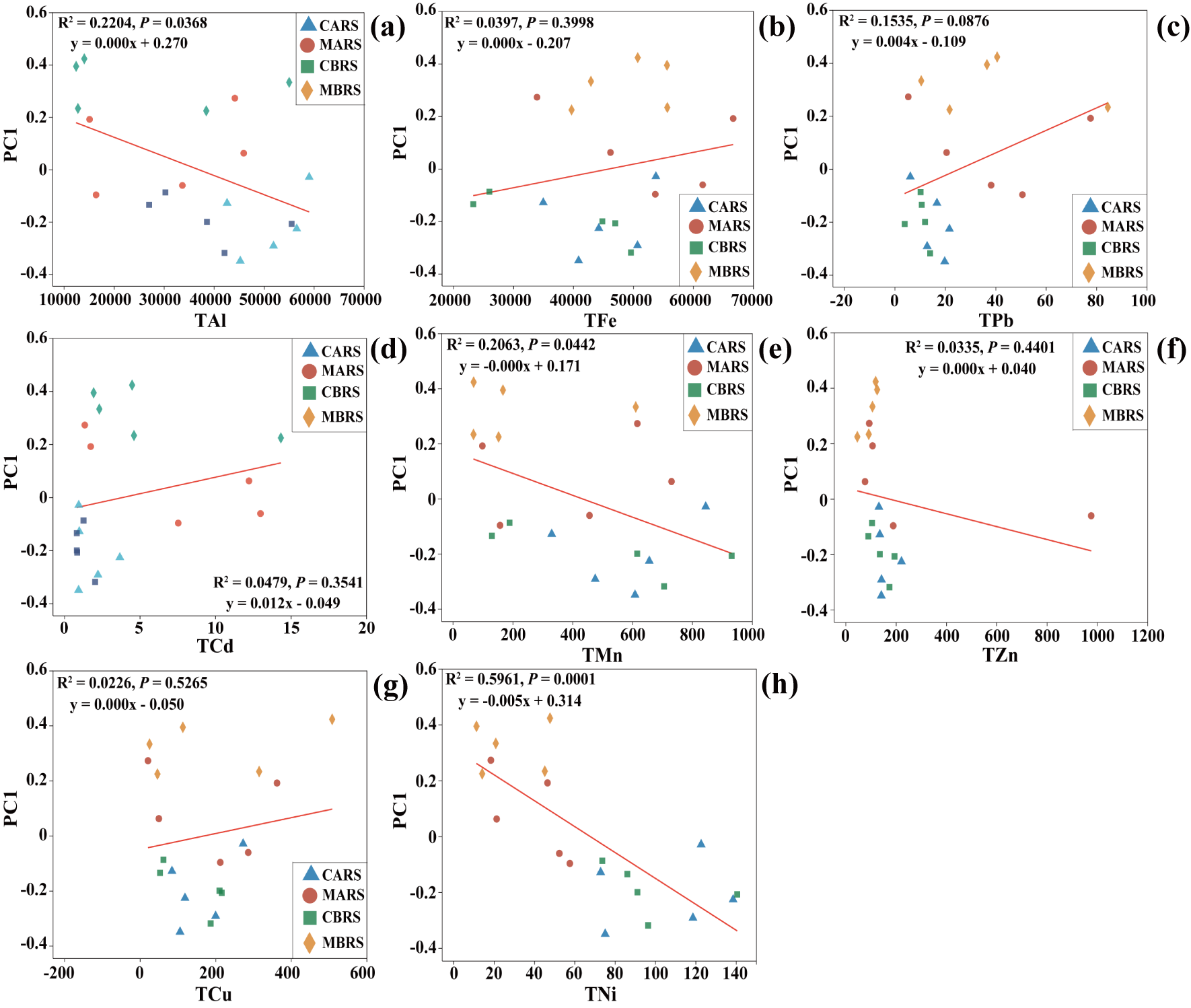


**Fig. S9.** Ordinal regression analysis showed the influence of total heavy metals on bacterial communities of both plants’ rhizosphere soil in control and mining areas.

**Note:** TAl: total aluminum, TFe: total iron, TPb: total lead, TCd: total cadmium, TMn: total manganese, TZn: total zinc, TCu: total copper, TNi: total nickel.


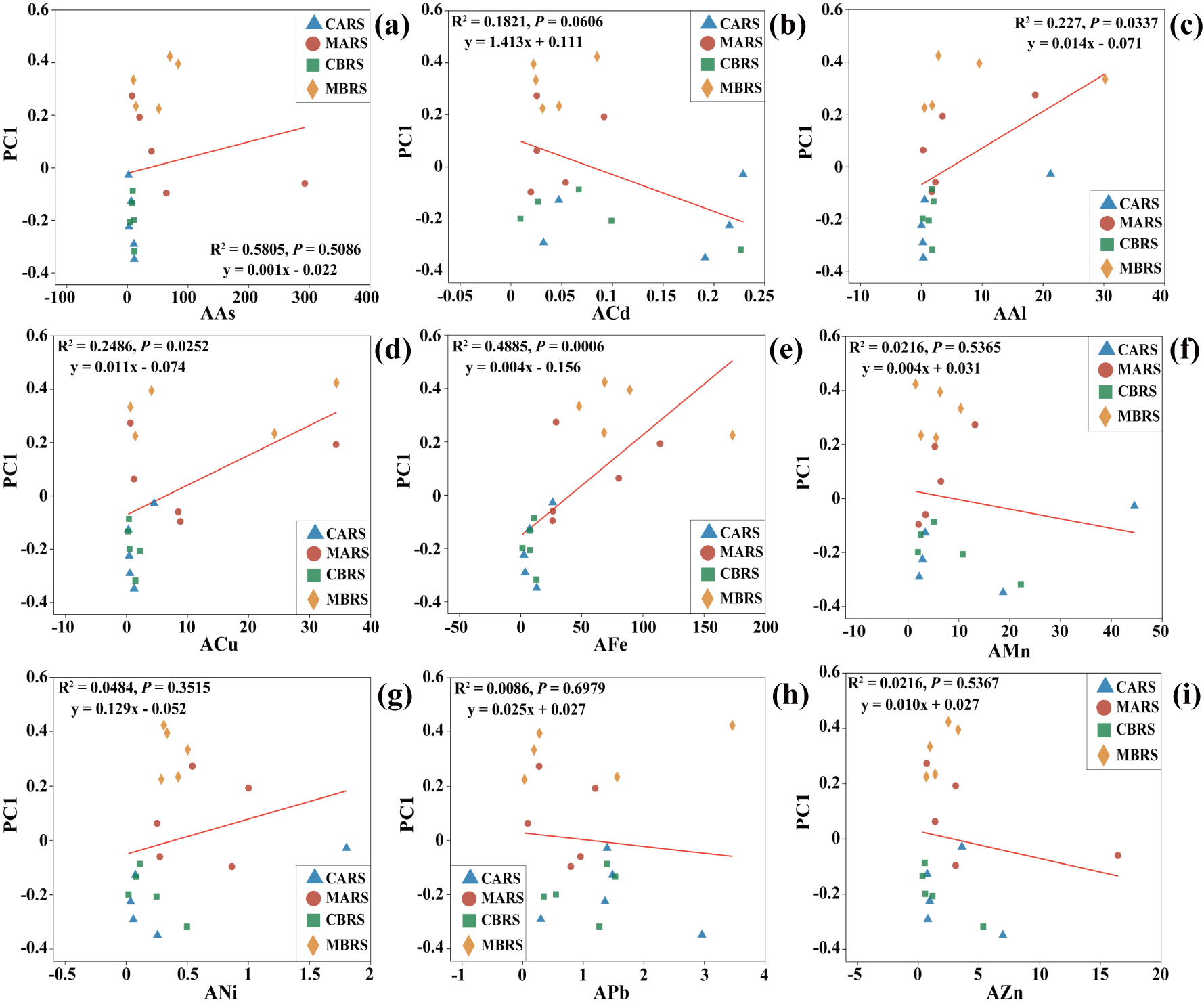


**Fig. S10**. Ordinal regression analysis showed the influence of available heavy metals on bacterial communities of both plants’ rhizosphere soil in control and mining areas.

**Note:** AAs: available arsenic, ACd: available cadmium, AAl: available aluminum, ACu: available copper, AFe: available iron, AMn: available manganese, ANi: available nickel, APb: available lead, AZn: available zinc.


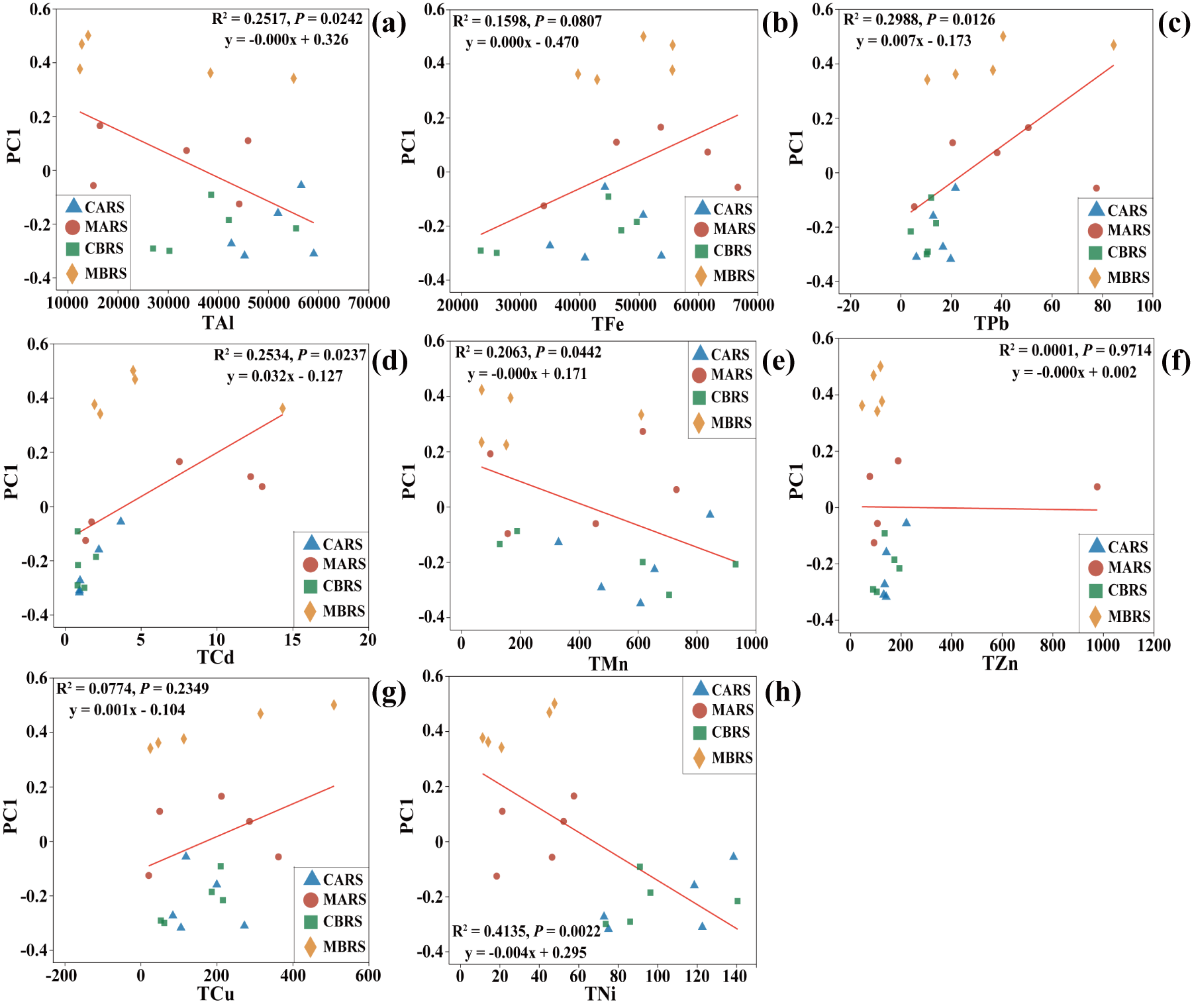


**Fig. S11**. Ordinal regression analysis showed the influence of total heavy metals on fungal communities of both plants’ rhizosphere soil in control and mining areas.

**Note:** TAl: total aluminum, TFe: total iron, TPb: total lead, TCd: total cadmium, TMn: total manganese, TZn: total zinc, TCu: total copper, TNi: total nickel.


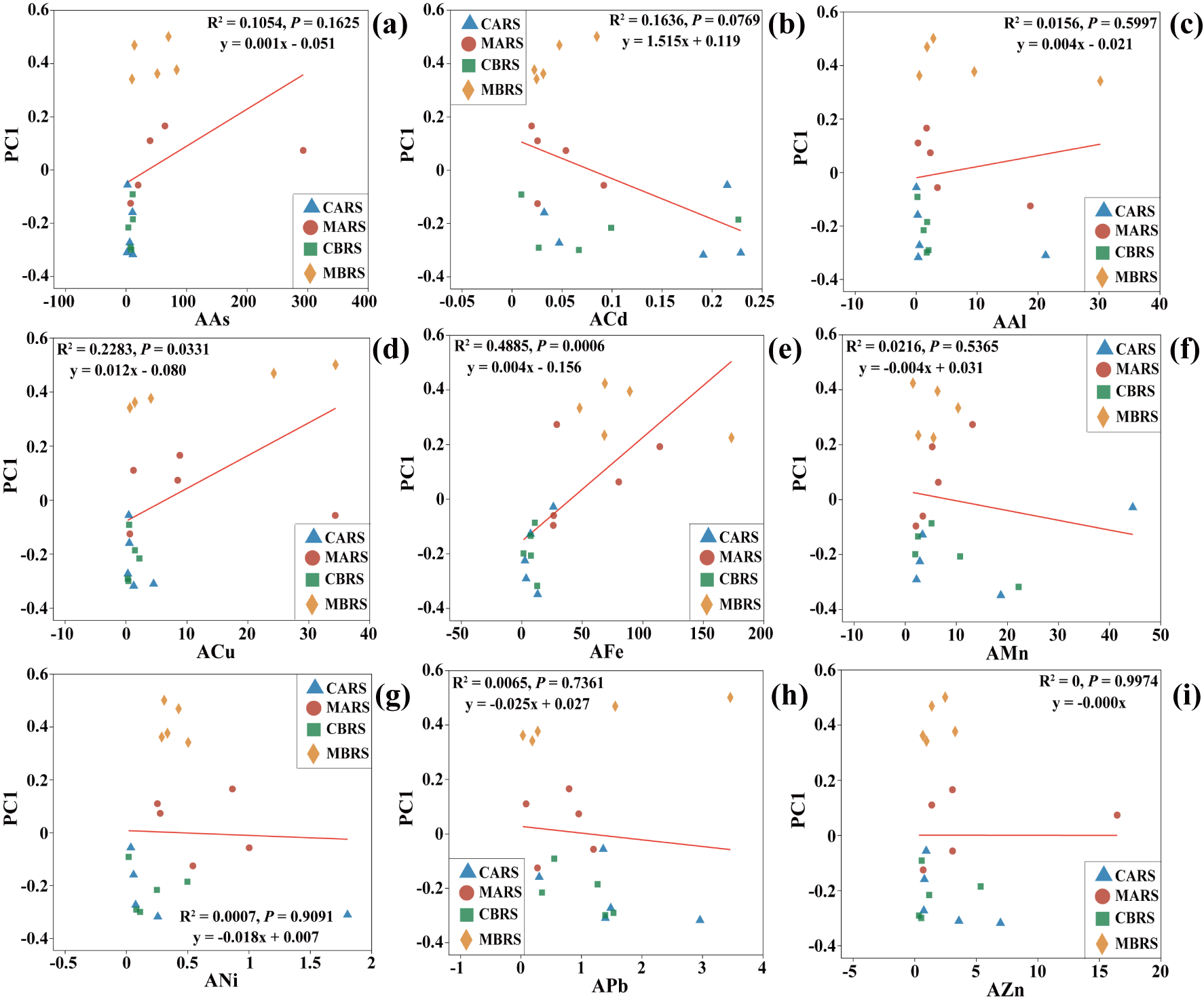


**Fig. S12**. Ordinal regression analysis showed the influence of available heavy metals on fungal communities of both plants’ rhizosphere soil in control and mining areas.

**Note:** AAs: available arsenic, ACd: available cadmium, AAl: available aluminum, ACu: available copper, AFe: available iron, AMn: available manganese, ANi: available nickel, APb: available lead, AZn: available zinc.


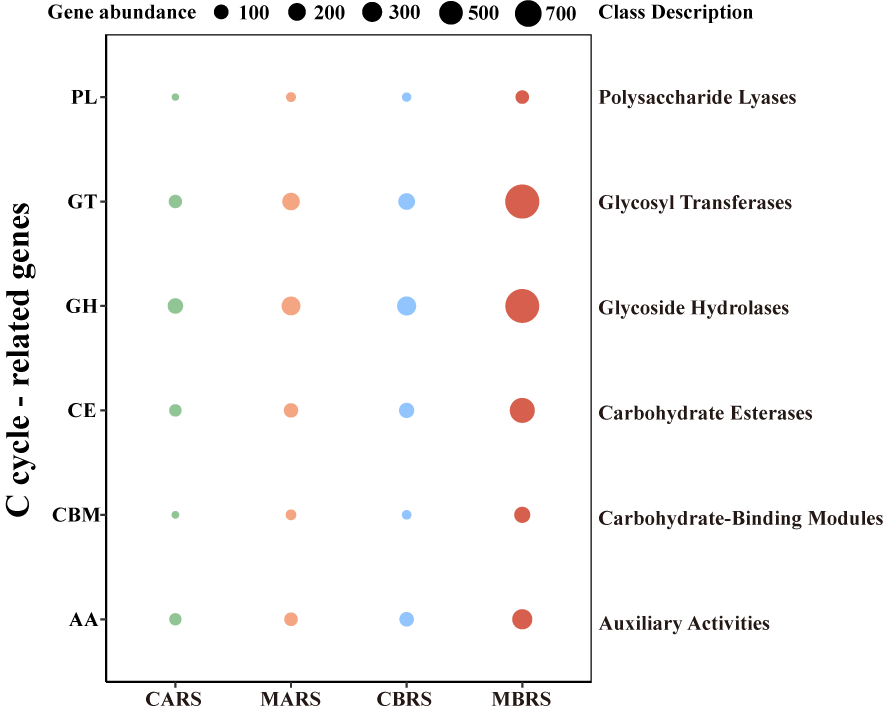


**Fig. S13.** Changes of genes abundance related to carbon cycle of both plants’ rhizosphere soil in control and mining areas.

**Note**: According to the similarity of amino acid sequences in protein domain, carbohydrate active enzymes from different species can be divided into Glycoside Hydrolases (GH), Glycosyl Transferases (GT), Polysaccharide Lyases (PL), Carbohydrate Esterases (CE), Carbohydrate-Binding Modules (CBM) and Auxiliary Activities (AA) are six protein families.


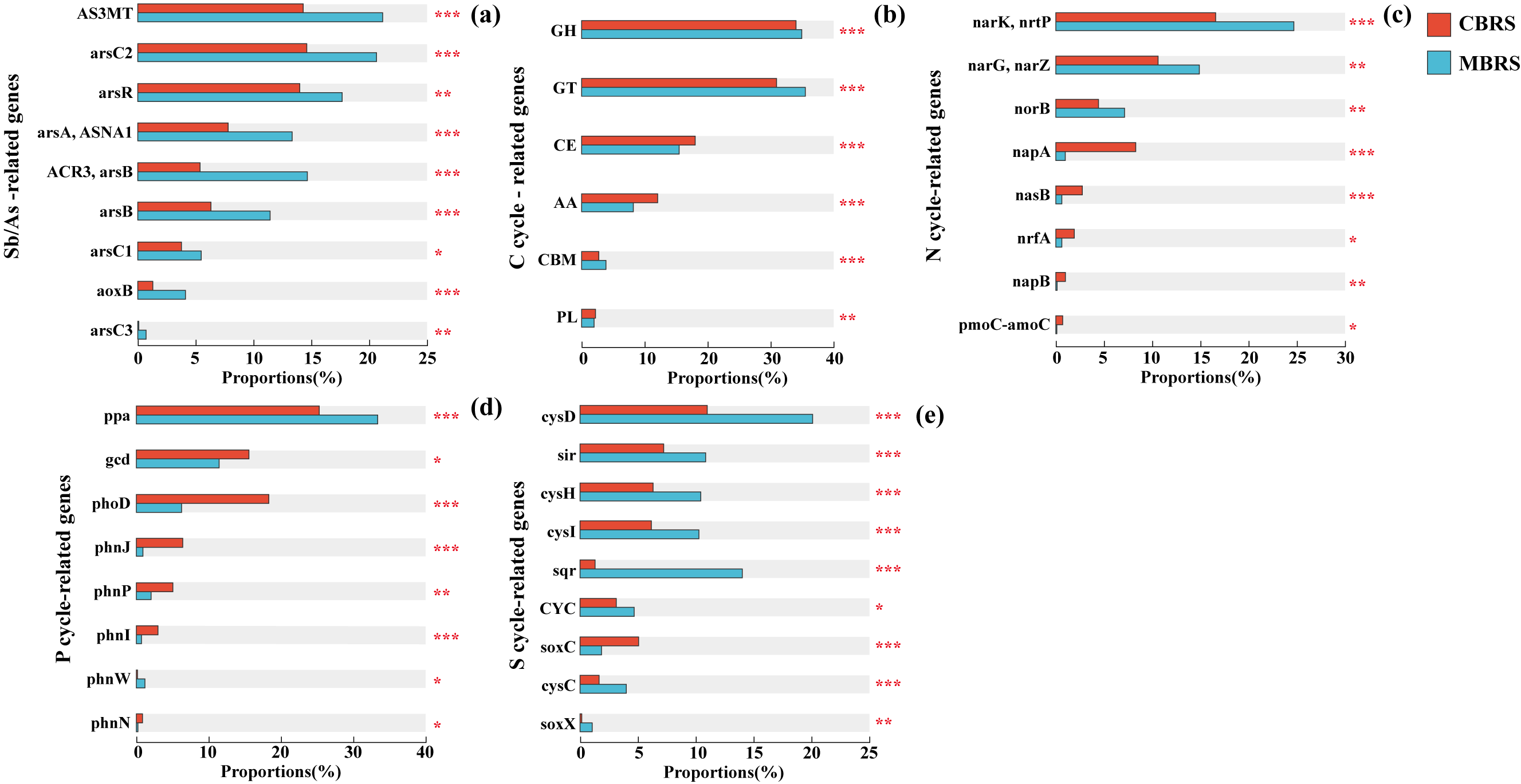


**Fig. S14.** Statistical analysis genes of Sb/As-resistance – related (a), carbon cycle-related (b), nitrogen cycle-related (c), phosphorus cycle-related (d) and sulfur cycle-related (e) of *B. luminifera* rhizosphere soil in control and mining areas.

**Note**: The Fisher's exact test was employed to test the significant between differences two samples (two-tailed test, fdr multiple test to correct *P*-value, the confidence interval was calculated by Newcombe-Wilson). The figure shows only the genes with significant differences (*P* <0.05), similarly hereinafter.


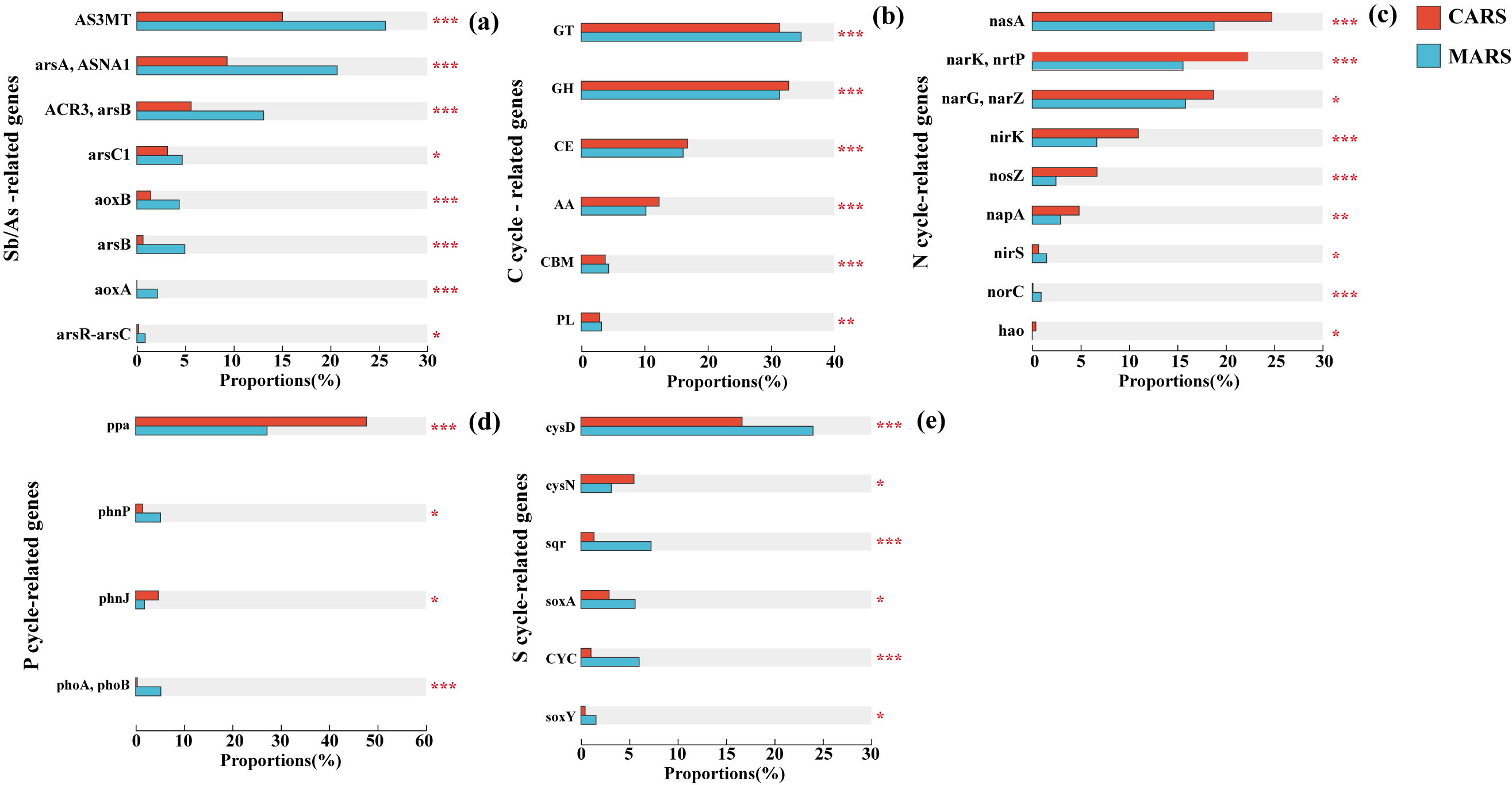


**Fig. S15.** Statistical analysis genes of Sb/As-resistance – related (a), carbon cycle-related (b), nitrogen cycle-related (c), phosphorus cycle-related (d) and sulfur cycle-related (e) of *A. lavandulaefolia* rhizosphere soil in control and mining areas.

**Note**: The Fisher's exact test was employed to test the significant between differences two samples (two-tailed test, fdr multiple test to correct *P*-value, the confidence interval was calculated by Newcombe-Wilson). The figure shows only the genes with significant differences (*P* <0.05), similarly hereinafter.


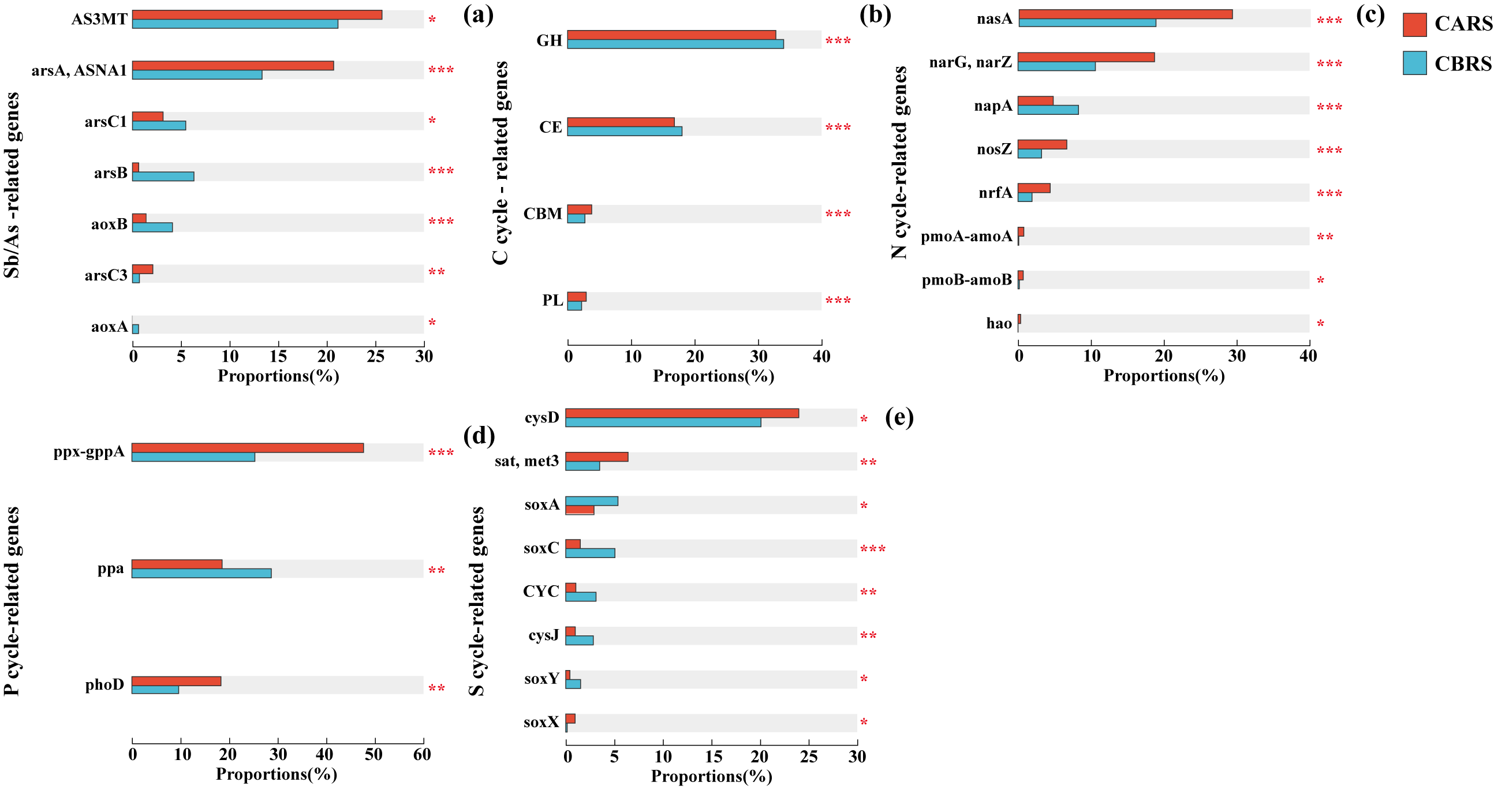


**Fig. S16.** Statistical analysis genes of Sb/As-resistance – related (a), carbon cycle-related (b), nitrogen cycle-related (c), phosphorus cycle-related (d) and sulfur cycle-related (e) of *A. lavandulaefolia* and *B. luminifera* rhizosphere soil in control aera.

**Note**: The Fisher's exact test was employed to test the significant between differences two samples (two-tailed test, fdr multiple test to correct *P*-value, the confidence interval was calculated by Newcombe-Wilson). The figure shows only the genes with significant differences (*P* <0.05), similarly hereinafter.


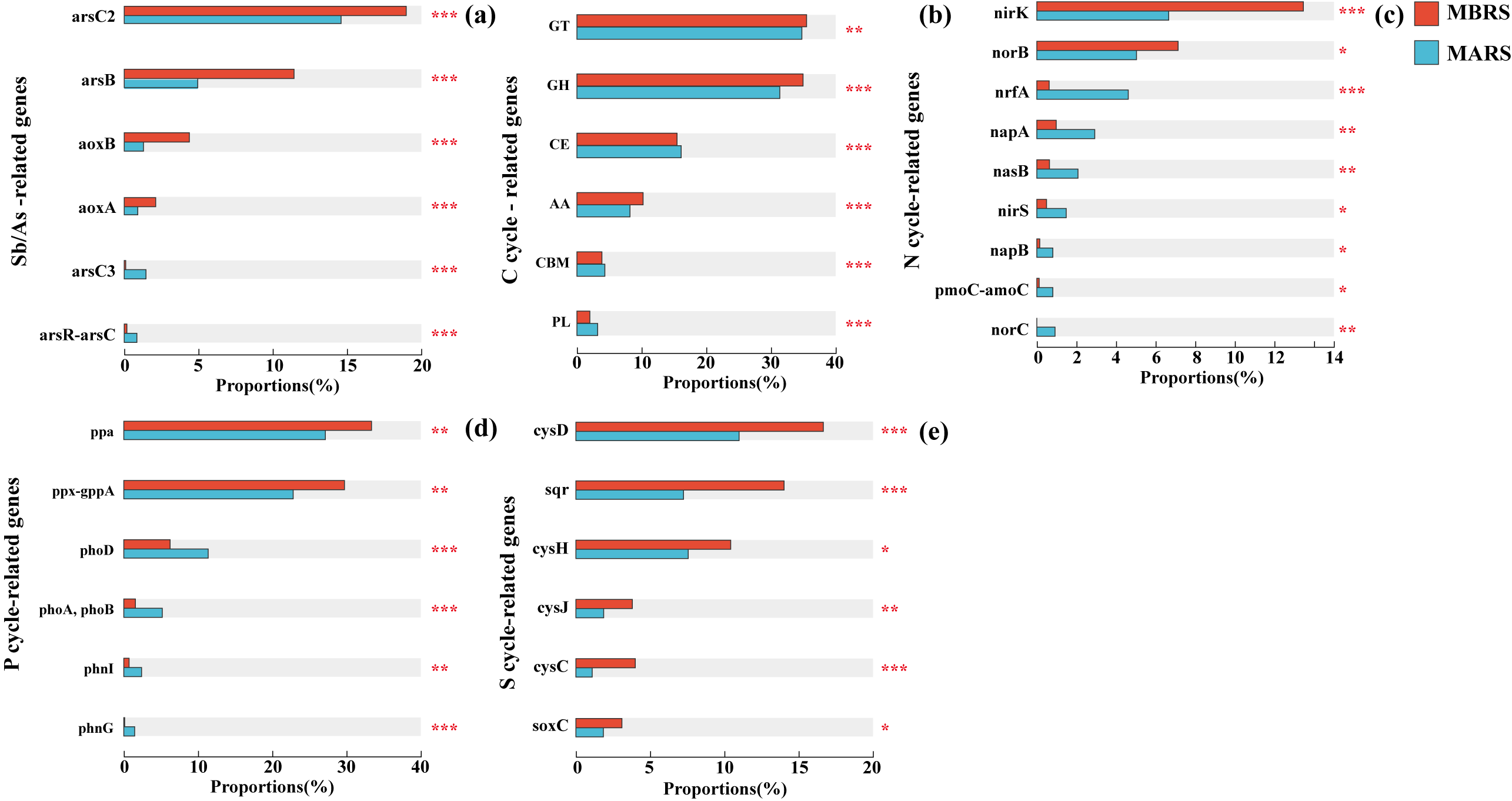


**Fig. S17.** Statistical analysis genes of Sb/As-resistance – related (a), carbon cycle-related (b), nitrogen cycle-related (c), phosphorus cycle-related (d) and sulfur cycle-related (e) of *A. lavandulaefolia* and *B. luminifera* rhizosphere soil in mining aera.

**Note**: The Fisher's exact test was employed to test the significant between differences two samples (two-tailed test, fdr multiple test to correct *P*-value, the confidence interval was calculated by Newcombe-Wilson). The figure shows only the genes with significant differences (*P* <0.05), similarly hereinafter.


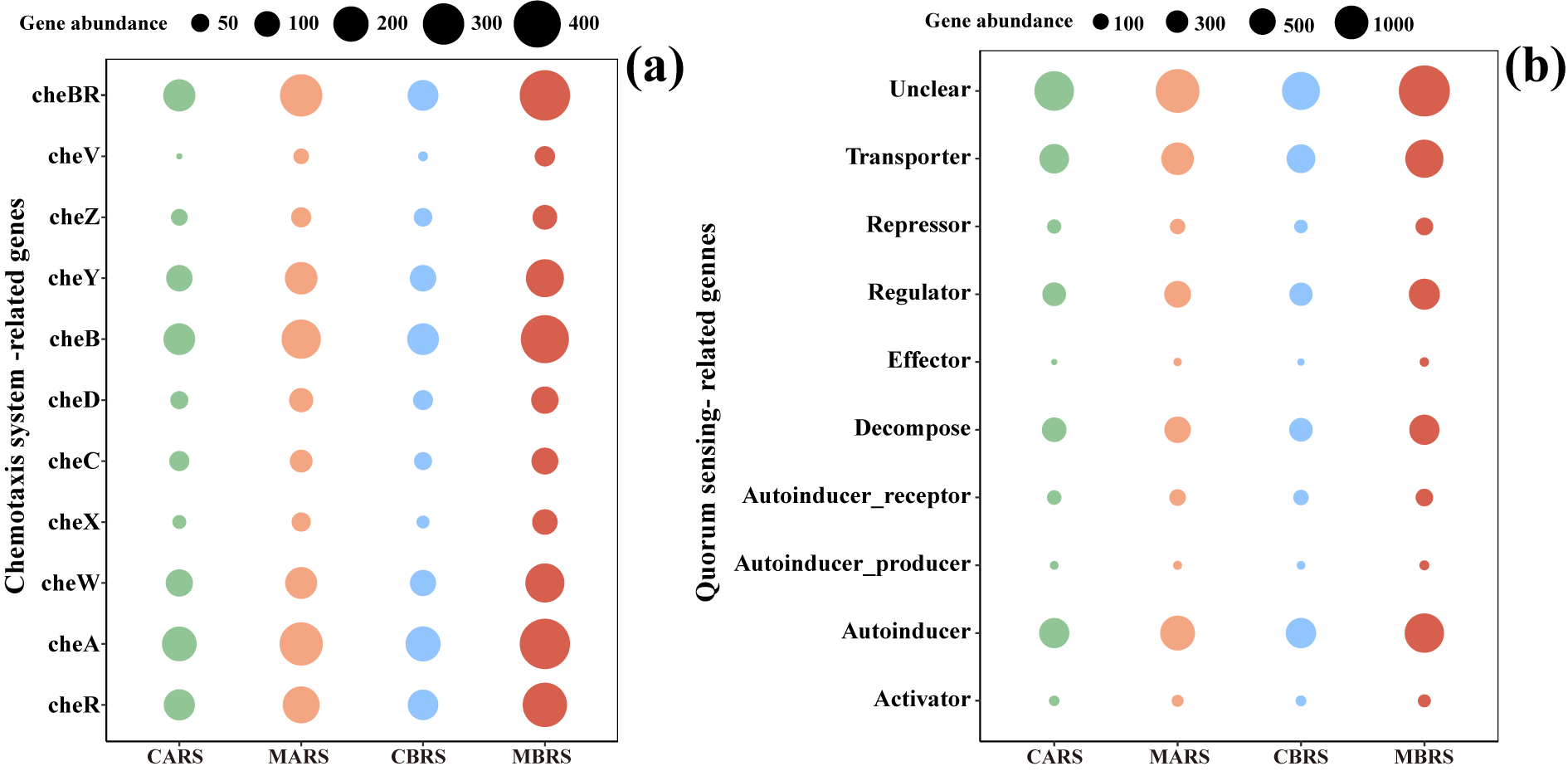


**Fig. S18.** Changes of genes abundance related to bacterial chemotaxis system (b), and quorum sensing (QS index) (a) of both plants’ rhizosphere soil in control and mining areas.

**Note**: See Fig. S19 and Fig. S20 for statistical difference analysis of genes.


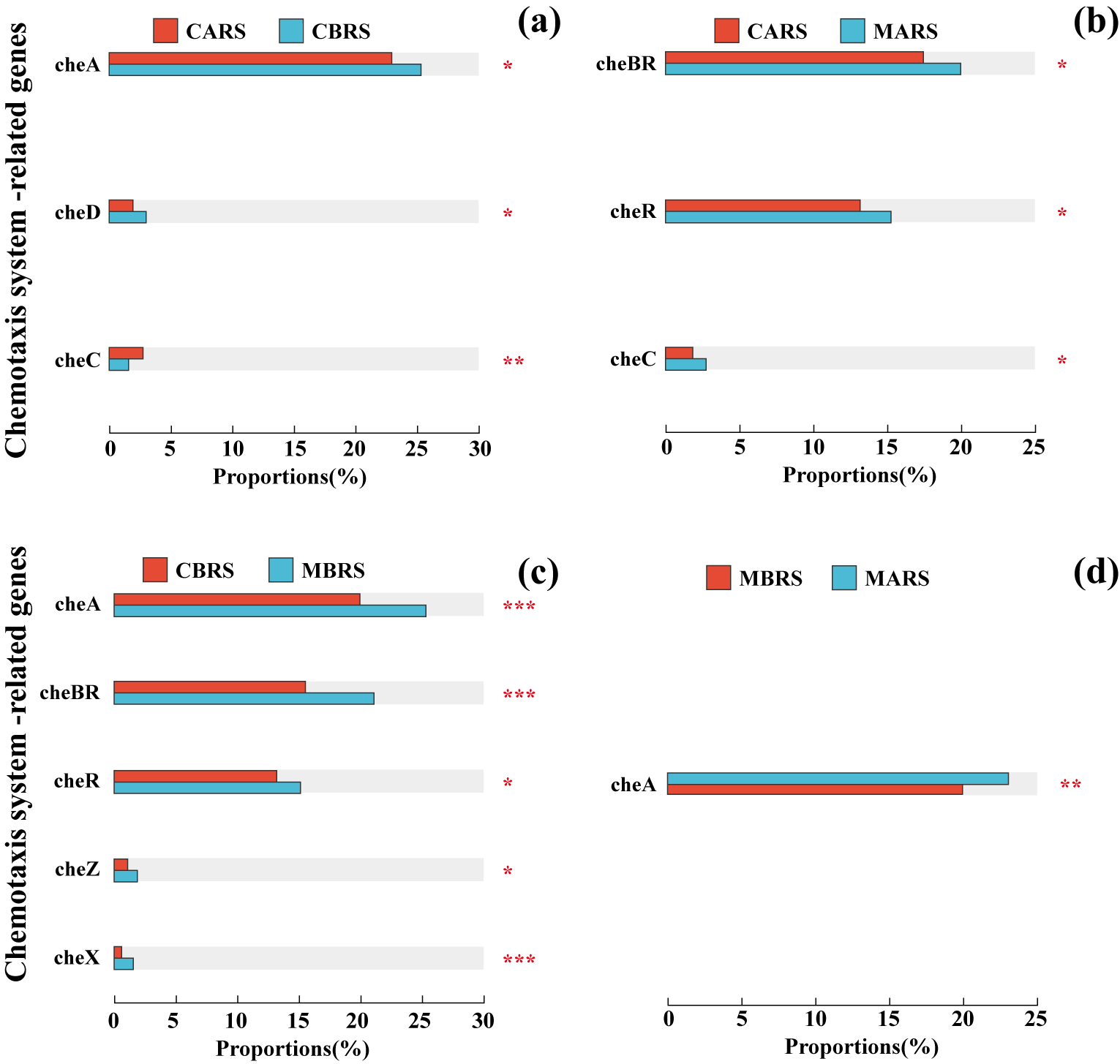


**Fig. S19.** Statistical analysis of bacterial chemotaxis system related genes of both plants’ rhizosphere soil in control and mining areas


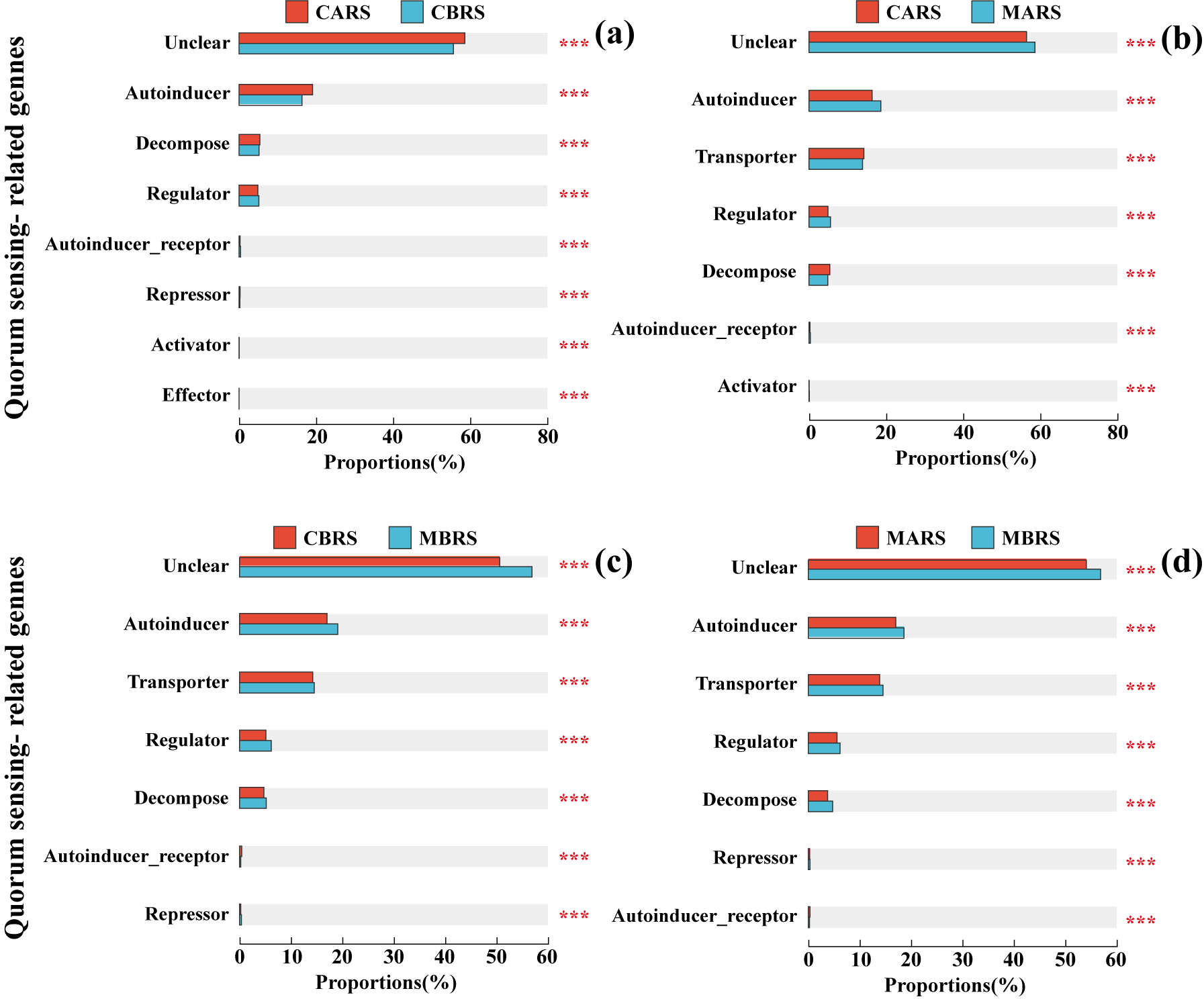


**Fig. S20.** Statistical analysis of bacterial quorum sensing (QS index) of both plants’ rhizosphere soil in control and mining areas.


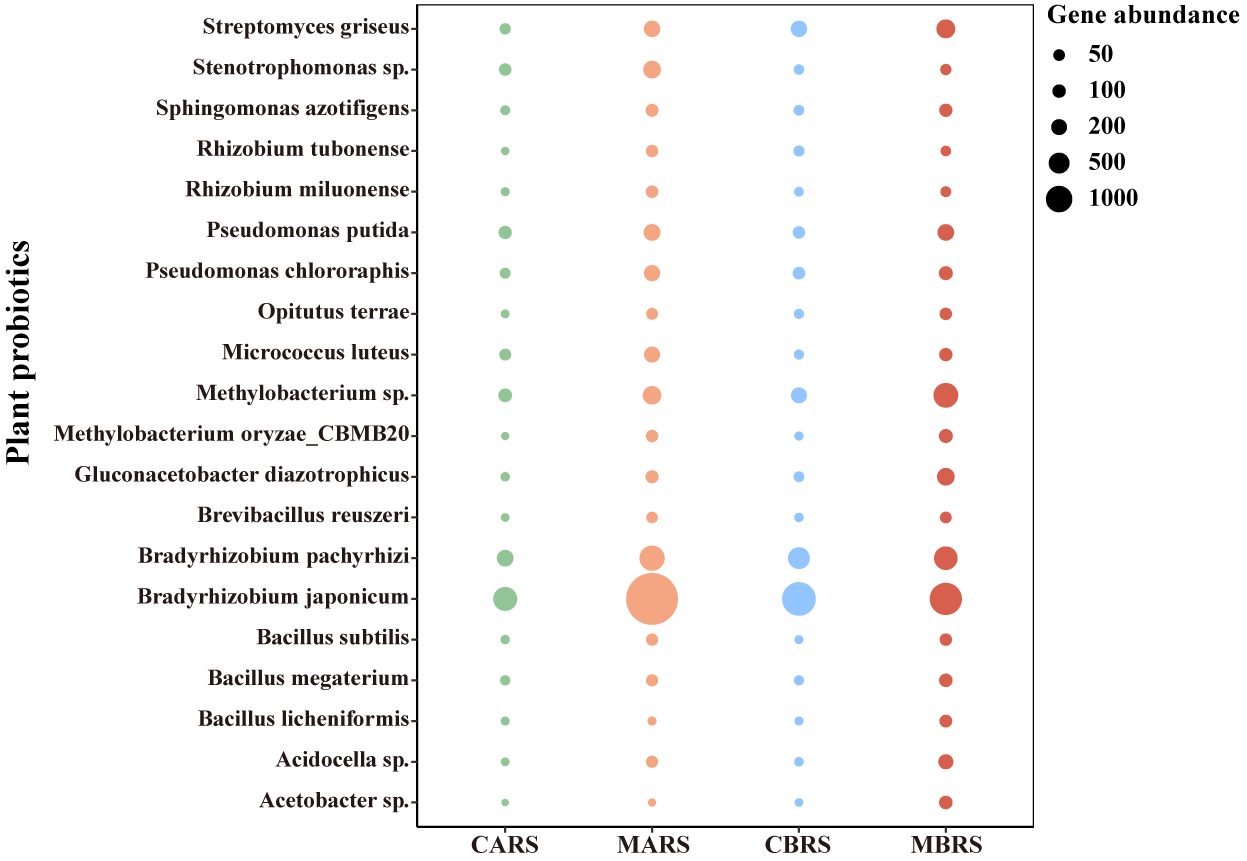


**Fig. S21.** Notes on probiotics of both plants’ rhizosphere soil in control and mining areas.

**Note:** We selected some plants growth-promoting bacteria from Probio's probiotic database. The figure displays the top 20 species enriched in the rhizosphere of plants in the mining area.


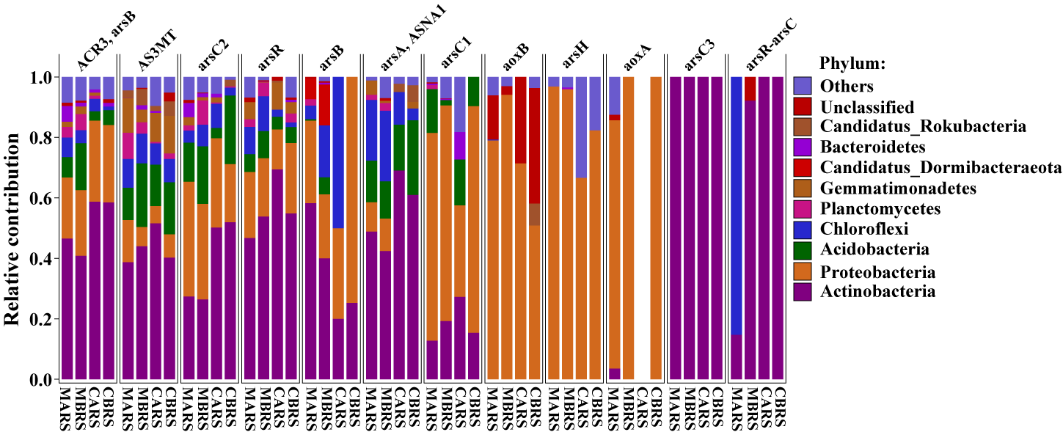


**Fig. S22.** Microbial contribution of Sb/As-resistance related genes of both plants’ rhizosphere soil in control and mining areas.


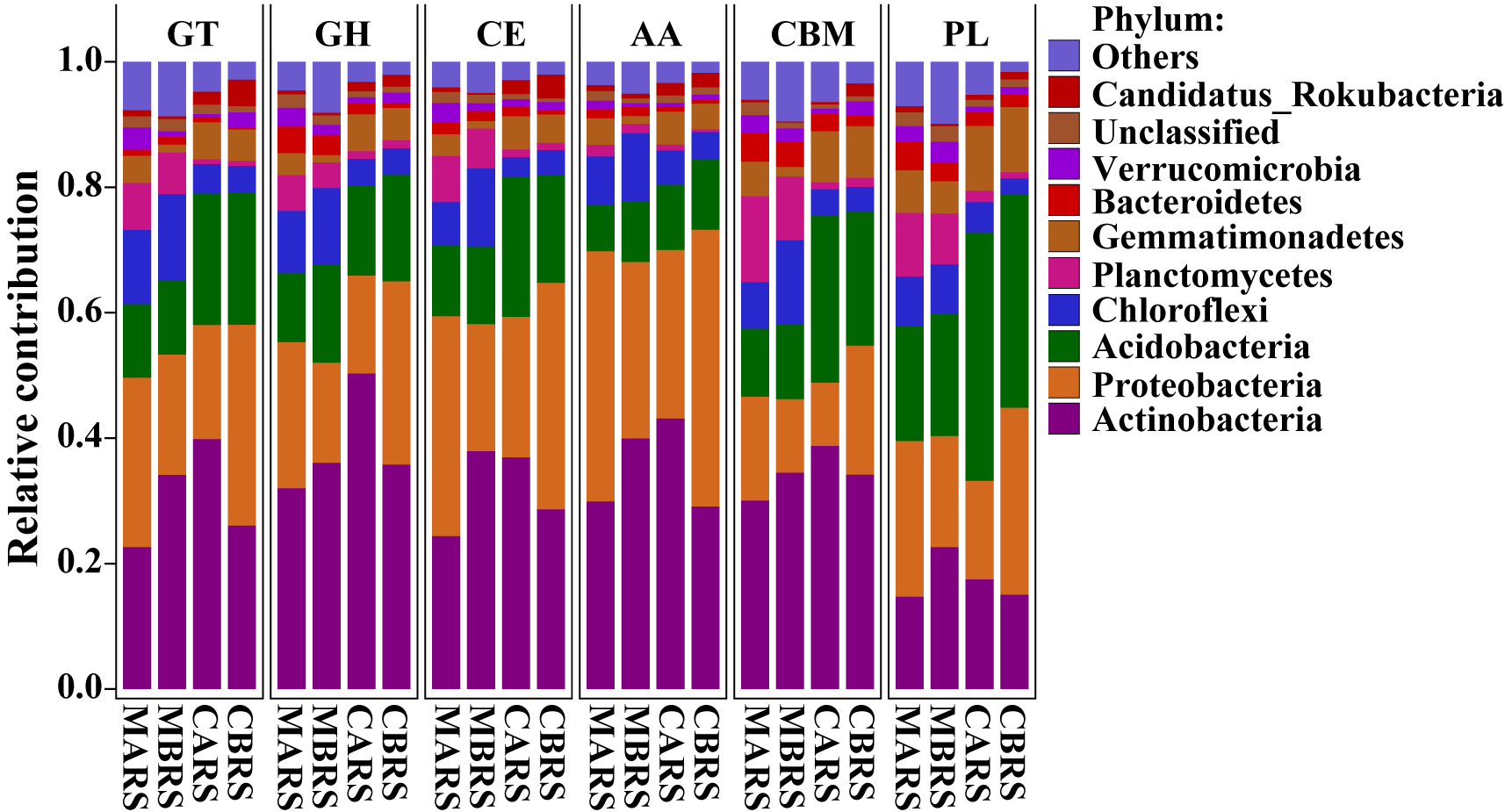


**Fig. S23.** Changes of genes abundance related to carbon cycle (a) and microbial contribution (b) of both plants’ rhizosphere soil in control and mining areas.


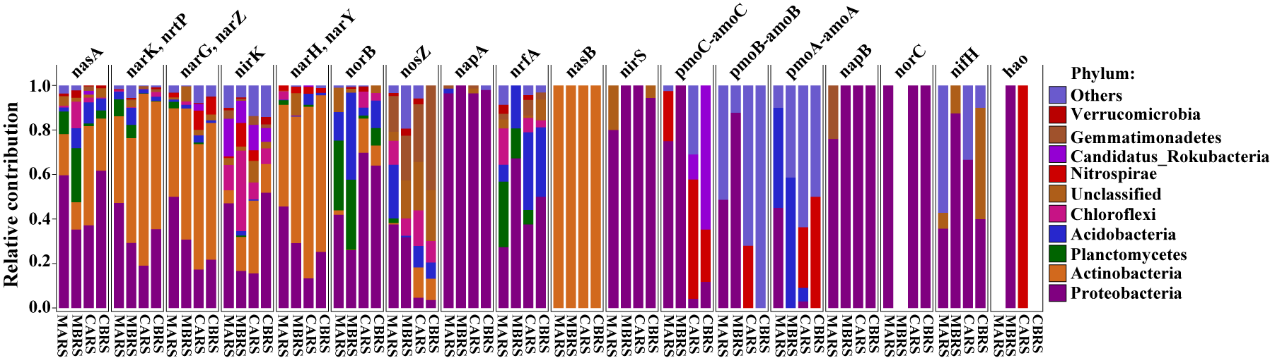


**Fig. S24.** Microbial contribution of nitrogen cycle-related genes of both plants’ rhizosphere soil in control and mining areas.


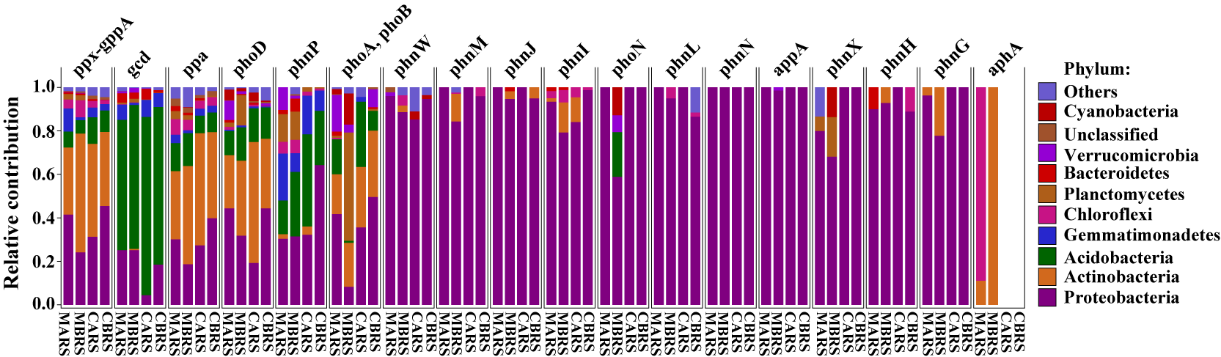


**Fig. S25.** Microbial contribution of phosphorus cycle-related genes of both plants’ rhizosphere soil in control and mining areas.


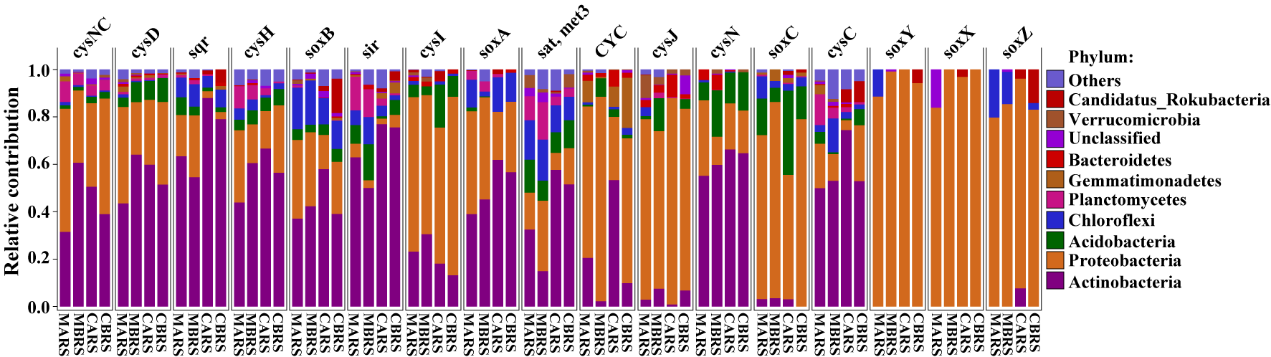


**Fig. S26.** Microbial contribution of sulfur cycle-related genes of both plants’ rhizosphere soil in control and mining areas.


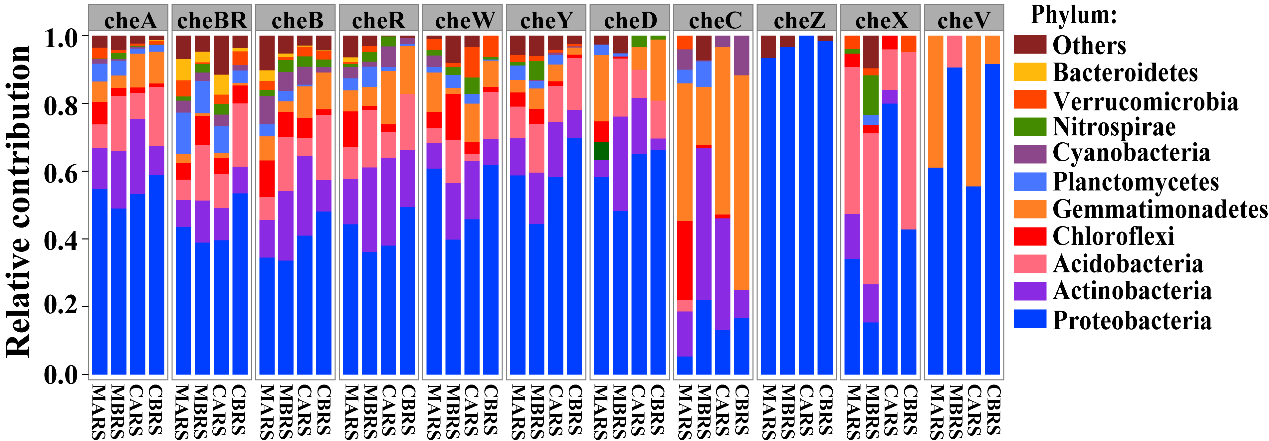


**Fig. S27.** Microbial contribution of chemotaxis system - related genes of both plants’ rhizosphere soil in control and mining areas.


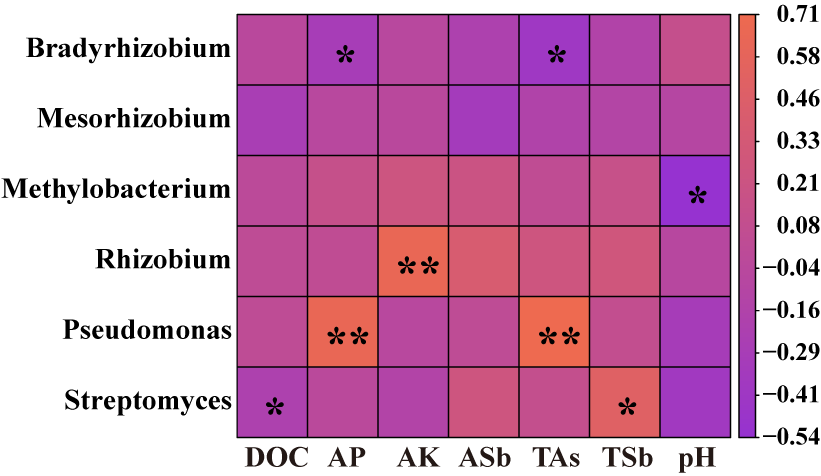


**Fig. S28.** Correlation analysis between plant probiotics and environmental parameters. The legend on the right indicates the pearson correlation coefficients.

**Note**: Due to different sequencing principles and taxonomic identification depths between smetabarcoding analysis and 16S rRNA gene sequencing, the taxonomic classification of these probiotics in the 16S rRNA gene sequencing results could only be traced back to the genus level at lowest resolution. DOC: dissoluble organic carbon, AP: available phosphorus, AK: available potassium, TSb: total antimony, TAs: total arsenic, ASb: available antimony. * is *P* < 0.05, ** is *P* < 0.01.


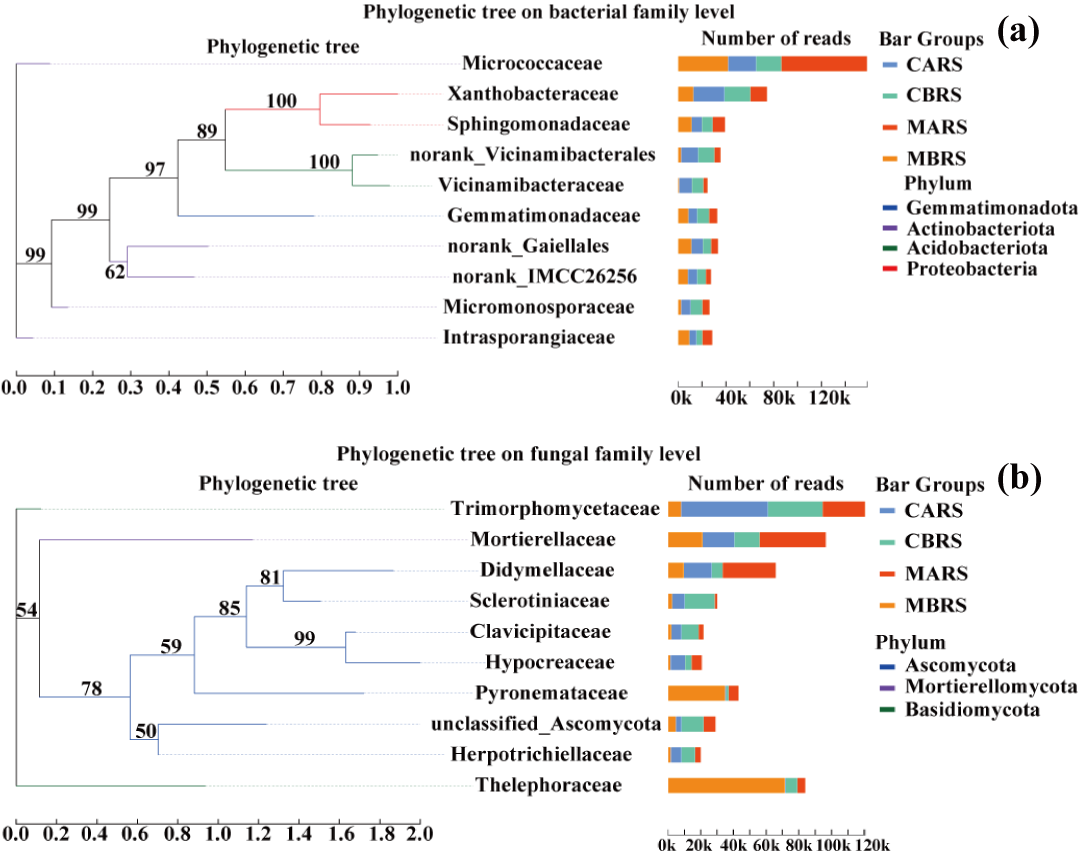


**Fig. S29**. Phylogenetic tree based on the evolutionary relationships among rhizosphere microbial species, revealing the phylogenetic affinity of native dominant plant rhizosphere microorganisms from a molecular evolution perspective (a and b).

**Note**: On the left is the phylogenetic tree of evolutionary relationships, where each branch represents a family (the top ten families by total abundance). Branches are color-coded based on the phylum level to which the species belong, and branch length represents the evolutionary distance between two species, i.e., the degree of difference between species. The bar chart on the right depicts the proportion of reads for each species across different plant rhizospheres.


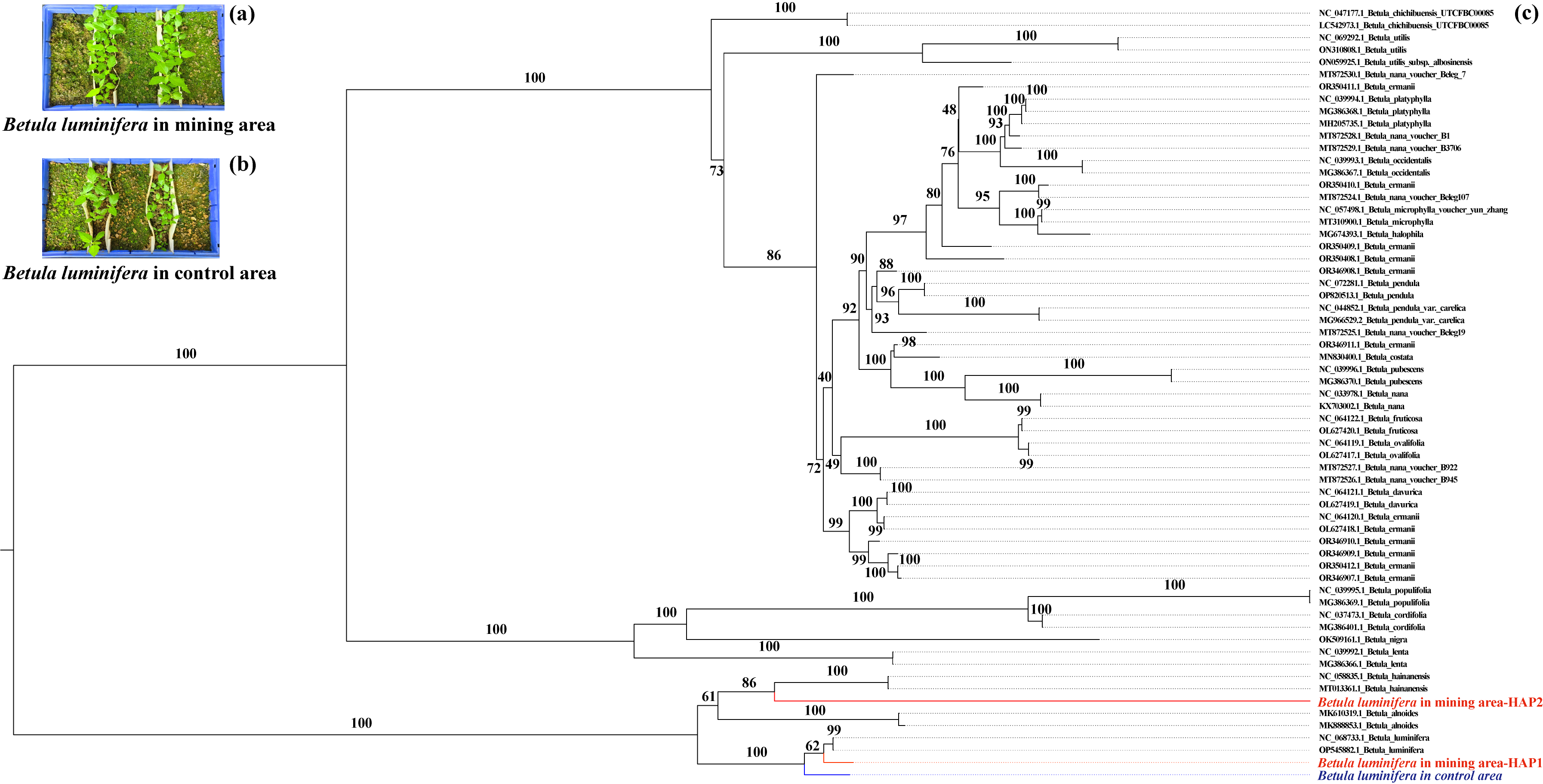


**Fig. S30**. *Betula luminifera* in mining area and control area (a and b), and phylogenetic tree of chloroplast genomes of *Betula luminifera* from the control and mining areas (c). Two haplotypes (HAP1 and HAP2) of *Betula luminifera* from the mining area are highlighted with red lines, while *Betula luminifera* from the control area is highlighted with blue lines.

**Note:** Seeds of *Betula luminifera* were collected separately from the control area and the mining area, and then sown in soil from the mining area. After 100 days, leaves of *Betula luminifera* were collected for whole-genome resequencing to detect mutations (a and b).
